# Prokaryote- and Eukaryote-Based Expression Systems: Advances in Post-Pandemic Viral Antigen Production for Vaccines

**DOI:** 10.3390/ijms252211979

**Published:** 2024-11-07

**Authors:** Nelli S. Khudainazarova, Dmitriy L. Granovskiy, Olga A. Kondakova, Ekaterina M. Ryabchevskaya, Angelina O. Kovalenko, Ekaterina A. Evtushenko, Marina V. Arkhipenko, Nikolai A. Nikitin, Olga V. Karpova

**Affiliations:** Department of Virology, Faculty of Biology, Lomonosov Moscow State University, 119991 Moscow, Russia; nelly.khudaynazarova@bk.ru (N.S.K.); dgran98@gmail.com (D.L.G.); olgakond1@yandex.ru (O.A.K.); eryabchevskaya@gmail.com (E.M.R.); angelina-kovalenko94@mail.ru (A.O.K.); trifonova.katerina@gmail.com (E.A.E.); armar74@mail.ru (M.V.A.); karpovaov@my.msu.ru (O.V.K.)

**Keywords:** expression system, vaccine development, recombinant protein, vaccine antigen, recombinant antigen, antigen design, recombinant vaccine platforms, virus-like particles, subunit vaccines, biopharmaceutical production

## Abstract

This review addresses the ongoing global challenge posed by emerging and evolving viral diseases, underscoring the need for innovative vaccine development strategies. It focuses on the modern approaches to creating vaccines based on recombinant proteins produced in different expression systems, including bacteria, yeast, plants, insects, and mammals. This review analyses the advantages, limitations, and applications of these expression systems for producing vaccine antigens, as well as strategies for designing safer, more effective, and potentially ‘universal’ antigens. The review discusses the development of vaccines for a range of viral diseases, excluding SARS-CoV-2, which has already been extensively studied. The authors present these findings with the aim of contributing to ongoing research and advancing the development of antiviral vaccines.

## 1. Introduction

The world faces the constant threat of new, or extremely evolved, versions of known viral diseases. The high rate of mutation in the genomes of some RNA-containing viruses, the recombination and reassortment in viruses with a segmented genome, the ability to overcome the interspecies barrier and the huge size of the reservoir of zoonotic viruses are just some of the factors that might facilitate new outbreaks of human viral infections with epidemic and/or pandemic potential [1]. The recent SARS-CoV-2 pandemic has taught the world a costly lesson about the devastating consequences that viral disease outbreaks can cause. This pandemic has served as a powerful catalyst for the development and introduction of new types of recombinant vaccines, as well as improvements in various technology platforms for the faster production of vaccine components [2]. Prophylactic vaccination against viruses that cause serious acute and chronic infectious diseases remains a key approach to reducing their spread and eliminating them in the future.

The technology for producing biopharmaceutical recombinant proteins, including vaccine antigens, is safe, scalable and well-established [3]. Generally, recombinant vaccines produced in prokaryotic or eukaryotic expression systems include subunit vaccines based on full-length viral antigen(s) or fragments/epitopes of those antigen(s); and vaccines based on virus-like particles (VLPs). In particular, VLPs vaccines against hepatitis B virus and human papillomavirus have been used for several decades, with proven safety [4]. Research into new vaccine candidates continues to improve their effectiveness and/or reduce their cost. The design of safer, more easily produced and more immunogenic antigens with higher protective properties, and the development of ‘universal’ antigens to create broad-spectrum vaccines against various strains/genotypes of viruses with great genetic diversity, are major challenges for modern vaccinology [5]. Selecting the optimal expression platform and optimal adjuvant (delivery system/immunostimulants) is also crucial when designing a new vaccine [6,7].

The current review examines various approaches and strategies used, in the five years since the SARS-CoV-2 pandemic, for the production of antigens for vaccines against various human viral diseases in expression systems of bacteria, yeast, plants, insects and mammals. The focus was on published data on preclinical and clinical trials for the period from 2019 to 2024. Some developments registered on the clinicaltrials.gov website https://clinicaltrials.gov/ (accessed on 1 November 2024) during this period were considered as well, if the information on the expression system used was provided. The main aim was to cover the diversity of technologies used for the antigen production. Thus, the vaccines considered were selected primarily based on their affiliation with different approaches used in their obtaining, rather than their objective relevance to the field of vaccine development. Vaccines against SARS-CoV-2 have deliberately not been included in the review, since they have recently been the focus of close attention and have been discussed in numerous reviews [2,8,9,10,11,12]. This review examines the main systems used for the expression of recombinant proteins, their advantages, limitations and application for the production of vaccine antigens. An analysis was also made of various vaccine antigen design strategies, with a particular focus on approaches to designing ‘universal’ antigens, adjuvant systems and technologies for producing various types of vaccine.

**Figure 1:**
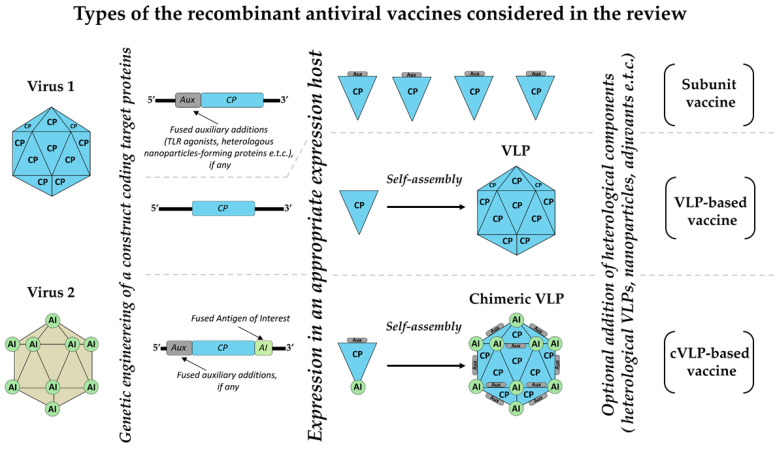
Types of the recombinant vaccines considered in the review. CP—Capsid Protein; AI—Antigen of Interest; Aux—Auxiliary additions to the antigen sequence; VLP—Virus-Like Particle; cVLP—Chimeric Virus-Like Particle.

The review’s data are presented in the form of summary tables, with vaccines being grouped into three major types: virus-like particles-based vaccines (VLPs), chimeric VLP-based vaccines (cVLPs) and subunit vaccines (subunit). The visual description of these types of vaccines is presented in Figure 1. In virus-like particles-based vaccines, the target recombinant antigens (specifically, capsid proteins) assemble into virus-like particles. In chimeric VLP-based vaccines, the target recombinant antigen is genetically fused to a supporting viral protein that mediates the assembling of the chimeric VLP. Subunit vaccines include all remaining vaccines that contain recombinant antigen as part of a formulation. The viral origin of VLP-forming proteins and the genetic fusion of the proteins are crucial for this classification. Even if the antigen is genetically fused to a supporting protein that mediates its assembly into an ordered structure, and this supporting protein does not have a viral origin, the vaccine was considered to be subunit. Similarly, the vaccine was considered to be subunit if the target antigen is bound to VLPs serving as an adjuvant and the binding is performed after the expression of the components using adaptor molecules and mechanisms other than genetic fusion. The authors hope that this review will be informative and helpful for researchers in the field of vaccinology.

## 2. Prokaryotic Expression System

*Escherichia coli* is the best-known, and a thoroughly researched, prokaryotic expression system. Despite ongoing studies into other bacterial expression systems, such as *Bacillus subtilis* [13], *Pseudomonas fluorescens* [14], *Pseudomonas putida* [15], *Ralstonia eutropha* [16] and *Lactococcus lactis* [17], *E. coli* remains the dominant bacterial species in use, due to its cost-effectiveness and because of the wide range of readily available genetic tools and strains that have been created to solve a variety of production tasks. *E. coli* is commonly the preferred choice for the production of prokaryotic proteins, although numerous eukaryotic proteins can also be effectively expressed in *E. coli*. It is generally believed, however, that *E. coli* is unable to provide most of the post-translational modifications that are essential for eukaryotic proteins, and it often misfolds complex proteins, such as those containing multiple disulfide bonds or several subunits. Until recently, expressing large proteins in *E. coli* was considered to be challenging and almost all *E. coli*-produced therapeutic proteins approved by the FDA had a molecular weight of less than 32 kDa [18]. Another issue associated with proteins expressed in *E. coli* is LPS (lipopolysaccharide, endotoxin) contamination. This necessitates the strict control of residual endotoxin content in therapeutic proteins [19], as there is no effective method to completely remove LPS [20].

Advancements in technology have, however, enabled engineered *E. coli* to overcome previous limitations and express a much broader range of proteins, through the integration and optimisation of novel molecular engineering tools and computational modelling [18]. The most common approach to expanding the capabilities of *E. coli* as an expression system is the use of specialised strains. For example, new mutant strains of *Escherichia coli* have been engineered that are capable of forming disulfide bonds in cytoplasm [21,22]. The construction of mutant *E. coli* strains that lack endotoxin is another remarkable achievement, which addresses the issue of endotoxin contamination in recombinant protein production [23,24]. Recently, Wang and colleagues reported the development of LPS-deficient strains for producing virus-like particles (VLPs) of nine papillomavirus genotypes for inclusion in a multivalent vaccine candidate [23]. In addition, progress has been made in solving the issue related to the size and complexity of the target recombinant protein. The successful expression of the vaccine antigen DBL1x-2x (multidomain of VAR2CSA, the leading antigen for malaria vaccine development), with a molecular mass of 100 kDa in *E. coli* (strain Shuffle), appears to be promising [25]. The developers achieved a high yield of correctly folded and functional vaccine antigen DBL1x-2x. A vaccine based on DBL1x-2x has demonstrated a high level of efficacy in preclinical studies [25] and is currently undergoing clinical trials [26]. Another major breakthrough is that recent pioneering efforts in *E. coli* glycoengineering have unlocked new possibilities for this expression system, such as the production of mammalian glycoproteins and antibacterial bioconjugate vaccines against human infectious diseases [27,28,29,30,31,32,33,34,35].

Among all procaryotic expression systems, *E coli* is currently the most widely used species for the production of recombinant viral antigens. Table 1 provides an overview of recent prophylactic vaccine viral antigens that have been successfully expressed in *E. coli* and then used in both licensed vaccines and candidate vaccines at various stages of preclinical and clinical trials.

### 2.1. Influenza Virus

It is well known that current influenza vaccines are mainly strain-specific and have limited efficacy against emerging influenza strains. The variability of the influenza virus surface proteins, haemagglutinin (HA) and neuraminidase (NA), necessitates the almost annual development of vaccines corresponding to current influenza strains. Vaccine efficacy against seasonal influenza ranges from 10% to 60%, reaching the lowest values when vaccine strains do not match circulating strains [36]. The development of a universal influenza vaccine capable of providing long-term protection against various influenza strains, including those with pandemic potential, remains a key priority [37]. Effective infection control can be achieved by developing broad coverage vaccines based on conserved antigens such as the HA stem domain and conserved epitopes of HA head, NA, M1, NP and M2e [36]. 

The extracellular domain of the M2 membrane protein (M2e) is known to be highly conserved in almost all human influenza isolates and is therefore often used as a vaccine base. The immunogenicity and safety of a universal influenza A vaccine candidate, ACAM-FLU-A, consisting of hepatitis B virus core (HBc) VLPs displaying conserved influenza virus M2e epitopes on the surface, was confirmed in phase I clinical trials. Despite demonstrating immunogenicity in the initial trial, further development of this vaccine has stalled, possibly due to a rapid decline in M2e-specific antibody titres over time [38]. Another universal influenza vaccine, Uniflu, containing four copies of M2e fused within the immunodominant HBc loop, demonstrated encouraging results in mice by reducing virus titres and increasing survival rates [39]. This vaccine candidate provided broad protective efficacy (against A/H1N1, A/H3N2, A/H2N2), but its extent will be determined in further studies. This candidate is currently undergoing evaluation in a single-site, randomised, double-blind, placebo-controlled clinical trial (NCT03789539), with results expected in the near future [36]. Further development of vaccine candidates utilising tandem copies of M2e epitopes includes chimeric VLPs derived from the capsid proteins of Macrobrachium rosenbergii nodavirus [40], bacteriophageT4 [41] and the hybrid flagellin/HBc platform [42]. The results of these studies have demonstrated the efficacy of chimeric VLPs in presenting tandem copies of M2e and protecting mice against infection. 

As the basis for a vaccine, epitopes of other conserved influenza A antigens can be used. For instance, universal subunit vaccine candidate Multimeric-001 (M-001) contains nine B- and T-cell epitopes (CD4+ and CD8+) from NP, HA and M1 that are conserved in both influenza A and B. M-001 is believed to induce an influenza-specific cellular immune response that is crucial for providing protection [43]. The safety and immunogenicity of M-001 as a stand-alone vaccine and as a pandemic primer have been reported previously [44,45]. Studies have indicated that M-001 can potentially serve as a primer for the seasonal trivalent inactivated vaccine (TIV), increasing antibody titres and extending the immune response to influenza strains beyond those covered by the TIV. In contrast to previous studies, the introduction of M-001 as a primer before administering quadrivalent inactivated influenza vaccine (IIV4) did not increase antibody titres to IIV4, although it did result in the expansion of the multifunctional CD4+ T cells subset over a 6-month period [46]. 

Recently, Zykova and colleagues developed and evaluated universal vaccine candidates based on conserved regions of M2e and HA2 (the second subunit of hemagglutinin). For antigen display, a self-assembled peptide (SAP) capable of oligomerising in vitro into icosahedral nanoparticles was used. This subunit vaccine candidate elicited a robust immune response and provided mice with a high level of protection against lethal influenza A infection [47]. To further improve vaccine efficacy and enhance the immune response, the T helper Pan DR binding epitope (PADRE) and conserved influenza virus NP epitopes were added to the vaccine. The inclusion of NP epitopes appears promising, as the NP protein is a key target for cross-reactive cytotoxic T lymphocytes (CTL) in response to influenza A viruses. The immunisation of mice with SAP-based nanoparticles displaying tandem copies of conserved epitopes M2e and HA2, NP and T helper epitope PADRE elicited high IgG titres against M2e and the entire virus (H3N2). The study also observed the formation of antigen-specific multifunctional CD4+/CD8+ effector memory T cells targetting both M2e and the entire virus. Although NP-specific effector memory T cells were not detected, probably due to suboptimal antigen design, immunisation with this vaccine conferred significant protection in mice against lethal influenza A infection [48]. 

### 2.2. Human Papillomavirus (HPV)

All licensed human papillomavirus (HPV) vaccines, whether produced in *E. coli* or yeast (Section 3), are based on VLPs formed by self-assembly of the L1 structural protein. In recent years, significant strides have been made in the development of *E. coli*-derived HPV vaccines. A highly promising advancement in this field is the approval and prequalification by the World Health Organisation (WHO), in 2021, of a bivalent vaccine Cecolin, that contains HPV-16 and HPV-18 recombinant L1 virus-like particles (VLPs) and aluminum hydroxide as an adjuvant. It was developed by Xiamen Innovax and licensed in China in 2019 (Table 1). Building on this success, the nine-valent vaccine (Cecolin 9 against HPV 6, 11, 16, 18, 18, 31, 33, 45, 52 and 58) was developed. It has recently completed phase II clinical trials, demonstrating good tolerability and immunogenicity [49]. Remarkable results from a randomised single-blind trial conducted in China revealed that Cecolin 9 induced a type-specific immune response against HPV that was no less than that of Gardasil 9 (Merck), expressed in yeast. This is a significant finding, since the cost of currently licensed HPV vaccines remains a barrier for many low- and middle-income countries. By offering a cost-effective alternative, Cecolin 9 may help address challenges in vaccine access and availability, ultimately contributing to the prevention of HPV-related diseases on a global scale [50].

Each vaccine consists of a mixture of VLP of different HPV genotypes, meaning that these vaccines are type-specific and provide only limited protection. At the same time, more than 400 HPV genotypes have been identified based on genome structure and tropism to human epithelial tissues [51]. Currently, 13 HPV types are classified as being high-risk and are considered human carcinogens. Unfortunately, further increasing the number of VLPs types in a vaccine may be problematic, due, in particular, to the high doses required for immunisation, leading to potential side effects and increased production costs. In response to this, Chinese researchers developed L1 VLP vaccines based on a consensus approach, in which a single VLP incorporates immunodominant epitopes from three HPV genotypes not yet covered (or partially covered) by existing vaccines. It has been demonstrated that such chimeric VLPs induce virus-neutralising titres that are comparable with those of a mixture of three single-genotype VLPs in both mice and nonhuman primates. Seven chimeric triple-genotype VLP human papillomavirus vaccines have already been characterised (Table 1) [52,53,54]. This approach may have promise for further development and application. 

Another strategy for developing a vaccine against HPV with a broad spectrum of action involves using conserved and consensus neutralising epitopes of the minor HPV capsid protein, L2. Unlike L1, L2 is incapable of self-assembly to form VLPs, which prompts the development of epitope-specific protein vaccines based on L2. To improve the efficacy of such epitope-specific vaccines, various approaches have been used, including epitope fusion to immunostimulatory molecules, utilising tandem repeats of the epitope, incorporating various external adjuvants and developing chimeric VLPs (Table 1) [55,56,57]. In a study conducted by Mashhadi and colleagues, a genetic construct encoding the conserved RG1 epitope of the HPV L2 protein was developed. The resulting antigen included a tandem repeat of RG1 fused to TLR4/5 agonists and a tetanus toxoid epitope. The immunisation of mice with such a vaccine antigen in various formulations revealed the critical role of the built-in adjuvants in eliciting a robust immune response against the RG1 epitope, which could be even further boosted by a combination of TLR7 agonist/alum-containing adjuvants [57]. 

### 2.3. Rotavirus

Several *E. coli*-expressed vaccines against group A rotavirus (RVA) are currently under development. They are mainly based on the VP4* RVA structural protein and its subunits, VP8* or VP5*. The most extensively researched of these is the trivalent injectable vaccine P2-VP8*, which includes three types of truncated VP8* proteins derived from predominant RVA genotypes (P[8], P[4], P[6]) [58]. VP8* of each genotype was genetically fused to the P2 epitope of a tetanus toxin to enhance immunogenicity. Although the P2-VP8* candidate reached phase III clinical trials, they were recently discontinued due to the failure to demonstrate a superior level of protective efficacy than existing live attenuated vaccines. Efforts are currently being made to identify reasons for the suboptimal performance of this candidate vaccine [59]. A possible explanation for the vaccine’s underperformance could be the mismatch between the sequences of the P2-VP8* antigens and actual circulating isolates [60]. To address this issue, alternative strategies are being explored. One such strategy involves obtaining VP8* and VP5* vaccine antigens through a consensus approach based on a wide range of circulating rotavirus isolates [61]. It enables the creation of antigens that closely match current RVA strains. This strategy was further enhanced by fusing the core antigen (consensus sequence VP8*P[8]) with a highly conserved neutralising epitope of RVA [62]. The resulting vaccine antigen was considered to be highly immunogenic in composition with plant virus particles used as a novel adjuvant and presentation platform. Another recent study introduced a method for developing a subunit RVA vaccine that utilizes pseudoviral nanoparticles (PVNPs) formed by the norovirus capsid protein as a platform [63]. The research created particles, S60-VP8* PVNPs, each displaying 60 copies of the VP8* antigen of P[8], P[4], P[6] or P[11] RVA genotype. Currently, Xia and colleagues are developing a trivalent subunit vaccine containing three types of S-VP4e PVNPs that presents ectodomains of the VP4 protein derived from P[8], P[4] or P[6] RVA genotypes [64]. Both vaccine candidates, S-VP4e PVNPs and S60-VP8* PVNPs, have induced a robust immune response in mice and stimulated the production of neutralising antibodies targetting P[8], P[4] and P[6] RVA genotypes. These advancements in vaccine design and development highlight ongoing efforts to improve the relevance and efficacy of RVA vaccines.

### 2.4. Zika Virus

The Zika virus (ZIKV) of the flavivirus genus poses a serious threat to public health, due to its recent global spread, recurring outbreaks and association with neurological developmental abnormalities in fetuses and Guillain-Barre syndrome in adults. To date, there are no approved vaccines against ZIKV infection. One of the main challenges in vaccine development is that, given close genetic and structural similarity, flaviviruses share highly conserved epitopes that can be recognised by cross-reactive antibodies. The presence of these antibodies can enhance the infection of heterologous, ADE (antibody-dependent enhancement) phenomenon, which is a major concern for vaccine use in areas where different flaviviruses co-circulate, such as regions with co-circulation of Zika and dengue viruses (DENV).

The envelope protein (E) of flaviviruses plays a pivotal role in vaccine development, as it is a crucial target for neutralising antibodies. Furthermore, it is believed that the EDIII domain of the E protein could provide an optimal basis for a ZIKV vaccine, as antibodies recognising EDIII are mostly specific to a particular flavivirus and/or serotype and are hence not capable of triggering ADE. In this regard, Cabral-Miranda and colleagues created a candidate vaccine based on the EDIII domain and virus-like particles of cucumber mosaic virus (CuMV) coat protein, both expressed in *E. coli* [65]. Despite the presence of VLPs as a platform, this vaccine should be considered subunit since the antigen is chemically conjugated to a separately obtained heterologous VPLs. In this research, the CuMV VLPs were modified with universal Th cell epitope (derived from tetanus toxin) and chemically conjugated with the vaccine antigen. When administered with the novel adjuvant (Di-oleoyl-phosphatidyl-serine), these chimeric VLPs induced a robust immune response even after a single immunisation. Elicited IgG antibodies were capable of neutralising ZIKV while not enhancing DENV infection, as demonstrated in vitro. Thus, the proposed vaccine candidate avoids the main problem associated with Zika virus vaccines, namely ADE of heterologous flavivirus infection.

### 2.5. Varicella Zoster Virus (VZV)

Varicella-zoster virus (VZV) is the infectious agent responsible for varicella, which primarily affects children and susceptible individuals. Following initial infection, VZV enters a latent state, potentially reactivating when T-cell immunity decreases in older, or immunocompromised, individuals and causing herpes zoster (HZ, shingles). Two herpes zoster (HZ) vaccines are currently available—the recombinant Shingrix vaccine (GSK, London, UK), approved by the FDA in 2017, and the live attenuated ZVL vaccine, licensed by the FDA in 2006. The antigen in the Shingrix vaccine is a shortened form of glycoprotein E (gE), one of the major antigens of VZV, produced in CHO cells. Shingrix induces a robust, significantly stronger and longer-lasting immune response (over 10 years) than the ZVL vaccine, and is also effective in preventing HZ (>90%) [66,67,68]. The vaccine is licensed for use as a two-dose regimen in adults aged ≥50 years and adults aged ≥18 years who are, or will be, at increased risk of HZ because of immunodeficiency or immunosuppression caused by a known disease or therapy [69]. Production in a mammalian expression system, however, implies a high cost for the vaccine, which may limit its widespread adoption [66]. Ongoing research efforts are focused on broadening the range of available recombinant vaccines against VZV and HZ. Chen and colleagues proposed several truncated forms of gE VZV as a basis for the subunit vaccine. For this purpose, genetically engineered constructs encoding gE with various C- or N-terminal truncations were created and examined to produce soluble gE in the *E. coli* expression system [70]. As a result, truncated gE (31–358 aa) was selected and obtained with a yield of 500 μg/g of bacteria after purification. The antigenic properties of the resulting protein, assessed using a panel of mAbs targetting both conformational and linear epitopes of the VZV protein gE, were comparable with the antigenic properties of the glycosylated form of gE previously produced in insect cells [71]. In parallel studies in mice, the truncated gE antigen was found to elicit humoral and cell-mediated immune responses comparable with those observed with the commercial vaccines Shingrix and live attenuated vaccine (vOka LAV) [70]. The truncated gE antigen obtained in *E. coli* demonstrates the great potential as a basis for a vaccine that prevents both HZ and primary VZV infection.

### 2.6. Hepatitis E Virus

The only licensed hepatitis E (HEV) virus vaccine (Hecolin^®^) expressed in *E. coli* is currently available for use in China, and more recently in Pakistan [72]. The vaccine antigen used in Hecolin^®^, p239 (368–606 a.a.), is a truncated form of ORF2 (1–660 a.a.) derived from genotype 1. In preclinical studies, however, Hecolin^®^ induced protective immunity against infections caused by genotypes 1 and 4, so it was suggested that this vaccine may confer cross-protection against various HEV genotypes. Nevertheless, this vaccine has not yet been submitted for prequalification by the World Health Organisation (WHO). Another vaccine based on VLPs from p179 antigen (439–617 a.a. ORF2) derived from genotype 4 has also demonstrated cross-protection against genotype 1 and 4 in rhesus macaques. This vaccine candidate is currently undergoing phase II clinical trials [73,74]. In humans, however, at least five HEV genotypes, including 1–4 and 7, can cause infection. Thus, further research is required to determine whether vaccine candidates could provide cross-protection against all these genotypes.

### 2.7. Respiratory Syncytial Virus (RSV)

Respiratory syncytial virus (RSV) causes severe respiratory illness, especially in young children, older people and people with a chronic disease. Vaccines against respiratory syncytial virus (RSV) have been under development for over 60 years. The first vaccine candidate that reached clinical trials was formalin-inactivated. Unfortunately, vaccinated children experienced enhanced severity of the disease (enhanced respiratory disease, ERD) after infection with a wild-type virus: 80% of vaccinated participants were hospitalised and two infants (aged 2 years) died [75]. Further vaccine development progressed slowly, as the potential for ERD had to be considered in the design of new vaccine candidates. The immunological mechanisms underlying ERD are still not fully understood, but it is hypothesised that the reason is improper conformation of the vaccine antigens, which leads to the induction of low-affinity antibodies that are incapable of neutralising the virus. Instead, these antibodies provoke inflammation and airway hyperreactivity due to the excessive stimulation of the Th2 response compared with natural RSV infection [76,77].

The respiratory syncytial virus (RSV) contains three primary surface proteins embedded in its lipid bilayer outer membrane: the fusion glycoprotein (F), attachment glycoprotein (G), and small hydrophobic protein (SH). F and G proteins are the main targets for neutralizing antibodies, and several promising developments are based on these proteins (Section 5.3, Section 6.7 and Section 7.1). However, despite intensive research, no licensed vaccine for prevention RSV is yet available for children [78]. The development is complicated not only by the risk of vaccine-associated enhancement of infection, but also by the short-lived immune response, characteristic of both vaccination and natural infection. This challenge has prompted scientists to develop novel immunization strategies. Therefore, the SH protein, another RSV membrane component, is being studied as a basis for a vaccine. Although SH protein is low-immunogenic and does not elicit neutralizing antibodies, studies suggest it can reduce viral replication in animal model through mechanisms not yet fully understood. Anti-SH antibodies may possibly act by activating resident alveolar macrophages and triggering ADCC (antibody-dependent cellular cytotoxicity) [79,80]. One of the most comprehensively studied development of this type, produced in the *E. coli* system, is the ectodomain of the SH protein (SHe) fused to pneumococcal surface protein A (PspA) as a carrier [81]. To increase the immunogenicity, the SHe sequence was repeated three times. One of the key advantages of this candidate is the nasal delivery, which allows for generating not only systemic immune response, but also local immune response at the primary RSV invasion site—the respiratory mucosa. For that, the vaccine employs a unique delivery system, a cationic cholesteryl-group-bearing pullulan nanogel (cCHP-nanogel), which has an affinity for the mucosa. The authors demonstrated the induction of SHe-specific IgG and IgA antibodies that effectively inhibited RSV infection in mice. Furthermore, vaccination following infection significantly reduced viral load. As predicted, anti-SHe antibodies did not show a direct neutralizing effect, they appeared to act through alternative mechanisms; specifically, Fc receptor-mediated immune responses, like ADCC, played a role. One of the important achievements of this development was that it proved to be safe in studies on vaccine-associated enhancement of the infection. In this way, the vaccine demonstrated immunogenicity and protective efficacy without triggering enhanced disease, making this innovative approach highly promising for RSV vaccine development.

**Table 1:** Summary of some vaccine viral antigens expressed in *E. coli* in licensed and candidate antiviral vaccines against human diseases for the period from 2019 to 2024.

Pathogen/Disease	Vaccine Antigen(s)/Type of Vaccine	Adjuvant	Vaccine Name/Developer	Status	References or Clinical Trial Identifier (NCT)	Year Reported or Regulatory Approved
**Influenza virus/Influenza**	Nine conserved B- and T-cell epitopes (CD4+ and CD8+) from NP, HA and M1 proteins of influenza A and B virus strains/Subunit	None	BiondVax Pharmaceuticals, Ltd., Jerusalem, Israel; Emmes Corporation, Rockville, MD, USA, etc.	Clinical, phase 2	[46]	2023
	Five copies of M2e epitopes (fused to norovirus capsid protein forming VLPs)/Chimeric VLPs	None	NvC-M2ex3/University Putra Malaysia, Selangor, Malaysia	Preclinical study/mice, protectiveness	[40]	2019
	Three tandem copies of M2e epitopes (fused to T4 phage Soc protein with subsequent formation of VLPs using Hoc^-^Soc^-^ T4 phage particles)/Chimeric VLPs	None	M2e-T4 VLPs/Huazhong Agricultural University, Wuhan, China; The Catholic University of America, Washington, DC, USA, etc.	Preclinical study/mice, protectiveness	[41]	2021
	Tandem copies of M2e (fused to flagellin/HBc protein (FH) forming VLPs)/Chimeric VLPs	Flagellin as a part of the antigen	FH-M2e VLP/University of Rhode Island, USA	Preclinical study/mice, protectiveness	[42]	2022
	Tandem copies of conserved M2e, HA2 epitopes (displayed on SAP-based nanoparticles)/Subunit	None	Institute of Bioengineering, Research Center of Biotechnology of the Russian Academy of Sciences, Moscow, Russia	Preclinical study/mice, protectiveness	[47]	2022
	Conserved NP (two epitopes), tandem copies of M2e, HA2 epitopes (displayed on SAP-based nanoparticles)/Subunit	Derinat *	Institute of Bioengineering, Research Center of Biotechnology of the Russian Academy of Sciences, Moscow, Russia	Preclinical study/mice, protectiveness	[48]	2023
**Human papillomavirus (HPV)/Cervical cancer**	L1 protein (types 16, 18)/VLPs	Aluminum hydroxide	Cecolin/Innovax, Xiamen, China	Licenced	[82]	2019
	L1 protein (9-valent; types 6,11,16, 18,31,33,45,52,58)/VLPs	Aluminum hydroxide	Cecolin 9/Xiamen University, Xiamen, China	Clinical, phase 2	[49]	2019
	L1 protein (9-valent; types 6,11,16, 18,31,33,45,52,58)/VLPs	No information	Beijing Health Guard Biotechnology, Inc., Beijing, China	Clinical, phase 3	NCT05668572	2022
	L1 protein (types 39/68/70)/Chimeric VLPs	Aluminum hydroxide	Xiamen University, Xiamen, China	Preclinical study/mice, neutralising activity of Ab	[53]	2022
	L1 protein (types 51/69/26)/Chimeric VLPs	Aluminum hydroxide	Xiamen University, Xiamen, China	Preclinical study/mice, neutralising activity of Ab	[54]	2022
	Tandem copies of RG1 epitope of L2 protein (fused to TLR4/TLR5 agonists and tetanus anatoxin epitope)/Subunit	TLR4/TLR5 agonists and tetanus anatoxin epitope as a part of the antigen; TLR7 agonist/alum	Islamic Azad University, Tehran, Iran; Pasteur Institute of Iran, Tehran, Iran	Preclinical study/mice, neutralising activity of Ab	[57]	2023
	Four epitopes of L2 protein (fused to the bacteriophage MS2 coat protein forming VLPs)/Chimeric VLPs	Cholera toxin (CT) and MPLA	Michigan Technological University, Houghton, MI 49931, USA	Preclinical study/mice, cross-protectiveness	[55]	2019
	Four epitopes of L2 protein (fused to the bacteriophage MS2 coat protein forming VLPs)/Chimeric VLPs	Aluminum hydroxide	Michigan Technological University, Houghton, MI 49931, USA	Preclinical study/mice, cross-protectiveness	[56]	2023
**Group A rotavirus/** **Gastroenteritis**	Truncated VP4 protein (genotypes P[8], P[6])/Subunit	Aluminum-based adjuvant	Xiamen University, Xiamen, China	Preclinical study/guinea pigs and rabbits, neutralising activity of Ab	[83]	2022
	VP8* protein (genotypes P[8], P[4], P[6], and P[11]; displayed on nanoparticles derived from norovirus S domain)/Subunit	Aluminum hydroxide	S_60_-VP8* PVNPs/University of Cincinnati College of Medicine, Cincinnati, OH, USA	Preclinical study/mice, neutralising activity of Ab	[63]	2022
	Truncated VP4 protein (genotypes P[8], [P4], P[6]; displayed on nanoparticles derived from norovirus S domain)/Subunit	Aluminum hydroxide	Trivalent S-VP4e PVNPs/University of Cincinnati College of Medicine, Cincinnati, OH, USA	Preclinical study/mice, protectiveness	[64]	2024
	Universal antigen URRA based on VP8* protein/Subunit	Plant virus-based spherical particles (SPs)	Lomonosov Moscow State University, Moscow, Russia	Preclinical study/mice, immunogenicity	[62]	2024
**Zika virus/Zika fever**	EdIII (displayed on VLPs derived from CuMV coat protein; chemical conjugation)/Subunit	Dioleoyl phosphatidylserine (DOPS)	University of Oxford, Oxford, UK; University of Bern, Switzerland; Artemis Bio-Support, The Netherlands; Bencard Adjuvant Systems, UK	Preclinical study/mice, neutralising activity of Ab	[65]	2019
**Varicella zoster virus (VZV)/Varicella, herpes zoster**	Glycoprotein E antigen (truncated)/Subunit	Aluminum hydroxide or AS01B	Xiamen University, Xiamen, China; Chinese Academy of Medical Sciences, Xiamen, China	Preclinical study/mice, immunogenicity, neutralising activity of Ab	[70]	2023
**Respiratory syncytial virus (RSV)/RSV infection**	Three repeats of SH protein ectodomain (SHe) genetically fused to pneumococcal surface protein A (PspA)/Subunit	cCHP-nanogel (cationic cholesteryl-group-bearing pullulan nanogel)	The University of Tokyo, Tokyo, Japan; Oita University, Oita, Japan; Chiba University-University of California San Diego Center for Mucosal Immunology, San Diego, CA, USA, etc.	Preclinical study/mice, immunogenicity, protectiveness	[81]	2023

* Derinat (sodium desoxyribonucleate) is licensed for human use in the Russian Federation as an immunomodulator.

## 3. Yeast Expression System

Yeast as an expression host combines the advantages of highly efficient prokaryotic platforms (growth speed and ease of genetic manipulation) with the ability to perform necessary post-translational modifications for proteins of eukaryotic viruses such as glycosylation, disulfide bond formation and proteolytic processing. Since the 1980s, the yeast *Saccharomyces cerevisiae* has been the most widely used protein expression system among eukaryotes. Low protein yields, plasmid instability, hyperglycosylation and production of hypermannosylated glycan structures have, however, limited the number of commercial products of *S. cerevisiae* on the market.

To address the shortcomings of *S. Cerevisiae*, other yeast species such as *Pichia pastoris* (now reclassified as two distinct organisms: *Komagataella pastoris* and *K. phaffii* [84]), *Hansenula polymorpha* and *Kluyveromyces lactis* have started to be widely used as expression platforms. In the context of *P. pastoris*, it is convenient to consider the advantages of those organisms for antigen production. In comparison with *S. cerevisiae*, *P. pastoris* is characterised by easier genetic engineering, higher protein expression levels, multi-copy genomic integration of foreign genes, reduced hyperglycosylation of protein products, higher levels of extracellular protein secretion and greater cost-effectiveness, and is suitable for the high cell density cultivation and multi-copy genomic integration of foreign genes [85,86]. New elements are constantly being added to the *P. pastoris* expression toolkit, such as various promoters (e.g., pAOX1, pGAP, pTEF1, pCAT1, pSDH) and signal peptides modulating the secretion of protein products. Protein folding and secretion pathways are also being optimised. For the production of heterologous proteins in *P. pastoris*, a common approach is the use of vectors capable of replication in *E. coli* that can also be maintained in *P. pastoris* by means of auxotrophy markers (e.g., HIS4, MET2, ADE1, URA3) or antibiotic resistance genes [85]. Along with expression system optimisation, much effort has been made to develop strains capable of humanised N-glycosylation, since glycosylation in yeast differs from glycosylation in mammals, which use more complex glycan structures [85,86,87].

The use of the yeast expression system has made it possible to create several widely used vaccines. Currently approved prophylactic VLP-based vaccines against hepatitis B virus (Engerix™ B, GSK, Wavre, Belgium [88]; Recombivax™, Merck & Co., Inc., Rahway, NJ, USA [89]; Heplisav-B^®^, Dynavax Technologies, Emeryville, CA, USA [90]; Heberbiovac-HB^®^, Heber Biotec S.A., La Habana, Cuba [91]) and human papillomavirus (Gardasil^®^, Merck & Co., Inc., Rahway, NJ, USA [92]; Gardasil^®^ 9, Merck & Co., Inc., Rahway, NJ, USA [93]; Walrinvax™, Shanghai Zerun Biotechnology Co., Ltd., Shanghai, China [94]) were obtained using three yeast species as expression hosts: *S. Cerevisiae*, *H. polymorpha* and *P. pastoris*. Building on this success, active research is being conducted in the field of recombinant vaccines based on yeast-derived viral antigens. Reviews by Kumar and colleagues [95], Srivastava and colleagues [96] provide an impressive list of vaccine antigens, expressed in different yeast species, that could potentially be used in the development of prophylactic vaccines against human diseases. Such a high number of directions of research suggests the great potency of the yeast platform [95,96]. A list of studies on yeast-derived vaccine antigens against various viral diseases for the period from 2019 to 2023 is presented in Table 2.

### 3.1. Influenza Virus

In 2019, Wang and colleagues described a technology for the effective production of the conserved domain HA2 of HA protein in the *P. pastoris* expression system that made it possible to achieve an antigen yield of 100 mg/L. The sequence of HA2 was based on H1N1, HA A/Brisbane/59/2007 [97]. This study compared the yield and immunogenicity of HA2 antigen expressed in yeast and mammalian cells (HEK293T) and examined the effect of glycosylation in different glycoforms on immunogenicity and protectiveness. The results obtained showed that all glycoforms of the HA2 antigen induced a cross-reactive protective response in mice, but the monoglycosylated form of HA2 expressed in *P. pastoris* in combination with C34 glycolipid as an adjuvant was demonstrated to induce the strongest virus-neutralising activity against heterologous strains of influenza virus and the broadest protection in mice. This suggests that the yeast expression system might provide appropriate tools for the development of a universal vaccine for preventing influenza pandemics.

### 3.2. Flaviviruses

The flavivirus genus includes human pathogenic viruses such as dengue (DENV), Zika (ZIKV), yellow fever (YFV), West Nile (WNV), tick-borne encephalitis (TBEV) and Japanese encephalitis (JEV), which continue to be a public health problem. There are still no effective and safe vaccines against DENV, ZIKV and WNV available. In addition, some of the currently available vaccines against YFV, JEV and TBEV are not completely optimal and might be improved. One of the main challenges to vaccine development is the antibody-dependent enhancement (ADE) phenomenon found in flaviviruses, which was partially described in Section 2.4 [98]. For example, seronegative individuals vaccinated with a licensed quadrivalent live attenuated DENV vaccine, Dengvaxia, have an increased risk of developing severe dengue in the event of secondary infection [99]. The WHO Global Advisory Committee on Vaccine Safety currently recommends that Dengvaxia should only be administered to recipients who have been previously infected with wild dengue virus [100]. Moreover, it has been suggested that antibodies to ZIKV may increase DENV infection.

Shanmugam and colleagues developed a vaccine candidate against ZIKV. The vaccine was based on chimeric VLPs assembled from an in-frame fusion of four copies of ZIKV envelope domain III (EDIII) with the hepatitis B virus (HBV) surface antigen (HbsAG) and individual HbsAG [101] co-expressed in *P. pastoris*. The EDIII domain of ZIKV was chosen as the vaccine antigen because EDIII-specific antibodies appear to lack appreciable ADE potential in vivo [102]. Chimeric VLPs were obtained according to a previously published method for EDIII DENV presentation [102]. The antibodies elicited as a result of the immunisation of BALB/C mice were able to neutralise ZIKV reporter virus particles in cell culture, despite the low antibodies titres in sera collected. Moreover, chimeric VLP-induced antibodies did not enhance a sublethal DENV-2 (DENV serotype 2) challenge in AG129 mice, despite the presence of low levels of cross-reactive IgGs to DENV-2. As a possible way to enhance the immunogenicity of the VLPs, it is proposed to increase the number of EDIII copies within the EDIII-HbsAG fusion. However, this is known to complicate the assembly of VLPs. In this regard, the use of other adjuvants may be considered to create a highly immunogenic EDIII-based vaccine.

Yang and co-authors reported a candidate VLP vaccine against JEV based on a chimeric recombinant prM/Env protein. The prM/Env protein expressed in *P. pastoris* contained a truncated structural E protein (1–456 aa) preceded by the C-terminal 33 amino acids of the prM protein (135–167 aa) to ensure the proper post-translational processing of the E protein. Such design is necessary, since prM structural protein is closely associated with E protein and acts as a chaperon to promote E maturation. VLPs assembled from prM/Env protein induced a strong humoral and cellular immune response in mice and pigs and conferred complete protection against JEV infection in immunodeficient mice [103].

Seesen and colleagues reported the development of a bivalent nanoparticle-based subunit dengue vaccine, which is a mixture of two recombinant DENV-2 antigens, EDIII and the N-terminal fragment (1–279 aa) of nonstructural protein 1 (NS1), expressed in *P. pastoris* and encapsulated within N-trimethyl chitosan nanoparticles (TMC NP). The EDIII + NS1 TMC NP vaccine candidate was shown to induce anti-NS1 and anti-EDIII IgGs and specific CD4+ and CD8+ T-cell responses, which could not only neutralise infectious DENV-2 but also eliminate DENV-2-infected cells [104]. Development by Seesen and co-authors was based on their previous research, which demonstrated the ability of a similar vaccine candidate not containing NS1 protein to elicit neutralising antibodies with undetectable ADE activity [105]. It is suggested, however, that utilising NS1 as an additional immunogenic component of a vaccine may provide cross-protection against all variants of DENV, since NS1 is highly conserved among DENV serotypes. When using NS1 as a vaccine component, the truncation of the C-terminus of NS1 is required, since antibodies to it are cross-reactive with some self-antigens and hence may cause autoimmunity. It has previously been demonstrated that immunisation with NS1 with truncated C-terminus can reduce the production of cross-reactive antibodies. Therefore, the EDIII + NS1 TMC NP formulation might be safe in terms of immunopathological effects but it is still necessary to investigate the safety of the proposed vaccine candidate [104].

### 3.3. Hepatitis E Virus

Although a vaccine named Hecolin^®^, based on VLPs from p239, a truncated form of hepatitis E virus ORF2 protein (aa 368–606) expressed in *E. coli*, has been licensed in China (Section 2.6), there is still no WHO-approved prophylactic vaccine against hepatitis E. A vaccine based on VLPs assembled from p495 antigen, another truncated form of ORF2 (aa 112–608), manufactured by GlaxoSmithKline, in insect cells with a baculovirus expression system, was the first vaccine candidate to successfully pass clinical trials, demonstrating safety and efficacy. After phase II clinical trials, however, this project was discontinued, maybe due to the lack of commercial value [74]. Despite this, p495 is still of interest as an antigen. Gupta and colleagues produced a recombinant antigen p495 in the *P. pastoris* expression system. The antigen was secreted into the culture medium as an N-linked glycoprotein and the ability of the purified p495 protein to form VLPs was shown. The resulting VLPs were able to induce a significant immune response in mice [106,107]. It is suggested that the *P. pastoris* expression system may be a cost-effective alternative to the baculovirus expression system for hepatitis E virus VLP production [106].

**Table 2:** Summary of some vaccine viral antigens expressed in yeast in licensed and candidate vaccines against human diseases for the period from 2019 to 2023.

Pathogen/Disease	Vaccine Antigen(s)/Type of Vaccine	Adjuvant	Host Expression	Vaccine Name/Developer	Status	References or Clinical Trial Identifier (NCT)	Year Reported or Regulatory Approved
**Influenza virus/Influenza**	HA2 protein/Subunit	C34 glycolipid	*P. pastoris*	School of Life Sciences, Sun Yat-sen University, Guangzhou 510006, China; Firstline Biopharmaceuticals Corporation, Redmond, WA, USA	Preclinical study/mice, cross-protectiveness	[97]	2019
**Human papillomavirus (HPV)/Cervical cancer**	L1 protein (types 16, 18) forming VLPs/VLPs	No information	*P. pastoris*	Walrinvax/Yuxi Zerum Biotechnology, Yuxi, China	Licensed	[94]	NMPA * 2022
	L1 protein (9-valent; types 6,11,16, 18,31,33,45,52,58)/VLPs	No information	*P. pastoris*	Zerun HPV-9/Shanghai Zerun Biotechnology Co., Ltd., Shanghai, China	Clinical, phase 3	NCT05580341	2022
	L1 protein (4-valent; types 6, 11, 16, and 18) VLPs	No information	*H. polymorpha*	Shanghai Bovax Biotechnology Co., Ltd., Shanghai, China	Clinical, phase 3	NCT05584332	2022
	L1 protein (11-valent Types HPV6/11/16/18/31/33/45/52/8/59/68)/VLPs	No information	*H. polymorpha*	National Vaccine and Serum Institute, Beijing, China	Clinical, phase 3	NCT05262010	2022
**Zika virus/Zika fever**	EDIII (fused to HBsAg forming VLPs)/Chimeric VLPs	Alhydrogel	*P. pastoris*	Recombinant Gene Products Group, Molecular Medicine Division, International Centre for Genetic Engineering & Biotechnology, New Delhi, India	Preclinical study/mice, neutralising activity of Ab	[101]	2019
	EDIII/Subunit	Alhydrogel	*P. pastoris*	Institut Pasteur of Shanghai, Chinese Academy of Sciences, University of Chinese Academy of Sciences, Shanghai, China	Preclinical study/mice, neutralising activity of Ab	[108]	2019
**Japanese encephalitis virus/Japanese encephalitis**	prM-Env antigen/VLPs	None	*P. pastoris*	College of Animal Science and Technology, Shihezi University, Shihezi, China	Preclinical study/pigs, neutralising activity of Ab	[103]	2022
**Dengue virus (DENV)/Dengue fever**	NS1 (1–279 aa) and EDIII of DENV-2 (encapsulated in the nanocarriers—TMC NPs nanoparticles)/Subunit	Trimethyl chitosan nanoparticles	*P. pastoris*	Mahidol University, Bangkok, Thailand	Preclinical study/mice, serotype-specific neutralising activity of Ab	[104]	2023
**Hepatits E virus (HEV)/Hepatits E**	HEV capsid protein ORF2 (112–608 aa; genotype 1) /VLPs	Aluminum-based adjuvant	*P. pastoris*	Virology Laboratory, Vaccine and Infectious Disease Research Centre, Translational Health Science and Technology Institute, NCR Biotech Science Cluster, Faridabad, India	Preclinical study/mice, immunogenicity	[106,107]	2020, 2022

* approved by the China National Medical Products Administration (NMPA).

## 4. Plant Expression System

The field of molecular farming, which involves using plants for protein expression, has advanced significantly, evolving from a promising platform to a reliable and established technology. Plants offer benefits for protein expression, such as cost-effectiveness, ease of scalability, the availability of eukaryotic-type post-translational modifications for proteins of eukaryotic viruses and the absence of common animal pathogens, ensuring the safety of plant-derived proteins. Nevertheless, there remain challenges that need to be addressed, such as low levels of protein expression, unique glycosylation patterns in plants and some issues related to regulatory approval. There are two ways to produce recombinant protein in plants: stable transformation involving the integration of the desired gene into the nuclear or plastid genome, and transient expression in which the gene is temporarily introduced into the plant cells. Obtaining stable transgenic plants is a complex, time-consuming and labour-intensive process. Furthermore, protein yields from nuclear transformation are usually low. It is believed that genetic transformation of the chloroplast genome can significantly increase the expression level of the target protein [109] but there are only a few plant species for which efficient transformation protocols have been successfully developed [110]. Transient transformation, in contrast to stable transformation, enables the rapid production of large quantities of recombinant proteins, making it a valuable tool for producing vaccine antigens [111]. For transient protein expression, the use of specific vectors is required; typically, these vectors are delivered to plants through agroinfiltration of the soil bacterium *Agrobacterium tumefaciens*. These vectors are commonly binary and capable of replication in both *E. coli* and *A. tumefaciens*. The expression cassette of such vectors can either comprise a nonreplicating sequence including a promoter, a target gene and a terminator, or a self-replicating phytoviral genome that can generate infectious progeny capable of systemic infection of plants, resulting in higher levels of protein production. The most widely used vectors, to date, are based on RNA-viruses, in particular tobamoviruses, potexviruses and comoviruses [112,113], and DNA-viruses such as geminiviruses [114,115]. Although the technology of agroinfiltration has been adapted to many plant species [116], *Nicotiana benthamiana* remains the primary choice for protein production [111], resulting in most of the progress being made on this particular plant species.

Deconstructed viral genomes containing only the essential components required for efficient protein expression, (promoters, terminators, translation enhancers and other regulatory sequences), have become widespread. Transient expression systems using viral vectors are constantly being developed to obtain the highest yield of the target protein. Among the innovative advancements in deconstructed vectors is a set of provector modules designed by Icon Genetics (Halle, Germany) under the trade name of magnICON^®^ technology, based on the genomes of two tobamoviruses forming functional RNA replicons when delivered into plant cells [117]. Using this system, VLPs of the hepatitis B virus core antigen (HBcAg) were obtained in *N. benthamiana* with a HBcAg yield of 2.38 mg/g fresh weight (FW) [118]. Another development based on magnICON^®^ technology has been a recombinant bivalent vaccine containing VLPs obtained in vitro from the norovirus capsid protein (VP1) of two norovirus strains [119,120]. Both VP1 types were produced in *N. bentamiana*. This vaccine candidate is currently undergoing clinical trials. The magnICON^®^ vector system has been shown to be adaptable, even for the large-scale production of pharmaceutical proteins [121]. Another promising development in this field is a ‘launch vector’ based on the TMV (Tobacco mosaic virus) genome [122]. Fraunhofer USA Center for Molecular Biotechnology (FhCMB) used this vector system for the efficient expression of vaccine antigens in plants; in particular, it has been used to create a subunit vaccine candidate against the influenza virus based on the hemagglutinin protein [123,124]. This vaccine candidate has successfully passed phase I clinical trials, demonstrating both safety and immunogenicity. Recently, Mardanova and colleagues reported a novel vector pEff, based on the genome of potexvirus PVX (potato virus X) [125], which provided a very high level of protein expression due to its ability to self-replicate in plant cells, the presence of a translation enhancer, a signal sequence for targetting recombinant protein to the endoplasmic reticulum and an expressing cassette for the P24 silencing suppressor from grapevine leafroll-associated virus-2. Subsequently, using pEff vector, chimeric Hepatitis E VLPs presenting the M2e peptide of influenza A virus were obtained with a yield of 200 μg/g FW after purification [126]. Several other deconstructed vector systems were developed based on the cowpea mosaic virus (CPMV) genome, such as pEAQ, pEAQ-HT [127] and pHREAC [128]. These technologies have been used to produce HIV gp140 antigen [129], chimeric VLPs vaccine candidates against dengue virus [130] and hepatitis E virus [131]. Vectors based on DNA-viruses, such as geminivirus BeYDV (bean yellow dwarf virus), are also being studied [132]. In particular, the BeYDV vector has been utilised to produce vaccine antigens for the prevention of dengue virus [133], Zika virus [134,135] and papilloma virus [136]. All of them were expressed in *N. benthamiana*. The latest developments using this plant species for vaccine antigen production are summarised in Table 3. *N. benthamiana* is applied not only to express individual antigens for inclusion in subunit vaccines, but also to generate VLPs and chimeric VLPs displaying target peptides on their surface, as shown in Table 3.

### 4.1. Influenza Virus

The development of influenza vaccines is challenged by the need for rapid production of those that target current influenza strains, as highlighted in Section 2.1. Transient expression in plants offers a solution to this issue, enabling the vaccine antigen to accumulate in large quantities within a short period of time. Furthermore, plants are considered to be optimal hosts for producing influenza VLPs containing only hemagglutinin (HA), as plant cells do not synthesise sialic acid and the presence of neuraminidase is not required for VLPs budding from the plasma membrane of plant cells. Medicago Inc. (Quebec City, QC, Canada), a company specialising in plant expression systems since 1999, has produced purified influenza virus HA VLPs in *N. benthamiana* and reported that it takes only 18 days after receiving the HA sequence of the target influenza strain [137]. The quadrivalent influenza vaccine candidate developed by Medicago Inc. has passed all three stages of clinical trials [138,139,140]. The vaccine induced hemagglutinin inhibition titres and provided protection against influenza in adults of all ages comparable with commercially available seasonal influenza vaccines. Unfortunately, Medicago Inc. ceased its operations in 2023, due to the termination of investments by the parent company (Mitsubishi Chemical Group, Tokyo, Japan), which raised some concerns [141,142,143]. The International Society of Molecular Plant Agriculture does not, however, consider this closure to be a serious argument against this technology, and it should not significantly affect future efforts [141].

Kentucky BioProcessing/iBio (2020) announced a phase I clinical trial of another quadrivalent plant-based subunit influenza vaccine, KBP-V001 (NCT04439695) (Table 3). Details have not yet been published, but Royal and colleagues reported that KBP-V001 consists of an equal weight mixture of four types of TMV-haemagglutinin chemical conjugates [144]. The use of phytoviruses, particularly TMV, as both immune stimulant and platform for antigen presentation is currently an active direction of development [145]. This technology provides an efficient system for the creation of vaccine candidates, adding to the range of tools against recurrent pandemics. A major limitation of chimeric VLPs that present heterologous epitope(s) is the potential reduction in assembly efficiency, which can result in low yields. This problem occurs when fused constructions of the antigen with the phytoviral envelope protein are used, which significantly restricts the size of possible insertions, (often short linear peptides). An alternative approach is chemical or enzymatic conjugation of the vaccine antigen after the assembly of recombinant (modified for conjugation) phytoviruses has been completed.

### 4.2. Rotavirus

It is widely known that the simultaneous expression of rotavirus structural proteins VP2, VP6 and VP7, with or without VP4, leads to the formation of triple-layered VLPs (2/6/7-VLPs or 2/4/6/7-VLPs) resembling infectious triple-layered rotavirus particles, although the production of 2/4/6/7-VLPs is challenging, due to the low yield of such particles [146]. Rotavirus VLPs have been obtained in various expression systems [146,147] but only one VLP-based vaccine has reached clinical trials. This monovalent vaccine candidate was produced in *N. bentamiana* through the co-expression of three structural proteins, VP2, VP6 and VP7 (G1 genotype), and the nonstructural protein NSP4 added to increase VLPs yield. The VP7, VP6 and VP2 proteins were under the control of the CPMV 160 expression system, whereas NSP4 was under the control of the CPMV HT expression system. The vaccine was well tolerated, at various doses, but induced only homotypic immune response in infants. Cross-protection against the globally common genotypes G2P[4], G3P[8], G4P[8], G9P[8] and G12P[8] was not reported [148].

### 4.3. Hepatitis B Virus

Over the past 40 years, licensed hepatitis B vaccines based on the small surface (envelope) protein HBsAgS expressed in yeast (Section 3) have demonstrated effectiveness in preventing infection. Research on HBV vaccines is, however, ongoing, with new vaccines being developed based not only on the small antigen (HBsAgS; S), but also on the middle (HBsAgM; M) and large (HBsAgL; L) antigens. S, M and L proteins are encoded by a single open reading frame, S-ORF, divided by three start codons into the pre-S1, pre-S2 and S domains, meaning that S, M and L proteins have the same C-terminus but different N-terminal extensions. The L protein consists of preS1, preS2 and S domains, and the M protein consists of preS2 and S domains [149]. The ratio of L, M and S proteins in the envelope of a mature virion is 1:1:4 [150]. By incorporating all three envelope proteins into the vaccine, the developers aim to provide comprehensive protection against HBV, targetting multiple epitopes that can enhance and broaden the immune response. The licensing of a novel vaccine, containing L, M and S proteins derived from mammalian cells, demonstrates an increased interest in producing vaccines of this type as an alternative to existing S protein-based vaccines [151,152]. In studies by Dobrica, Pantazica and colleagues, chimeric VLPs were obtained from the S protein and various preS1 epitopes of the L protein in both plant and mammalian expression systems [153,154,155]. Among the resulting chimeric proteins, the antigen S/preS116-42, expressed in mammalian cells, showed the highest expression level, immunogenicity and the ability to produce neutralising antibodies against both wild-type and vaccine variants of HBV [155]. Since the large-scale production of vaccine antigens in mammalian cells is quite expensive, *N. benthamiana* was chosen as a cost-effective alternative for S/preS116-42 expression [156]. It is believed, however, that plant glycosylation has specific features that may pose a problem for the immunogenicity of plant-derived vaccine antigens. In particular, unlike mammalian cells, plants synthesise complex N-glycans with β-1,2- xylose and core α-1,3-fucose, while sialic acid and core α-1,6-linked fucose are absent. To address this issue, multiplex genome editing using CRISPR/Cas9 was recently applied to create the FX-KO line of *N. benthamiana* for the production of recombinant proteins completely devoid of core α-1,3-fucose and/or β-1,2-xylose residues [157]. In the study of Pantazica and colleagues, the molecular, antigenic and immunogenic properties of the proteins produced in FX-KO and in wild-type *N. benthamiana* were compared [156]. Predictably, FX-KO-derived antigen S/preS116-42 demonstrated enhanced binding to a panel of conformation-dependent anti-S antibodies, reaching a level of recognition comparable with that observed for antigen derived from mammalian cells. Immunisation with S/preS116-42 from the FX-KO line also elicited significantly higher antibody titres in mice and, furthermore, these antibodies demonstrated more potent neutralising activity against native HBV than the antigen produced in wild-type plants [156]. This work confirms that glycoengineered *N. benthamiana* with ‘humanised’ N-glycosylation may be an improved host for expressing vaccine glycoproteins. Overall, the ability to modulate glycosylation patterns in plants opens up new possibilities for optimising the immunogenic properties of recombinant antigens, ultimately advancing the development of novel plant-based vaccines against various infectious diseases.

### 4.4. Hepatitis C Virus

The development of a vaccine against hepatitis C (HCV) is a challenging task, due to the genetic diversity of HCV strains (there are eight known genotypes and over 100 subtypes) and the virus’s ability to circumvent the immune response, using various strategies. Despite enormous efforts, a vaccine has not yet been created, but the scientific community remains hopeful of success. Researchers’ attention is focused on the two transmembrane envelope glycoproteins of HCV—E1, E2 and their heterodimer E1/E2, stabilised by disulfide bonds. As the basis for a vaccine, recombinant E2 and E1/E2 are primarily considered. Strategies used by viruses to evade the immune response include the presence of a hyper-variable region 1 (HVR1) in the E2 protein, which screens the more conserved E2 epitopes that are crucial for receptor binding. Shielding of these epitopes is also achieved by the glycosylation of E2, which contains 11 highly conserved N-linked glycosylation sites. In the context of vaccine development, a key point is that the presence or absence of these specific glycosylation sites can directly affect virus neutralisation. In some cases, glycans may be essential for interaction with neutralising antibodies, while in other cases differences in glycosylation patterns due to glycan shift mutations may alter or prevent neutralisation [158].

Modern vaccine development approaches focus on improving the immunogenicity of potential vaccine antigens, their correct folding and proper processing in various expression systems [159]. Molecular manipulations regarding antigen conformation and N-glycosylation pattern, including the removal of N-glycans that shield conservative epitopes, could be the key to developing an effective vaccine based on the E2 glycoprotein. Dobrica and colleagues successfully expressed full-length E2 glycoprotein in *N. benthamiana* [160]. To target E2 to the endoplasmic reticulum, the genetic construct of the recombinant E2 was complemented with the C-terminal domain (32 codons) of the E1 protein providing a signal sequence for cellular peptidase. As a result, plant-derived E2 was correctly processed and acquired the native conformation essential for binding to the cellular receptor, mediating virus entry into the host cell. Comparative analysis of N-glycans of two recombinant E2 proteins expressed in plant and mammalian cells has indicated system-specific features of glycosylation, particularly differences in mannose residue removal and in protein transport. Nevertheless, the plant-derived viral antigen induced an effective immune response in mice and the production of virus-neutralising antibodies [160].

**Table 3:** Summary of some vaccine viral antigens expressed in *Nicotiana Bentamian*a in licensed and candidate antiviral vaccines against human diseases for the period from 2019 to 2024.

Pathogen/Disease	Vaccine Antigen(s)/Type of Vaccine	Adjuvant	Vector	Vaccine Name/Developer	Status	References or Clinical Trial Identifier (NCT)	Year Reported or Regulatory Approved
**Influenza virus/Influenza**	HA protein (4 strains; displayed on the tobacco mosaic virus virions)/Subunit	None	No information available	KBP-V001/KBio Inc, Gangdong-gu Seoul, Korea	Clinical, phase 1	NCT04439695	2020
	HA (4 strains)/VLPs	None	Medicago vector	QVLP/Medicago Inc., Québec, Canada *	Clinical, phase 3 Preclinical study/mice, protectiveness	[140]	2021
	Tandem copies of conserved epitopes M2e and HA2 (fused to flagellin)/Subunit	Flagellin as a part of the fusion protein	pEff	Institute of Bioengineering, Research Center of Biotechnology of the Russian Academy of Sciences, Moscow, Russia	Preclinical study/mice, protectiveness	[161]	2020
	Tandem copies of M2e (displayed on the SAP-based nanoparticles)/Subunit	Derinat **	pEff	Institute of Bioengineering, Research Center of Biotechnology of the Russian Academy of Sciences, Moscow, Russia	Preclinical study/mice, protectiveness	[162]	2023
**Group A rotavirus/Gastroenteritis**	VP2, VP6, VP7 (genotype G1)/VLPs	Aluminum hydroxide	CPMV 160 CPMVHT	Ro-VLP vaccine/Mitsubishi Tanabe Pharma Corporation, Tokyo, Japan; Medicago Inc., Québec, QC, Canada *	Clinical, phase 1	[148]	2021
**Zika virus/Zika fever**	EDIII (fused to IgG)/Subunit	None	BeYDV	Arizona State University, Tempe, AZ, USA	Preclinical study/mice, neutralising activity of Ab	[134]	2020
	EDIII (fused to the IgG/HBcAg protein forming VLPs)/Chimeric VLPs	None	BeYDV	Arizona State University, Tempe, AZ, USA	Preclinical study/mice, neutralising activity of Ab	[135]	2020
**Dengue virus (DENV)/Dengue fever**	EDIII (fused to the HBcAg forming VLPs)/Chimeric VLPs	Aluminum-based adjuvant	pEAQ-τGFP	tHBcAg-cEDIII/University of Nottingham Malaysia, Semenyih, Malaysia; John Innes Centre, Norwich, UK	Preclinical study/mice, immunogenicity	[163]	2019
	DENV-SP, DENV-NSP(co-expression with the subsequent VLPs formation)/VLPs	None	pEAQ-HT	John Innes Centre, Norwich Research Park, Norwich, UK	Preclinical study/mice, immunogenicity	[130]	2021
**West Nile virus/Encephalitis**	EDIII (displayed on the AP205 phage VLPs; SpyTag/SpyCatcher (ST/SC) conjugation system)/Subunit	Montanide Gel	pEAQ, pTRAkc	Department of Molecular and Cell Biology, University of Cape Town, Cape Town, South Africa	Preclinical study/mice, immunogenicity	[164]	2021
**Hepatitis B(HBV)/Hepatitis B**	S/preS1 antigen/Subunit	AddaVax	pEAQ-HT-DEST1	Institute of Biochemistry of the Romanian Academy, Bucharest Romania; NIBIO—Norwegian Institute of Bioeconomy Research, Ås, Norway; “Cantacuzino” Medico-Military National Research Institute, Bucharest Romania	Preclinical study/mice, neutralising activity of Ab	[156]	2023
**Hepatitis C Virus (HCV)/Hepatitis C**	Full-size glycoprotein E2/Subunit	Aluminum hydroxide	pEAQ-HT-DEST1	Institute of Biochemistry of the Romanian Academy, Bucharest, Romania; NIBIO, Ås, Norway; Oxford Glycobiology Institute, University of Oxford, Oxford, UK, etc.	Preclinical study/mice, neutralising activity of Ab	[160]	2021
**Norovirus/Gastroenteritis**	VP1 protein (genotypes GI.4 + GII.4)/VLPs	None	magnICON^®^ technology	rNV-2v/Icon Genetics GmbH, Denka Company, Weinbergweg, D-06120 Halle, Germany	Preclinical study/rabbits, immunogenicity Clinical, phase 1	[119,120]	2022
**Immunodeficiency virus type 1 (HIV-1)/Immunodeficiency**	Env gp140/Subunit	Alhydrogel	pEAQ-HT	University of Cape Town, Cape Town, South Africa	Preclinical study/rabbits, immunogenicity	[165]	2019
	Env gp140 (co-expression with the chaperone; glyco-engineering)/Subunit	Alhydrogel	pEAQ-HT	University of Cape Town, Cape Town, South Africa; University of Southampton, Southampton UK; University of Natural Resources and Life Sciences, Vienna, Austria, etc.	Preclinical study/rabbits, neutralising activity of Ab	[166]	2022

* Medicago Inc. was dissolved in 2023 [141]; ** Derinat (sodium desoxyribonucleate) is licensed for human use in the Russian Federation as an immunomodulator.

## 5. Baculovirus-Mediated Insect Cell Expression System

Insect cells are widely used for the expression of recombinant proteins, including vaccine antigens, due to biotechnologies that allow high-yield production and post-translational modifications that are similar to those found in mammalian expression systems. The most common approach is transient expression using the baculovirus expression vector system (BEVS). Alternatively, the Drosophila S2 cell line, as a stably transformed and nonviral expression platform, can occasionally be used for the production of vaccine antigens [167,168,169]. Undoubtedly, at the moment, the baculovirus expression vector system is much more often used for this purpose, because of the ability to incorporate large fragments of target DNA and to express multiple heterologous DNAs simultaneously. BEVS is also characterised by a high level of safety (baculoviruses are insect pathogens and therefore do not replicate in mammalian cells). The most prevalent BEVS format involves the *Autographa californica* multiple nucleopolyhedrovirus (AcMNPV) and its permissive cell lines, such as Sf21, Sf9 and ExpresSF+, which originate from the pupal ovarian cells of the fall armyworm (*Spodoptera frugiperda*), or the High Five cell line derived from ovarian cells of the cabbage looper (*Trichoplusia ni*) [170]. Heterologous gene expression is usually under the control of the AcMNPV p10 promoter or the polyhedrin promoter, which is one of the strongest known in nature [171]. Various approaches have been developed to produce recombinant baculovirus, including homologous recombination (e.g., BaculoGold™ system, BD Biosciences, San Jose, CA, USA), site-specific transposition (Bac-to-Bac baculovirus expression system, ThermoFisher, Waltham, MA, USA) and combined technologies (BacMagic, Sigma-Aldrich-Novagen, St. Louis and Burlington, MA, USA and flashBAC system, Oxford Expression Technologies, Oxford, UK) [172]. The Bac-to-Bac system was a significant achievement in BEVS biotechnology, enabling the rapid creation of a baculovirus shuttle vector (bacmid) in *E. coli* cells, through site-specific transposition of the target gene, followed by the transfection of insect cells [173]. The bacmid contains a mini-F replicon for single-copy replication in *E. coli*, a kanamycin resistance gene for selection and a LacZ-mini-attTn7 transposon integration site. The Bac-to-Bac system is based on the transfer of the target gene, enclosed between the Tn7L and TnR elements of the Tn7 transposon, from the donor plasmid to the bacmid due to the presence of mini-attTn7 sites within it. This process requires the participation of the helper plasmid carrying the tns-A-E transposase gene. Bacmids have become widely used for creating expression vectors, due to their adaptability with methods developed for bacterial systems. The molecular engineering of baculovirus is constantly evolving, with ongoing optimisations to enhance protein expression. For example, new promoters, enhancers, anti-apoptotic genes, molecular chaperones and other elements are incorporated into the viral vector to increase protein yield, improve protein stability and prolong the survival of host cells during infection [174]. The use of multiple promoters makes it possible to express several proteins from a single construct.

Although proteins expressed in insect cells undergo all stages of maturation and modification inherent in eukaryotic systems, insect cells generate less complex N-glycans than mammalian cells, so this system is not always optimal. To address this limitation, great efforts have been made, in recent years, to optimise the components of viral vectors, to edit existing cell lines and generate new ones [174]. Nevertheless, for some vaccine antigens, insufficient glycosylation has been shown not to affect their antigenic properties. For example, this was demonstrated for HA and NA antigens of the influenza virus [175] and envelope glycoprotein of HIV-1 (human immunodeficiency virus 1) [176,177]. Table 4 provides an overview of some recent vaccine viral antigens expressed in BEVS, in both licensed and candidate vaccines, at various stages of preclinical and clinical trials.

### 5.1. Influenza Virus

Sanofi Pasteur’s Flublok, the first influenza vaccine based on recombinant hemagglutinin (from three different influenza strains), was licensed by the FDA in 2013, and followed by the quadrivalent Flublok (RIV4) vaccine in 2016. It is expected that RIV4 will be available in the 2023–2024 influenza season [178]. Both Flublok vaccines can be manufactured in less than two months, making them well suited for preventing pandemic influenza viruses [179]. Several studies have shown that RIV4 potentially provides better protection against influenza A infection than standard egg-based inactivated influenza vaccines [180,181,182]. This advantage is likely because the traditional method of producing inactivated influenza vaccine through serial passage in eggs can alter the antigenic properties of the vaccine strain, making it less effective against wild-type influenza strains [180]. It should be noted that RIV4 contains three times more HA (45 µg HA per strain) than the standard dose of the inactivated influenza vaccine (IIV4) (15 µg of hemagglutinin per strain).

Unfortunately, seasonal vaccines do not provide protection against novel pandemic strains and therefore require annual composition updates. Approaches to developing a universal influenza vaccine in baculovirus/insect cell expression systems include the use of conserved and consensus vaccine antigens and their combinations, as well as tandem repeats of epitopes, new delivery systems and immunostimulants. Wang and colleagues obtained double-layer protein nanoparticles by chemically cross-linking NA antigens (N1 and N2) onto the surface of M2e [183] or NP nanoparticles [184]. These vaccine candidates should be considered subunit due to the origin of nanoparticles and the nature of antigen-carrier interaction. It has been shown that immunisation with M2e-NA and NP-NA nanoparticles protects mice from homologous and heterosubtypic influenza strains and induces long-lasting immunity. The inclusion of flagellin in nanoparticles as a part of the fusion protein NA-FliC/M2e [183] or co-administration of NP-NA nanoparticles with monophosphoryl lipid A (MPLA, a toll-like receptor 4 agonist) has been shown to enhance immune response and provide improved protection against influenza [184]. The results of these studies demonstrate the potential of dual-layer protein nanoparticles as a platform for epitope-based vaccines.

Another type of epitope-based influenza vaccine candidate produced in the baculovirus system is M2e5x VLPs, which contains tandem repeats of heterologous M2e epitopes of human, avian and swine influenza A viruses [185]. Immunisation of mice with M2e5x VLPs provided robust cross-protection against viruses with various HA subtypes. Co-administration of VLPs M2e5x with a live attenuated vaccine [186], or using VLPs M2e5x as a booster, significantly improved cross-protection in mice [187]. Various approaches have been used to enhance the effectiveness of VLPs M2e5x. Gomes and colleagues used M2e5x VLPs for encapsulation in microparticles (MPs) of different types (precrosslinked polymer matrix of bovine serum albumin; poly(lactic-co-glycolic) acid (PLGA) nanoparticles, etc.) and explored different routes of administration to enhance the immunogenicity and effectiveness of the antigen, both in vitro and in vivo [188,189,190]. In the serum of mice immunised with M2e5x VLPs-MPs (transdermally or using fast-dissolving microneedles), an increased antibody titre was observed, which was further enhanced with the use of adjuvants (MPLA and Alhydrogel).

Kim and co-authors explored a novel approach for creating a VLP-based universal influenza vaccine expressed in insect cells, which included multiple consensus sequences of NA (cN1, cN2 and B-cNA) and a tandem repeat of 5xM2e, using the multigene-expressing baculovirus vector pFastBac1 [191]. Young and aged mice vaccinated with multi-subtype cNA-M2e VLPs were protected against influenza A viruses (H1N1, H5N1, H3N2, H9N2, H7N9) and influenza B viruses (Yamagata and Victoria lineages, containing significant antigenic variations) [191]. Co-administration of seasonal split vaccine with the multi-subtype cNA-M2e VLPs enhanced protection against homologous and heterologous viruses in an aged mouse model [192]. This technology provides an efficient system for developing vaccine candidates, which augments the range of tools that can be used to combat influenza virus in the face of recurrent pandemics.

### 5.2. Rotavirus

As discussed in Section 4.2, the monovalent vaccine produced in *N. benthamiana* (Ro-VLP; Table 3) by simultaneous expression of the three structural proteins, VP2, VP6 and VP7 (genotype G1), did not induce a heterotypic neutralising humoral response in infants against five heterologous genotypes of rotavirus (G2P[4], G3P[8], G4P[8], G9P[8] and G12P[8]), which are responsible for the majority of infections in most countries of the world. Using the baculovirus system, Kostina and colleagues created a polyvalent VLP-based vaccine containing VP4 and VP7 antigens of the six most relevant rotavirus genotypes in Russia (Table 4) [193]. VP4 and VP7 proteins were each cloned into a separate bacmid, rather than into a single construct. Triple-layer VLPs (2/6/7 or 2/6/4) were produced from three proteins; (for this purpose, coinfection with two baculoviruses was performed, e.g., VP2/VP6 + VP4 or VP2/VP6 + VP7). Using this technology, virus-like structures containing outer layer proteins of six different rotavirus genotypes were produced independently of each other and then combined in an equal ratio into the final composition [194]. Immunisation with the Gam-VLP-rota vaccine provided long-lasting protective humoral, secretory and cellular immunity in minipigs [193]. A double-blind, randomised, placebo-controlled study is currently underway to evaluate the immunogenicity, tolerability and safety of Gam-VLP-rota vaccine with adjuvant against rotavirus infection in healthy volunteers aged 18–45 years. The results of the study are expected soon.

### 5.3. Respiratory Syncytial Virus (RSV)

The transmembrane glycoproteins G and F (G—attachment glycoprotein; F—fusion glycoprotein) of RSV are the main targets of neutralising antibodies. The G glycoprotein is highly glycosylated and variable, except for the central conserved domain (CCD) of the protein. It is the G protein that mediates the initial attachment of the virus to the cell by binding to the CX3CR1 receptor and/or other candidate receptors such as TLR4 and HSPG [195,196]. The F glycoprotein is, however, more conserved than the G protein among all strains of groups A and B RSV and is likely to provide better cross-protection [197]; therefore, the majority of development efforts are focused on this particular protein. The trimeric F-protein on the surface of the virion exists in a metastable pre-F form and undergoes irreversible rearrangement into a nonfunctional post-F conformation during membrane fusion. The most neutralisation-sensitive antigenic sites are predominantly found in the pre-F conformation. The most common antigen for vaccine development is the stabilised pre-F; this antigen was used in vaccine formulations approved by the FDA, in 2023, for the prevention of RSV diseases (Section 6.7). Nevertheless, the development of improved vaccines that offer greater cross-protection against various RSV strains continues.

Currently, the focus is on VLP-based vaccines produced in the baculovirus expression system. Luo and colleagues used influenza virus matrix protein M1 for co-expression with the pre-F or post-F antigen, and obtained chimeric VLPs for a comparative evaluation of their immunological properties [197]. Both types of VLPs induced the production of neutralising antibodies but nAb titres were significantly higher after pre-F VLPs immunisation. Compared with post-F VLPs, immunisation with pre-F VLPs led to a more balanced Th1/Th2 immune response and reduced levels of the cytokines IL-4 and IL-5. Immunisation with pre-F VLPs protected mice from RSV without signs of immunopathology [198]. A group of Korean researchers has also developed several chimeric VLPs constructs on the platform of the M1 protein of influenza, which contain pre-F, G, Gt (G protein with tandem repeats) and pre-F+G antigens. In particular, it was demonstrated that co-immunisation with pre-F + Gt VLPs and pre-F + G VLPs significantly enhanced protection of mice against a laboratory-adapted strain [199,200] and the recombinant strain rA2-line19F, that cause severe mucus secretion and inflammation in mice, and is similar to circulating pathogenic isolates [201]. The researchers are currently conducting a comparative evaluation of the effectiveness of these VLPs in a mouse model (Table 4) [199,200,201]. In addition, Lee and colleagues reported the creation of VLPs by co-expression of pre-F and G RSV proteins with the M protein of RSV. Immunisation of mice with pre-F or G VLPs limited viral replication in the lungs and the development of pulmonary histopathology. Further studies on the effectiveness of the chimeric VLPs obtained are planned, to identify factors contributing to differences in protection, if any are observed [202].

### 5.4. Ebola Virus

Ebola hemorrhagic fever in humans is caused by four of the six known species belonging to the genus Ebolavirus: Ebola virus (EBOV, species Zaire ebolavirus), Sudan virus (SUDV, species Sudan ebolavirus), Bundibugyo virus (BDBV, species Bundibugyo ebolavirus) and Taï Forest virus (TAFV). The most severe symptoms, and the most deaths, are caused by EBOV (50–90%), SUDV (55%) and BDBV (25–50%) [203]. Although several viral vector vaccines have been approved for the prevention of Ebola virus disease [204], subunit vaccines remain a promising approach to combatting this dangerous disease; they may be effective both as a heterologous booster and as an alternative vaccine.

Most vaccines, both licensed and in development, target the surface glycoprotein (GP) of EBOV as the antigen. This glycoprotein mediates virus attachment and fusion with the host cell and serves as the major target for neutralising antibodies. The baculovirus expression platform has been used to express the full-length GP EBOV (Guinea 2014 variant). The recombinant GP was produced in Sf9 insect cells as glycosylated trimers and formed spherical particles 30–40 nm in size upon purification [205]. Despite the formation of these particles, the mismatch between their structure and the structure of Ebola virus’ virion and the fact that GP is a surface glycoprotein and not a capsid protein allow classify this vaccine as a subunit vaccine. Immunisation of mice with EBOV GP in combination with the Matrix-M adjuvant fully protected animals from a lethal dose of the virus. A phase I clinical trial demonstrated safety and relatively high levels of neutralising antibodies compared with the FDA-approved rVSV-ZEBOV vaccine. Antibody titres remained elevated for 385 days [206].

Vaccines against Sudan ebolavirus are also being developed. Wu and colleagues designed and produced a VLP-based vaccine against SUDV in insect cell line through the co-expression of two viral structural proteins, GP and the matrix protein VP40 [207]. For that purpose, *GP* and *VP40* genes were cloned into the pFastBacDual vector (pFastBacDual-VP40-VP40 and pFastBacDual-GP-GP), using dual promoters (p10 and polyhedron) to increase yields. The vaccine induced antigen-specific IgG and neutralising antibodies in mice and horses.

**Table 4:** Summary of some vaccine viral antigens expressed in insect cells in licensed and candidate antiviral vaccines against human diseases for the period from 2019 to 2024.

Pathogen/Disease	Vaccine Antigen(s)/Type of Vaccine	Adjuvant	Insect Cells Lines	Vaccine Name/Developer	Status	References or Clinical Trial Identifier (NCT)	Year Reported or Regulatory Approved
**Influenza virus/Influenza**	HA (4 strains; displayed on the Matrix-M1 nanoparticles)/Subunit	Matrix-M1	Sf9	Quad-NIV/Novavax, Gaithersburg, MD, USA	Clinical, phase 3	[208]	2022
	M2e-N1 and M2e-N2 nanoparticles/Subunit	None	No information	Georgia State University Institute for Biomedical Sciences, Atlanta, USA	Preclinical study/mice, cross-protectiveness	[209]	2020
	NA1-FliC-M2e and NA2-FliC-M2e nanoparticles/Subunit	Flagellin (FliC) as a part of the fusion protein	Sf9	Georgia State University Institute for Biomedical Sciences, Atlanta, USA	Preclinical study/mice, cross-protectiveness	[183]	2022
	NP-N1 and NP-N2 nanoparticles/Subunit	MPLA	Sf9	Georgia State University Institute for Biomedical Sciences, Atlanta, USA; Texas Tech University, Lubbock, USA	Preclinical study/mice, cross-protectiveness	[184]	2023
	Consensus multi-NA (subtypes cN1, cN2, B cNA) and M2e/VLPs	None	Sf9	Georgia State University, Atlanta, Georgia, USA; Medigen, Inc., Frederick, MD, USA	Preclinical study/mice, cross-protectiveness	[191]	2022
	Tandem repeats of two human M2e, swine M2e, avian M2e (type I and II)/VLPs	ASO4 (MPLA^®^ + Alhydrogel^®^)	Sf9	Mercer University, Atlanta, GA, USA; Georgia State University, Atlanta, GA, USA	Preclinical study/mice, immunogenicity	[188]	2021
	M2e (SpyTag/SpyCatcher bioconjugation or fusion with the norovirus VP1 capsid protein forming VLPs)/Subunit or Chimeric VLPs	Aluminum hydroxide	High Five™	Mercer University, Atlanta, GA, USA Tampere University, Tampere, Finland	Preclinical study/mice, immunogenicity	[210]	2023
	H1/H1 stem (fused to the M1 influenza protein forming VLPs)/Chimeric VLPs	DnaK as a part of cVLPs; Freund’s adjuvant	Sf9	Hunan University, Changsha, China; Georgia State University, Atlanta, GA, USA; Beijing Weimiao Biotechnology Co., Ltd., Beijing, China, etc.	Preclinical study/mice, protectiveness	[211]	2022
**Human papillomavirus (HPV)/Cervical cancer**	L1 protein (14-valent; types 6/11/16/18/31/33/35/39/45/51/52/56/58/59)/VLPs	No information	No information	SCT1000/Sinocelltech Ltd., China	Clinical, phase 3	NCT06041061	2023
	Modified L1 protein (types 6, 11, and 52)/VLPs	Aluminium hydroxide, MPLA	Sf9	Institute of Basic Medical Sciences Chinese Academy of Medical Sciences, School of Basic Medicine Pecking Union Medical College, Beijing, China	Preclinical study/mice, immunogenicity, neutralising activity of Ab	[212]	2023
	Modified L1-L2 protein (16L1-33L2, 58L1-16L2) (conserved cross-neutralising epitopes of the minor capsid protein L2 displayed on the L1 VLPs)/Chimeric VLPs	Aluminium hydroxide, MPLA	Sf9	Institute of Basic Medical Sciences Chinese Academy of Medical Sciences, School of Basic Medicine Pecking Union Medical College, Beijing, China	Preclinical study/mice, immunogenicity, neutralising activity of Ab	[212]	2023
**Group A rotavirus/Gastroenteritis**	VP4(type P4), VP4(type P8), VP7(type G1), VP7(type G2), VP7(type G4) VP7(type G9) (mix of 6 VLPs)/VLPs	None	Sf9	Gam-VLP-rota/National Research Center for Epidemiology and Microbiology named after Honorary Academician N.F. Gamaleya of the Ministry of Health of the Russian Federation, Moscow, Russia; RMC «HOME OF PHARMACY» JSC, St. Petersburg, Russia	Preclinical study/minipigs, immunogenicity	[193,194]	2021; 2023
**Zika virus/Zika fever**	Baculovirus displaying ZIKV E protein/Subunit	Freund’s adjuvant	Sf9	Chinese Academy of Sciences, Wuhan, China	Preclinical study/mice, protectiveness	[213]	2020
**Respiratory syncytial virus (RSV)/RSV infection**	Prefusion F (Pre-F) and G with tandem repeats (Gt) RSV antigens (co-expression with the influenza protein M1 forming VLPs)/Subunit	None	Sf9	Kyung Hee University, Seoul, Republic of Korea	Preclinical study/mice, protectiveness	[199,201]	2022; 2024
	Pre-F and G RSV proteins (co-expression with the influenza protein M1 forming VLPs)/Subunit	None	Sf9	Kyung Hee University, Seoul, Republic of Korea	Preclinical study/mice, protectiveness	[200]	2023
	Pre-F and G RSV antigens (co-expression with the RSV M protein forming VLPs)/Subunit	None	Sf9	Kyung Hee University, Seoul, Republic of Korea	Preclinical study/mice, protectiveness	[202]	2023
	Pre-F and Post-F(co-expression with the influenza protein M1 forming VLPs)/Subunit	None	Sf9	Wuhan University, Wuhan, China	Preclinical study/mice, protectiveness	[198]	2022
**Ebola virus (EBOV)/Hemorrhagic fever**	EBOV glycoprotein/Subunit	Matrix-M	Sf9	Novavax, Inc., Gaithersburg, MD, USA; Philipps University of Marburg, Germany; German Center for Infection Research (DZIF), Partner Site Gießen-Marburg-Langen, Marburg, Germany, etc.	Clinical, phase 1	[206]	2020
	Sudan virus matrix structural protein VP40 and glycoprotein GP (co-expression with the subsequent VLPs formation)/VLPs	Montanide ISA 201 (mice) or Freund’s adjuvant (horse)	Sf9	Institute of Military Veterinary Medicine, Academy of Military Medical Sciences, Changchun, China; College of Wildlife Resources, Northeast Forestry University, Harbin, China, etc.	Preclinical study/mice and horses, immunogenicity, neutralising activity of Ab	[207]	2020

## 6. Mammalian Expression Systems

The production of recombinant proteins in mammalian cells has significant advantages over the bacterial-, yeast-, insect- or plant-based expression systems currently used for this purpose. Despite the higher cost of the final products, the mammalian expression system is valued for its ability to express complex proteins of eukaryotic viruses in a native conformation with the necessary post-translational modifications and correct subunit assembly. In addition to high cost, this expression system has a number of other disadvantages, such as low production rate, low protein yield and the risk of viral contamination [214]. Currently, several types of cell lines are used to produce recombinant vaccine antigens, with the most popular being various lines of Chinese hamster ovary (CHO) cells and human embryonic kidney 293 (HEK 293) cells (Table 5). HEK 293 cell lines are well suited for transient expression of recombinant proteins, due to the ease of transfection with plasmid DNA. Transduction using baculovirus (BacMam) can be used for the expression of multicomponent protein complexes [215]. CHO cells are more commonly used to generate lines that stably express the target protein. Such lines can be obtained by transfection with a plasmid vector or, for example, by transduction with a lentivirus-based vector [216] with random integration into the genome and subsequent selection of clones. Alternatively, site-specific methods of integrating the target gene into specific loci can be used [217]. Despite the advantages of stable CHO lines, it should be noted that glycosylation patterns may differ between these cells and HEK 293 cells. HEK 293 cells are a human cell-derived line, meaning that their glycosylation patterns could be more suitable for human viruses’ protein production. Therefore, if a specific glycosylation is required for the correct functioning of a recombinant protein, CHO cells may not always provide the optimal system for its expression. To solve this problem, improved glycoengineered CHO strains are being developed to produce vaccine antigens, using various strategies [218,219,220]. Another problem with the expression of recombinant proteins in CHO cells is protein degradation, which can have various causes, including the degree of glycosylation. Protein degradation affects the quality of the final substance [221]. For example, degradation of the gp120 monomeric protein (HIV1 vaccine antigen) occurs from proteolysis by the C1s endogenous serine protease in the V3 loop region, which is important for recognition by broadly neutralising antibodies (bnAbs) [222]. To counter serine protease-induced protein proteolysis, a new C1s−/− CHOK1 cell line was generated, using CRISPR/Cas9 technology to knockout the *C1s* gene [223]. Currently, of all mammalian cell-based expression systems, CHO lines remain the preferred option for the production of recombinant proteins in the biopharmaceutical industry [224,225].

Table 5 provides an overview of the most recent prophylactic vaccine virus antigens that have been successfully expressed in mammalian cells and then used in both licensed vaccines and candidate vaccines at various stages of preclinical and clinical trials.

### 6.1. Influenza Virus

To develop a broad-spectrum vaccine against avian influenza virus (H5N1), Chen and colleagues obtained CHO cell clones stably expressing the recombinant vaccine antigen rH5HA, with mutations at positions 127 (127NSS129) and 138 (138NGT140) in the variable region of the HA head, introducing two new N-glycosylation sites [226]. In earlier works of the same group [227,228], it was shown that such additional glycosylation leads to a refocusing of the immune response to the conserved stem domain, which is more favourable in the context of wide-spectrum vaccine design. The HA double mutant (g127 + g138) induced potent titres of neutralising antibodies that were effective against heterologous H5N1 viral strains. A number of antibodies specific to the stem part of HA correlated with the number of protective antibodies. These HA stem-specific antibodies provided protection against live viral challenges [226].

### 6.2. Flaviviruses

As with other antiviral vaccines, the development of vaccines against flaviviruses encounters a number of problems and complications, which have been discussed in Section 2.4 and Section 3.2.

Thoresen and colleagues developed a quadrivalent VLP-based vaccine candidate against four dengue virus serotypes [229]. This vaccine candidate comprises the prM and the E protein, co-expressed in mammalian cells. The F108A mutation was introduced into the structure of the fusion loop of E protein domain II, which increased VLPs production. In preclinical studies, this vaccine was shown to induce sustained neutralising antibody responses (up to 1 year) against all four Dengue virus (DENV) serotypes in nonhuman primates, and to reduce viral replication upon subsequent challenge. The transfer of purified IgGs from immunised monkeys to immunodeficient mice protected them from subsequent lethal DENV challenge. In addition, vaccination with tetravalent VLPs did not result in ADE for DENV serotypes 1–4, as demonstrated in vitro [229].

Several vaccines based on VLPs have also been developed against the Zika virus. De Lorenzo and colleagues constructed a plasmid encoding the capsid anchor region, followed by full-length prM and E [230]. Expi293F cells were transfected with this plasmid. A disulfide bridge was introduced into the E protein sequence by substitution A264C, which ensured the stabilisation of the E protein in the conformation of a covalent dimer. Such a structure should reduce exposure to undesirable cross-reactive epitopes responsible for ADE and enhance exposure to epitopes for the most potent neutralising antibodies. Indeed, the redesigned VLPs induced antibodies that, in in vitro experiments, reduced the effect of ADE of DENV, yellow fever virus (YFV) and West Nile virus (WNV) infections, in comparison with a similar vaccine candidate corresponding to the wild-type. Vaccinated mice produced antibodies with a high level of neutralisation activity, enabling them to successfully cope with the lethal challenge of both Asian (PRVABC59) and African (MP1751) lineages of ZIKV [230]. Immunisation prevented acute disease after subjects were bitten by *Aedes aegypti* mosquitoes infected with ZIKV. Moreover, transmission of the virus from the host to the vector was shown to be inhibited by vaccination [231].

Vang and co-authors developed another VLP-based vaccine candidate against ZIKV [232]. HEK293 cells expressed a cassette plasmid containing chimeric genes of structural proteins of two different ZIKV strains: the prM (African MR766 strain, 168 aa), E ectodomain (Brazilian SPH2015 strain, 405 aa) and the E stem-anchor (African MR766 strain, 99 aa). Resulting VLPs have been shown to induce high nAb titres in mice and rhesus macaques, the levels of which correlate with the level of protection against ZIKV infection.

Two different subunit vaccines involving Zika virus E protein domain III (EDIII) have been developed using an approach of the target antigen fusion with the Fc-fragment of human IgG1. Tai and colleagues identified in EDIII an immunodominant non-neutralising epitope that is hidden within full-size E protein, but exposed in the recombinant domain [233]. They introduced an additional N-glycosylation site, to refocus the immune system on neutralising epitopes. A modified antigen contained two mutations (M375N and E377T) in EDIII and was fused with the Fc-tag of human IgG1. The level of protection was studied in both immunocompetent and immunocompromised mice, which are considered to be a lethal model for ZIKV. In comparison with the wild-type EDIII-based antigen, the modified antigen was shown to induce greater protective efficacy in immunocompromised mice. In adult mice, as well as their unborn offspring, vaccination with the designed antigen significantly reduced viral titres and provided protection [233].

Su and co-authors also used Fc fragment of IgG1 to enhance the immunogenicity of the EDIII ZIKV antigen as a part of a subunit vaccine and its yield during expression in mammalian cells [234]. Using a lentiviral expression system, EDIII-Fc was obtained in 293T cells, which stably secreted the recombinant protein. It was shown that EDIII-Fc significantly inhibited viral replication in a mouse model and induced the production of neutralising antibodies and T-cell immune response against Zika virus in immunised rhesus macaques.

### 6.3. Varicella Zoster Virus (VZV)

Currently, there are two FDA-approved vaccines against herpes zoster—live attenuated vaccine, ZVL, and a recombinant vaccine, Shingrix (GSK, UK), based on the shortened form of gE VZV, as mentioned previously (Section 2.5). The Shingrix vaccine contains an AS01B liposome-based adjuvant system that includes the immunostimulants MPLA and Quillaja saponaria Molina, fraction 21 (QS21) [235]. One of the key components of the AS01B adjuvant system is a mixture of polysaccharides extracted from the bark of *Quillaja saponaria*, which is native to South America. The high cost, the impossibility of chemical synthesis and the limited supply of QS21 have led to the development of new vaccine candidates based on a truncated form of VZV gE in combination with various adjuvants. In preclinical studies, the immunogenicity of PLGA [236] or lipid [237] nanoparticles containing VZV gE, together with the immunostimulants CpG ODN and Poly I:C, was compared with gE adjuvanted with aluminum hydroxide. Both nanoparticle formulations demonstrated better efficiency [236,237]. In another study, in a mouse model, lipid nanoparticles containing VZV gE and CpG ODN demonstrated an immunogenicity similar to that of the Shingrix vaccine [238]. It can be concluded that these nanoparticle-based vaccine candidates are quite promising, given their good immunogenicity profile and the fact that all of their components can be artificially synthesised in high quantities, which significantly reduces production costs.

Korean researchers are currently developing a subunit vaccine candidate against HZ that is based on a truncated form of gE in combination with a new liposomal adjuvant system, CIA09A. CHO cells were transfected with a plasmid encoding a truncated *gE* gene lacking the hydrophobic anchor and carboxy-terminal domains. The plasmid also contained a CMV promoter and the mouse *DHFR* gene for clone selection. The composition of the medium and cell culture conditions were optimised to ensure sustainable growth of the culture and efficient production of the recombinant antigen; (the protein yield was 2440 mg per litre of culture). In immunised mice, the vaccine candidate induced both humoral and T-cell immune responses [239,240].

### 6.4. Hepatitis B Virus

In 2021, the FDA approved the PreHevbrio vaccine, which was previously licensed in Israel as Sci-B-Vac™, against all known subtypes of hepatitis B virus (HBV) for individuals over 18 years of age (Table 5). This vaccine contains VLPs that include three HBV envelope antigens: small surface protein (S), medium protein (Pre-S2) and large protein (Pre-S1). Antigens were produced in CHO cells. The availability of Pre-S2 and Pre-S1 antigens in the purified VLP vaccine preparation was confirmed in laboratory animals that produced the corresponding antibodies after immunisation [241]. Clinical studies have confirmed the safety of the vaccine, which also showed better immunogenicity than yeast vaccines against HBV in special patient groups (immunocompromised patients, patients with renal failure, etc.). Booster vaccination provided an immune response in individuals who had no, or poor, response to previous immunisation with yeast HBV vaccine [241,242]. When using a vaccine comprising three antigens, seroprotection forms more quickly than with a single-antigen vaccine, contributing to fewer infections, complications and deaths from HBV [243].

### 6.5. Hepatitis C Virus

Currently, the attention of researchers concerned with the design of antigens for vaccinating against Hepatitis C Virus (HCV) is focused on approaches based on the use of glycoproteins E2 and E1/E2, along with various delivery systems. Problems with the use of E1 and E2 complex transmembrane proteins as a base for vaccine development have been described in detail in Section 4.4.

Pierce and colleagues presented a panel of E2 glycoprotein constructs [244]. The antigen design was focused on the antigenic D domain, which is targeted by bnAbs, and the antigenic A domain, which is targeted by non-neutralising antibodies. In order to stabilise D domain conformation, a point mutation was introduced into a key conserved epitope. A new N-glycosylation site masking the A domain site was added, to refocus immune response and reduce the production of non-neutralising antibodies targetting this region.

He and co-authors applied a complex approach to the development of a vaccine against HCV, using structure-based optimisation of E2 core to obtain vaccine constructs that carried only conserved epitopes of bnAbs [245]. Self-assembling protein nanoparticles were used to display conserved epitopes of bnAbs within a subunit vaccine. Vaccine constructs showed good immunogenicity in experiments on laboratory animals.

McGregor and colleagues created chimeric VLPs based on the duck HBV small surface antigen (S) as a scaffold carrying an optimised E2 glycoprotein ectodomain that lacks three variable regions [246]. This modified form of E2 glycoprotein ectodomain had been shown to elicit bnAbs omore effectively than the nonmodified one.

All of the E2 protein-based antigens described above, in the presence of various adjuvants, elicited antibodies that demonstrated the cross-neutralisation of heterologous viruses (Table 5).

Most recombinant protein vaccines against HCV usually include only E2 and produce a weak neutralising antibody response. Recently, promising results have been achieved through the creation of stabilised and secreted E1/E2 [247] and E2/E1 constructs, which were obtained by rearranging the genes coding the E1 and E2 subunits [248]. In earlier studies, E1/E2-based antigens did not demonstrate significant superiority over E2 antigens in inducing bnAbs [249]. The complex interaction between the E1 and E2 subunits causes difficulties with obtaining this antigen, but efforts to study the structure of E1/E2 and to create new modifications of this glycoprotein complex, facilitating its successful expression in mammalian cells, may lead to significant advances in its application.

### 6.6. Human Immunodeficiency Virus Type 1 (HIV-1)

There is currently no vaccine against Human immunodeficiency virus type 1 (HIV-1). Despite research and development that has been ongoing for more than 40 years, the challenges for creating an effective vaccine remain. It is generally accepted that the development of this vaccine is one of the most difficult tasks in vaccinology. The HIV-1 envelope glycoprotein (Env) is the primary target of neutralising antibodies. Functional Env is a trimer of heterodimers consisting of gp120 and gp41, which are formed by proteolytic cleavage of the precursor gp160. Env binds to the CD4 cell receptor through one of two noncovalently associated subunits (gp120). The resulting conformational changes activate another subunit (gp41), which leads to the fusion of the viral membrane with the cell membrane. Env is highly glycosylated, which is responsible for the relatively high molecular weight of the monomer (about 160 kDa); while about half of its mass is carbohydrates [250].

Currently, the main efforts of researchers are aimed at developing vaccine antigens capable of inducing broadly neutralising antibodies (bnAbs). Here there are some examples of such antigens developed for subunit HIV-1 vaccines. Current antigen design strategies for creating an effective HIV-1 vaccine have been reviewed by Haynes and colleagues [250]. The main bnAb epitopes are presented only in the prefusion conformation of Env, so rational antigen design focuses on obtaining this form [251].

Kwon and colleagues obtained an antigen that mimics the native HIV-1 Env trimer in a stabilised closed prefusion conformation [252]. The antigen was constructed from clade A strain BG505 and named trimer 4571. The SOSIP construct (BG 505 SOSIP.664) was used as a base for trimer 4571. In order to stabilise the prefusion conformation of trimer SOSIP, an engineered disulfide bond between gp120 and gp41 was added, as well as a mutation in the heptad repeat 1 region (I559P). Although this trimer contained stabilising mutations, it was still recognised by non-neutralising (nontargetting), CD4-induced antibodies [252]. Therefore, an additional mutation that leads to the formation of one more disulfide bond between 201 and 433 cysteine residues in gp120 was added to trimer 4571, to prevent any CD4-induced conformational changes. A phase I clinical trial of trimer 4571 demonstrated its safety and immunogenicity. Nevertheless, titres of neutralising antibodies were low and were detected only in the group of individuals who received the maximum dose of antigen (500 μg) [253]. Difficulties encountered in obtaining protective bnAbs may be explained by the different immune evasion strategies of HIV-1, including genetic diversity due to the high mutation rate of Env (1–10 mutations per genome per replication cycle) and HIV recombination, as well as the dense glycan shield on Env, which hides critical antigenic epitopes from the immune system [254,255,256].

Zhang and co-authors prepared an uncleaved prefusion-optimised (UFO) BG505 trimer with modified glycans [257]. To increase immunogenicity and stability, the trimer was presented on the surface of single-component, self-assembling protein nanoparticles. The antigen induced neutralising antibody and germinal center reactions, and increased the frequency of vaccine responses in various animal models.

Env trimers corresponding to the clade C HIV-1 were stabilised in a prefusion conformation and displayed on liposomes comprising cobalt-porphyrin phospholipid (CoPoP). In preclinical studies, rabbits were sequentially immunised with three different variants of Env-CoPoP particles containing consensus and mosaic sequences. The particles induced neutralising antibodies that were cross-active against 18 of 20 multiclade tier 2 HIV-1 strains [258].

Another promising strategy is heterologous vaccination regimens that prime germline precursor bnAbs with immunogen in order to induce HIV-specific bnAbs. Nanoparticle HIV vaccine-priming candidate eOD-GT8, produced in HEK293 cells [259], when combined with adjuvant AS01B, demonstrated a favourable safety profile in phase I clinical trials. It successfully induced VRC01 class bnAb precursors in 97% of vaccine recipients and publicly available T helper cell responses [260,261]. Additional immunogen-based boosters with more native-like stabilised Env trimers are needed to trigger the further maturation and production of potent bnAbs.

### 6.7. Respiratory Syncytial Virus (RSV)

Problems associated with vaccine development were discussed in Section 2.7 and Section 5.3. On 3 May 2023, the FDA approved the world’s first RSV vaccine, developed by GSK (Arexvy), and on 31 May 2023, Pfizer’s bivalent vaccine (Abrysvo) received approval. Both vaccines were approved for use by adults aged over 60. A recombinant stabilised F protein in a prefusion conformation (pre-F), which was obtained in genetically modified stable CHO cells, was applied as an antigen in both vaccines.

Another subunit vaccine candidate, DS-Cav1, is a prefusion-stabilised trimer of the F protein ectodomain of RSV (subtype A2) that was produced in CHO cells. The results of phase I clinical trials of the DS-Cav1 have been published, reporting that a single vaccination with DS-Cav1 without adjuvant resulted in a significant increase in the neutralising activity of serum antibodies against RSV subtypes A and B. Antibodies persisted in vaccinated people for more than one RSV season [262].

Stabilised forms of pre-F may differ in the sequences of the F protein of different RSV strains, and also in the genetic modifications that provide trimerisation and stabilisation of the protein in the prefusion conformation [263]. Pang and colleagues presented a systematic review and meta-analysis of 22 studies on different types of vaccines containing RSV pre-F (subunit, adenoviral vector vaccines and mRNA vaccines). Subunit vaccines were revealed to be effective in preventing RSV but, just as importantly, they were significantly safer than other vaccine candidates [264]. However, vaccines for children, who are at the highest risk, are not yet available.

Other approaches currently used to develop vaccine antigens in mammalian expression system (2019–2024) are based on G protein as the target antigen (subunit vaccine) and obtaining chimeric VLPs of various formulations [265,266]. In recent research by McGinnes and colleagues, Newcastle disease virus (NDV) M and NP core proteins were used to create VLPs, incorporating chimeric NDV HN and F proteins fused with RSV antigens [265]. The transmembrane and cytoplasmic domains (TM/CT) of NDV F or HN proteins were fused with DS Cav1 (the RSV pre-F ectodomain) or the RSV G ectodomain, respectively. This design enabled successful assembly into chimeric VLPs. Three types of VLPs were obtained, namely those containing only DS Cav1, or only the G protein, or both RSV proteins. G protein within VLPs has been shown to provide additional stabilisation of the pre-F. VLPs simultaneously containing both pre-F and G proteins were shown to elicit significantly higher titres of neutralising antibodies than those comprising only one of the proteins. In studies carried out in a cotton rat model, these VLPs demonstrated improved protection [265]. These results correlate with data obtained from a study of VLPs assembled from the M1 protein of the influenza virus, which included recombinant RSV proteins expressed in insect cells (Section 5.3; Table 4), and indicate the promise of this approach. In a study by Voorzaat and colleagues, the highly conserved central domain of the G protein was used as an antigen, which was displayed on the surface of self-assembled lumazine synthase nanoparticles from the thermophile *Aquifex aeolicus*. Immunisation of mice with these nanoparticles led to the induction of neutralising antibodies, whose activity was confirmed in neutralisation assays on primary, fully differentiated cultures of human airway epithelial cells [266].

### 6.8. Ebola Virus

Xu and colleagues are developing a vaccine antigen that can simultaneously protect against three major species of Ebola virus, which pose the greatest danger: EBOV, SUDV and BDBV [267]. The development is carried out with a view of a subunit vaccine creation. The opportunity of developing such broad coverage vaccine is theoretically confirmed by the fact that monoclonal antibodies, which can simultaneously neutralise all three types of virus, have been isolated. It is known that, upon entry into the cell, EBOV glycoprotein (GP) undergoes proteolytic processing in the endosome, and the mucin-like domain and glycan cap region are cleaved, exposing a conserved receptor-binding domain that is necessary for binding to the intracellular receptor. A panel of antigens with additional glycosylation sites, introduced to mask the highly variable region of the glycan cap, has been prepared. This approach makes it possible to redirect the antibody response to conserved and neutralising epitopes. *Helicobacter pylori* ferritin nanoparticles containing wild-type GP have been shown to induce neutralising antibodies only against EBOV in immunised mice. Hyperglycosylated GP variants obtained by site-directed mutagenesis, and presented on ferritin nanoparticles, have induced the formation of neutralising antibodies against EBOV and two other virus species, SUDV and BDBV [267].

### 6.9. Kaposi Sarcoma Herpesvirus (KSHV or HHV8)

Kaposi’s sarcoma-associated herpes virus (KSHV), also known as human herpesvirus type 8 (HHV8), is found everywhere, but most often in sub-Saharan Africa and the Mediterranean region [268]. Vaccination of high-risk groups and children in populations with high viral prevalence could stop transmission of the virus. There is, however, no vaccine against KSHV and research and development funding are limited [269]. Mulama and colleagues generated chimeric multivalent KSHV VLPs containing ectodomains of four KSHV glycoproteins (gpK8.1, gB and gH/gL) required for viral entry into various cell types [270]. KSHV glycoproteins were fused with the TM/CT domains of the NDV F protein and VLPs were produced in CHO cells. Stable CHO cells expressing chimeric proteins were co-transfected with plasmids encoding the NDV M and NP proteins. KSHV glycoproteins (under a single CMV promoter) were interspersed with the picornavirus 2A self-cleaving peptide, for efficient expression. Purified immunoglobulins from rabbits immunised with these VLPs neutralised KSHV in epithelial, endothelial, fibroblast and B cell lines (60–90% at the highest concentration tested). IgG levels specific to all four KSHV glycoproteins were, however, short-lived and decreased significantly 70 days after the second immunisation [270].

### 6.10. Epstein-Barr Virus (EBV or HHV-4)

Epstein-Barr virus (EBV, HHV-4) is a ubiquitous herpesvirus, by which more than 90% of the world’s adult population is infected. It is the leading cause of infectious mononucleosis and a major risk factor for the further development of several diseases, including multiple sclerosis and various types of cancer [271,272]. During primary infection, EBV is typically transmitted through saliva and infects resting B cells or epithelial cells in the oropharynx and then triggers transcriptional programming of the B cells to establish lifelong viral latency [273]. There is no vaccine against EBV. The main targets of neutralising antibodies are the EBV surface glycoproteins gp350, gH, gL, gB and gp42, which mediate virus entry into the cell. Early prophylactic EBV vaccines based on a single gp350 antigen had limited clinical efficacy [274].

Antibodies to the gH/gL glycoprotein complex of EBV were revealed to be the key components of human plasma that neutralise infection in epithelial cells. At the same time, antibodies to gH/gL and gp42 were shown to be responsible for viral neutralisation in B cells. The study by Bu and co-authors addressed the generation of gH/gL-ferritin and gH/gL/gp42-ferritin nanoparticles (NP) using Expi293F cells [275]. Cells were co-transfected with plasmids encoding individual proteins. Immunisation of mice and nonhuman primates with the subunit vaccine containing gH/gL-ferritin or gH/gL/gp42-ferritin nanoparticles elicited antibodies that effectively neutralised viral infection in both epithelial cells and B cells. Serum collected from nonhuman primates inhibited EBV fusion with epithelial cells and B cells. Cell type-independent protection against EBV infection was also demonstrated. Subsequently, the same group of researchers improved the design of vaccine candidates. For that, genetic constructs ensuring the expression of gH/gL and gH/gL/gp42 as a single polypeptide were created. This approach enabled both a reduction in the number of components and product homogeneity, which is quite important in the development of pharmacological substances. Immunisation of mice, ferrets and nonhuman primates with a single-chain gH/gL-NP or a single-chain gH/gL/gp42-NP generated high titres of antibodies that were able to neutralise EBV entry into B cells and epithelial cells. When this vaccine was mixed with another EBV vaccine candidate, representing nanoparticles displaying the gp350 protein, no immune competition was observed, so it is likely that a bivalent vaccine formulation containing both gp350 and gH/gL/gp42 components will provide improved coverage against the virus on different cell types [276].

Escalante and colleagues developed a pentavalent chimeric vaccine candidate based on NDV VLPs comprising five EBV glycoproteins, (gp350, gB, gp42, gH and gL) [277]. A polycistronic construct gp350-F-gB-F-gp42-gL-gH-F, interspersed with unique 2A autocleavable linker sequences, was obtained. In addition, the transmembrane domains of gp350, gB and gH were replaced with the TM/CT domains of the NDV F protein, which ensured the incorporation of the designed glycoprotein into the NDV VLPs. For stable expression and production of VLPs in the CHO cell line, co-transfection of this plasmid and constructs encoding NDV structural proteins was carried out. Immunisation of New Zealand white rabbits with chimeric VLPs induced the synthesis of antibodies capable of neutralising viral infection in in vitro experiments on both B cells and epithelial cells, with greater efficiency than antibodies induced after administration of the soluble gp350 ectodomain [277].

Malhi and co-authors, using computational technology, designed nanoparticles presenting on their surface 60 copies of gH/gL [278]. The passive transfer of purified IgG isolated from the blood of animals immunised with gH/gL nanoparticles provided protectivity against a high-dose EBV lethal challenge in a humanised mouse model. These results support the effectiveness of applying gH/gL nanoparticles for EBV vaccine development.

**Table 5:** Summary of some vaccine viral antigens expressed in mammalian cells in licensed and candidate antiviral vaccines against human diseases for the period from 2019 to 2024.

Pathogen/Disease	Vaccine Antigen(s)/Type of Vaccine	Adjuvant	Cell Line	Vaccine Name/Developer	Status	References or Clinical Trial Identifier (NCT)	Year Reported or Regulatory Approved
**Influenza virus/Influenza**	Modified H5HA/Subunit	PELC/CpG	CHO	National Tsing Hua University, Taiwan; University of Manitoba, Manitoba, Canada	Preclinical study/mice, cross-clade neutralising Ab, cross-protectiveness	[228]	2019
**Zika virus/Zika fever**	Modified EDIII (fused to the IgG1-Fc fragment)/Subunit	Aluminum hydroxide +MPLA	293T	Lindsley F. Kimball Research Institute, New York, NY, USA; Beijing Institute of Microbiology and Epidemiology, Beijing, China	Preclinical study/immunocompromised mice and their fetuses, protectiveness	[233]	2019
	E protein dimer/VLPs	Aluminum hydroxide +MPLA	Expi293F	MRC-University of Glasgow Centre for Virus Research, Scotland, UK; International Centre for Genetic Engineering and Biotechnology, Trieste, Italy; Navamindradhiraj University, Bangkok, Thailand, etc.	Preclinical study/ mice, protectiveness	[230]	2020
	E protein dimer/VLPs	AddaVax	Expi293F	MRC-University of Glasgow Centre for Virus Research, Scotland, UK; Research and Development Institute, Government Pharmaceutical Organization, Bangkok, Thailand; Navamindradhiraj University, Bangkok, Thailand, etc.	Preclinical study/ a mosquito transmission mouse model, protectiveness	[231]	2023
	prM-E/VLPs	Alhydrogel	HEK293	Emergent BioSolutions Inc., Gaithersburg, MD, USA, etc.	Preclinical study/ mice, nonhuman primates, protectiveness	[232]	2021
	EDIII (fused to the human IgG1-Fc fragment)/Subunit	Freund’s complete adjuvant or MF59	293T	Southern Medical University, Guangzhou, China; Sun Yatsen University Cancer Center, Guangzhou, China; South China Institute of Large Animal Models for Biomedicine, Wuyi University, Jiangmen, China, etc.	Preclinical study/ mice, rhesus macaques, protectiveness	[234]	2023
**Dengue virus (DENV)/Dengue fever**	Modified prM-E (1–4 serotypes)/VLPs	Aluminum hydroxide	FreeStyle 293F	DENVLP/VLP Therapeutics, USA Nagasaki University, Japan	Preclinical study/non-human primates, protectiveness	[229]	2024
**Varicella zoster virus (VZV)/Varicella, herpes zoster**	Glycoprotein E (truncated)/Subunit	CIA09A	CHO DG44	Sejong University, Seoul, Republic of Korea; R & D Center, EyeGene, Goyang, Republic of Korea; Korea Polytechnic University, Seongnam, Republic of Korea	Preclinical study/mice, immunogenicity	[239]	2019
**Hepatitis B virus (HBV)/Hepatitis B**	Triple antigen S, preS1, and preS2/VLPs	Aluminium hydroxide	CHO	PreHevbrio/(Sci-B-Vac /Bio-Hep-B™/Hepimmune™)/VBI Vaccines, Cambridge, MA, USA	Licenced	[279]	2021
**Hepatitis C Virus (HCV)/Hepatitis C**	Modified E2 (residues 384–661)/Subunit	Polyphosphazene	FreeStyle HEK293-F	University of Maryland, USA; Princeton University, Princeton, USA; Stanford University School of Medicine, Stanford, USA, etc.	Preclinical study/mice, cross-neutralising activity	[244]	2020
	Modified core E2 (genotype 1a or 6a; displayed on the ferritin or E2p or I3-01 nanoparticles)/Subunit	AddaVax	HEK293-F	The Scripps Research Institute, La Jolla, CA, USA	Preclinical study/mice, cross-neutralising activity	[245]	2020
	Modified E2E1 (6 strains; displayed on the I53-50A nanoparticles)/Subunit	Squalene emulsion	Freestyle 293-F	Amsterdam UMC, The Netherlands; Amsterdam Institute for Infection and Immunity, Amsterdam, The Netherlands; University of Southampton, Southampton, UK, etc.	Preclinical study/rabbits, cross-neutralising activity	[248]	2022
	Modified E1E2/Subunit	Polyphosphazene	Expi293	University of Maryland, USA; Princeton University, Princeton, USA; Stanford University School of Medicine, Stanford, USA	Preclinical study/mice, cross-neutralising activity	[247]	2022
	Modified E2 core domain (384–661aa; fused to the surface antigen of duck hepatitis B virus forming VLPs)/Chimeric VLPs	AddaVax	Freestyle 293-F	Burnet Institute, Australia; University of Melbourne, Australia; ARTES Biotechnology GmBH, Langenfeld, Germany	Preclinical study/guinea pigs, cross-neutralising activity	[246]	2022
**Immunodeficiency virus type 1 (HIV-1)/Immunodeficiency**	Prefusion-stabilised HIV-1 env trimer (Trimer 4571; BG505 DS-SOSIP.664)/Subunit	Aluminum hydroxide	CHO DG44	National Institute of Allergy and Infectious Diseases, National Institutes of Health, USA; Commissioned Corps, U.S. Public Health Service, Rockville, MD, USA	Clinical, phase 1	[253]	2022
	BG505 UFO trimer (displayed on the 1c-SApNP nanoparticles)/Subunit	AddaVax, Adju-Phos, or Alhydrogel	ExpiCHO	The Scripps Research Institute, La Jolla, USA; University of Southampton, UK, etc.	Preclinical study/mice, rabbits, and nonhuman, immunogenicity	[257]	2023
	Stabilised gp140 trimer in a prefusion conformation of clade C HIV-1 (displayed on the CoPoP liposomes)/Subunit	Adjuplex; CoPoP liposomes	HEK293E-253	Janssen Vaccines & Prevention, Leiden, The Netherlands; University of Amsterdam, Amsterdam, The Netherlands; University at Buffalo, Buffalo, USA, etc.	Preclinical study/rabbits, cross-neutralising activity	[258]	2024
**Respiratory syncytial virus (RSV)/RSV infection**	F protein in the prefusion-stabilised form/Subunit	AS01E	CHO	Arexvy /GlaxoSmithKline	Licenced	[280]	FDA 2023
	F protein in the prefusion-stabilised form (subgroups A and B)/Subunit	No information	CHO	Abrysvo/Pfizer	Licenced	[281]	FDA 2023
	Pre-F (displayed on the ferritin nanoparticles)/Subunit	AF03	CHO	Sanofi, Cambridge, USA; Sanofi Pasteur VaxDesign, USA; Sanofi Pasteur, France, etc.	Preclinical study/mice, nonhuman primates, neutralising activity	[282]	2020
	Stabilised prefusion F/Subunit	aluminum hydroxide with or without	CHO	DS-Cav1/National Institute of Allergy and Infectious Diseases, National Institutes of Health, USA	Clinical, phase 1	[262]	2021
	Pre-F or G or Pre-F or Pre-F+G antigens (displayed on the TM/CT domains of the NDV F protein forming VLPs)/Chimeric VLPs	None	Expi293F	University of Massachusetts Chan Medical School, Worcester, MA, USA, etc.	Preclinical study/cotton rats, protectiveness	[265]	2023
	Central conserved domain (CCD) of G protein (displayed on the lumazine synthase nanoparticles)/Subunit	Adjuplex	Expi293F	Janssen Vaccines & Prevention B, Leiden, The Netherlands	Preclinical study/mice, neutralising activity	[266]	2024
**Ebola virus (EBOV)/Hemorrhagic fever**	Modified GPΔmuc trimer (displayed on the ferritin or E2p or I3-01 nanoparticles)/Subunit	AddaVax or Adju-Phos (InvivoGen)	ExpiCHO (GPΔmuc), HEK293F (GPΔmuc-presenting NPs)	The Scripps Research Institute, CA, USA	Preclinical study/mice and rabbits, cross-neutralising activity	[283]	2021
	Hyperglycosylated GPΔmuc trimers (displayed on the ferritin nanoparticles)/Subunit	Ferritin, MPLA/Quil-A	Expi-293F	Stanford University School of Medicine, Stanford, USA; Boston University, Boston, USA	Preclinical study/mice, cross-neutralising activity	[267]	2024
**Kaposi sarcoma herpesvirus (KSHV)/Kaposi sarcoma**	KSHV proteins gpK8.1, gB, and gH/gL (displayed on the nanoparticles derived from the TM/CT domains of the NDV F protein)/Chimeric VLPs	Aluminum hydroxide+MPLA	CHO	Beckman Research Institute of City of Hope, USA; Masinde Muliro University of Science and Technology, Kenya	Preclinical study/ rabbits, neutralising activity	[270]	2019
**Epstein-Barr virus (EBV)/Infectious mononucleosis**	gH/gL or gH/gL/gp42 (displayed on the ferritin nanoparticles)/Subunit	SAS (Sigma Adjuvant System)	Expi293F	National Institute of Allergy and Infectious Diseases, National Institutes of Health, Bethesda, USA; Henry M. Jackson Foundation for the Advancement of Military Medicine, Bethesda, USA, etc.	Preclinical study/ mice, nonhuman primates	[275]	2019
	Glycoproteins gp350, gB, gp42, gH, and gL (displayed on the nanoparticles derived from the TM/CT domains of the NDV F protein)/Chimeric VLPs	Aluminum hydroxide+ MPLA	CHO	Department of Immuno-Oncology, Irell & Manella Graduate School of Biological Sciences of City of Hope, Duarte, USA; Beckman Research Institute of City of Hope, Duarte, USA; Masinde Muliro University of Science and Technology, Kakamega, Kenya, etc.	Preclinical study/ New Zealand white rabbits, neutralising activity	[277]	2020
	gH/gL(displayed on the ferritin or cTRP or IMX313 nanoparticles)/Subunit	SAS	HEK293	Fred Hutchinson Cancer Research Center, Seattle, WA, USA; University of Washington, Seattle, USA; Institute for Protein Design, University of Washington, Seattle, USA, etc.	Preclinical study/humanised mice, protectiveness	[278]	2022
	gH/gL/gp42+gp350 or gH/gL+gp350 (displayed on the ferritin nanoparticles)/Subunit	AF03	Expi293F	Sanofi, 640 Memorial Dr., Cambridge, CA, USA; ModeX Therapeutics Inc., USA; National Institute of Allergy and Infectious Diseases, Bethesda, MD, USA, etc.	Preclinical study/ mice, ferrets, and nonhuman primates	[276]	2022
	gp350 (displayed on the ferritin nanoparticles)/Subunit	Matrix-M1	No information	National Institute of Allergy and Infectious Diseases, Bethesda, MD, USA	Clinical, phase 1	NCT04645147	2022

## 7. Using Multiple Expression Systems in Vaccine Development

Previous sections have discussed the advantages of different expression systems. Regardless of the cost-effectiveness of some systems, others are required for the production of specific antigens. During vaccine development, multiple expression systems may be used simultaneously to achieve the optimal expression conditions for each vaccine antigen and auxiliary vaccine components (e.g., the delivery system). Table 6 provides examples of vaccine candidates whose antigens and components were produced in different expression systems.

### 7.1. Respiratory Syncytial Virus (RSV)

Kawahara and colleagues showed that *E. coli*-derived RSV G protein in combination with pre-F antigen produced in the Expi293 cell line (DS-Cav1 vaccine) with CpG ODNs as an adjuvant provided greater protection against RSV infection of the upper respiratory tract than either of these two antigens alone [284]. The production of G protein in bacterial cells turned out to be crucial. The authors carried out a comparative analysis of the immunogenicity and safety of mammal cells-derived RSV G protein (67–298 aa) and its *E. coli*-derived version without glycosylation in combination with various adjuvants (Table 6). The molecular weight of G protein obtained from *E. coli* was consistent with the estimated molecular weight calculated using the amino acid sequence (30 kDa), while at the same time, the molecular weight of G protein expressed in Expi293 mammalian cells was 90 kDa, due to hyperglycosilation. The Expi293-derived G antigen was not effective at eliciting IgGs and suppressing eosinophilia in mice, which raises doubts about the feasibility of using it in vaccines. At the same time, the nonglycosylated form of the protein elicited IgGs, even with no adjuvants, and the addition of CpG ODNs adjuvant led to high IgG titres and a Th1 cell immune response (while a Th2 cell immune response is believed to be the reason for ERD, as discussed in Section 5.3).

### 7.2. Epstein-Barr Virus (EBV or HHV-4)

Dasari and co-authors developed a subunit vaccine candidate designed to prevent primary EBV infection and control latently infected B cells [273]. The vaccine candidate included two antigens: EBV gp350 glycoprotein and genetically engineered polyepitope EBV protein containing 20 CD8+ T cells epitopes of latent and lytic EBV antigens. EBV gp350 was expressed in CHO mammalian cells, while polyepitope EBV antigen was expressed in *E. coli.* For the assessment of the vaccine’s effectiveness, the immunisation of multiple HLA transgenic mice with the vaccine candidate was performed. For this, Amphiphile-CpG adjuvant mediating antigen delivery into lymph nodes was used. The immunisation induced high titres of neutralising antibodies and polyfunctional CD4+ and CD8+ T cell responses. Table 6. Summary of some vaccines against human viral diseases, whose components were expressed in multiple different expression systems for the period from 2019 to 2023.

**Table 6:** Summary of some vaccines against human viral diseases, whose components were expressed in multiple different expression systems for the period from 2019 to 2024.

Pathogen/Disease	Vaccine Antigen/Type of Vaccine	Adjuvant	Expression System/Cell Line	Vaccine Name/Developer	Status	References or Clinical Trial Identifier (NCT)	Year Reported or Regulatory Approved
**Influenza virus/Influenza**	HA stalk, M2e (displayed on the SAPNs protein nanocages)/Subunit	None	Expi293F (HA) *E. coli* (M2e; component of SAPNs)	Georgia Institute of Technology, Atlanta, GA, USA	Preclinical study/mice, immunogenicity	[285]	2023
**Respiratory syncytial virus (RSV)/RSV infection**	Ectodomain G and Pre-F protein/Subunit	alum, CpG ODN or AddaVax.	Expi293 (G and Pre-F) *E. coli* (G)	Osaka University, Osaka, Japan; Tokyo Medical University, Tokyo, Japan	Preclinical study/mice, protectiveness	[284]	2023
**Epstein-Barr virus (EBV)/Infectious mononucleosis**	EBV gp350 and polyepitope protein (20 CD8+ T cell epitopes)/Subunit	Amphiphile-CpG	CHO (gp350) *E. coli* (polyepitope protein)	Berghofer Medical Research Institute, Herston, Australia Elicio Therapeutics, Inc., Boston, MA, USA	Preclinical study/ HLA expressing mice, neutralising activity of Ab and T-cell responses	[273]	2023

## 8. Conclusions

Recombinant protein vaccines have proven their efficacy and safety, earning a place in public vaccination programmes against numerous viral infections worldwide. In combatting the COVID-19 pandemic, these vaccines demonstrated a level of protection comparable with vector and RNA vaccines, successfully preventing the spread of the coronavirus and reducing the risks of severe illness and death [286]. This success has sparked interest in developing recombinant vaccine candidates against other relevant viral infections. Efforts are now focused on creating vaccines for diseases that currently lack licensed options, as well as on expanding the arsenal of existing vaccines for certain diseases. A distinctive feature, and fundamental advantage, of recombinant vaccines is their exceptional safety profile. The absence of whole virions (attenuated or inactivated) in these vaccines eliminates the risk of infection and reduces the likelihood of contamination with other pathogens and side effects, making these vaccines particularly useful for immunocompromised individuals and older people. In addition, the ability to quickly modify the amino acid composition of recombinant proteins in response to rapid mutational changes in the pathogen, together with an established production process, makes this approach competitive during epidemics and pandemics. This is especially advantageous in the early stages of such events, when other vaccine platforms may require additional safety testing.

Choosing the appropriate expression system for producing the antigen is a critical step in the creation of recombinant vaccines. Each expression system has its own unique features related to protein folding and stability, as well as a distinctive glycosylation pattern, so selection should be guided by the specific requirements for the antigenic protein, such as its size, compositional complexity and essential post-translational modifications, if any. It is important to note, however, that an increasing number of technologies are emerging that allow the adaptation of ‘traditional’ expression systems to meet the evolving needs of modern researchers. For instance, there are several approaches for developing expression systems capable of reproducing the necessary glycosylation pattern, including methods developed specifically for prokaryotic systems. Therefore, multiple expression systems are often suitable for producing the vaccine antigen, and it is crucial to select the one that achieves the highest immunogenicity and protective efficacy of the resulting protein. When choosing an expression platform, it is also necessary to consider factors that are essential for industrial-scale vaccine production, such as cost-effectiveness, scalability and the ease of culturing. Many vaccines fail to reach commercial use due to the high costs and/or specific expression conditions required. Therefore, it is vitally important to select a system that is as robust and uncomplicated as possible, while still meeting all the requirements for producing the desired antigen.

In addition to full-length viral proteins, individual sequences of virus neutralising epitopes are also used as the basis for recombinant vaccines, allowing the production of the safest and the most stable vaccine candidates. This approach can also enhance vaccine efficacy by eliciting a targeted immune response and reducing the risks of adverse effects, including antibody-dependent enhancement of infection.

Recombinant antigens, whether full-length proteins or individual sequences, are often not sufficiently immunogenic on their own. Therefore, the selection of an appropriate adjuvant becomes another determining step in the development of a recombinant vaccine. The right adjuvant can significantly enhance the vaccine’s ability to elicit a robust, long-lasting, specific and protective immune response. Currently, adjuvants based on virus-like particles are becoming increasingly prevalent, allowing for the development of vaccines with improved immunogenicity due to more effective antigen presentation. Moreover, some VLPs can affect the stability of the antigen and even the type of immune response induced, which can be vital for vaccines against certain viral infections.

It is worth noting that recombinant protein vaccines are currently being considered not only as stand-alone options, but also as boosters to maintain immunity levels after primary vaccination, protecting the population against reinfection. Booster doses of recombinant vaccines can also serve as a crucial tool for adapting to new threats when new pathogen variants emerge or epidemiological conditions change. This can be particularly beneficial for protecting vulnerable groups, such as older people, individuals with a chronic disease and those with a weakened immune system, who are at higher risk of developing severe forms of disease. Therefore, the use of recombinant vaccines as boosters could be a vital strategic decision, which is capable of strengthening population immunity and providing maximum protection against infectious diseases, ultimately contributing to better public health outcomes.

## Data Availability

No new data were created or analyzed in this study.

## References

[1] Lawrence P., Heung M., Nave J., Henkel C., Escudero-Pérez B. (2023). The natural virome and pandemic potential: Disease X. Curr. Opin. Virol..

[2] Kovalenko A., Ryabchevskaya E., Evtushenko E., Nikitin N., Karpova O. (2023). Recombinant Protein Vaccines against Human Betacoronaviruses: Strategies, Approaches and Progress. Int. J. Mol. Sci..

[3] Jayakrishnan A., Wan Rosli W.R., Tahir A.R.M., Razak F.S.A., Kee P.E., Ng H.S., Chew Y.-L., Lee S.-K., Ramasamy M., Tan C.S. (2024). Evolving Paradigms of Recombinant Protein Production in Pharmaceutical Industry: A Rigorous Review. Sci.

[4] Zhang L., Xu W., Ma X., Li Y., Chen J., Wang Z. (2023). Virus-like Particles as Antiviral Vaccine: Mechanism, Design, and Application. Biotechnol. Bioprocess. Eng..

[5] Tan C.W., Valkenburg S.A., Poon L.L.M., Wang L.-F. (2023). Broad-spectrum pan-genus and pan-family virus vaccines. Cell Host Microbe.

[6] Cid R., Bolivar J. (2021). Platforms for Production of Protein-Based Vaccines: From Classical to Next-Generation Strategies. Biomolecules.

[7] Zhao T., Cai Y., Jiang Y., Li Z., Liu M., Zhang X., Wang W. (2023). Vaccine adjuvants: Mechanisms and platforms. Sig. Transduct. Target. Ther..

[8] Pollet J., Chen W.-H., Strych U. (2021). Recombinant protein vaccines, a proven approach against coronavirus pandemics. Adv. Drug Deliv. Rev..

[9] Heidary M., Kaviar V.H., Shirani M., Ghanavati R., Motahar M., Sholeh M., Ghahramanpour H., Khoshnood S. (2022). A Comprehensive Review of the Protein Subunit Vaccines Against COVID-19. Front. Microbiol..

[10] Sharifzadeh M., Mottaghi-Dastjerdi N., Soltany Rezae Raad M. (2022). A Review of Virus-Like Particle-Based SARS-CoV-2 Vaccines in Clinical Trial Phases. Iran J. Pharm. Res..

[11] Suryawanshi Y.R. (2023). An overview of protein-based SARS-CoV-2 vaccines. Vaccine.

[12] Chavda V.P., Ghali E.N.H.K., Balar P.C., Chauhan S.C., Tiwari N., Shukla S., Athalye M., Patravale V., Apostolopoulos V., Yallapu M.M. (2024). Protein subunit vaccines: Promising frontiers against COVID-19. J. Control. Release.

[13] Souza C.C., Guimarães J.M., Pereira S.D.S., Mariúba L.A.M. (2021). The multifunctionality of expression systems in Bacillus subtilis: Emerging devices for the production of recombinant proteins. Exp. Biol. Med..

[14] Retallack D.M., Jin H., Chew L. (2012). Reliable protein production in a Pseudomonas fluorescens expression system. Protein Expr. Purif..

[15] Gauttam R., Eng T., Zhao Z., Ul Ain Rana Q., Simmons B.A., Yoshikuni Y., Mukhopadhyay A., Singer S.W. (2023). Development of genetic tools for heterologous protein expression in a pentose-utilizing environmental isolate of Pseudomonas putida. Microb. Biotechnol..

[16] Gruber S., Schwab H., Koefinger P. (2015). Versatile plasmid-based expression systems for Gram-negative bacteria--General essentials exemplified with the bacterium Ralstonia eutropha H16. New Biotechnol..

[17] Frelet-Barrand A. (2022). *Lactococcus lactis*, an Attractive Cell Factory for the Expression of Functional Membrane Proteins. Biomolecules.

[18] Niazi S.K., Magoola M. (2023). Advances in *Escherichia coli*-Based Therapeutic Protein Expression: Mammalian Conversion, Continuous Manufacturing, and Cell-Free Production. Biologics.

[19] Wakelin S.J., Sabroe I., Gregory C.D., Poxton I.R., Forsythe J.L., Garden O.J., Howie S.E. (2006). “Dirty little secrets”–Endotoxin contamination of recombinant proteins. Immunol. Lett..

[20] Gorbet M.B., Sefton M.V. (2005). Endotoxin: The uninvited guest. Biomaterials.

[21] Levy R., Weiss R., Chen G., Iverson B.L., Georgiou G. (2001). Production of correctly folded Fab antibody fragment in the cytoplasm of *Escherichia coli* trxB gor mutants via the coexpression of molecular chaperones. Protein Expr. Purif..

[22] Lobstein J., Emrich C.A., Jeans C., Faulkner M., Riggs P., Berkmen M. (2012). SHuffle, a novel *Escherichia coli* protein expression strain capable of correctly folding disulfide bonded proteins in its cytoplasm. Microb. Cell Fact..

[23] Wang K., Zhou L., Chen T., Li Q., Li J., Liu L., Li Y., Sun J., Li T., Wang Y. (2021). Engineering for an HPV 9-valent vaccine candidate using genomic constitutive over-expression and low lipopolysaccharide levels in *Escherichia coli* cells. Microb. Cell Fact..

[24] Mamat U., Wilke K., Bramhill D., Schromm A.B., Lindner B., Kohl T.A., Corchero J.L., Villaverde A., Schaffer L., Head S.R. (2015). Detoxifying *Escherichia coli* for endotoxin-free production of recombinant proteins. Microb. Cell Fact..

[25] Chene A., Gangnard S., Guadall A., Ginisty H., Leroy O., Havelange N., Viebig N.K., Gamain B. (2019). Preclinical immunogenicity and safety of the cGMP-grade placental malaria vaccine PRIMVAC. EBioMedicine.

[26] Sirima S.B., Richert L., Chêne A., Konate A.T., Campion C., Dechavanne S., Semblat J.P., Benhamouda N., Bahuaud M., Loulergue P. (2020). PRIMVAC vaccine adjuvanted with Alhydrogel or GLA-SE to prevent placental malaria: A first-in-human, randomised, double-blind, placebo-controlled study. Lancet Infect. Dis..

[27] Wacker M., Linton D., Hitchen P.G., Nita-Lazar M., Haslam S.M., North S.J., Panico M., Morris H.R., Dell A., Wren B.W. (2002). N-linked glycosylation in Campylobacter jejuni and its functional transfer into *E. coli*. Science.

[28] Nothaft H., Szymanski C.M. (2019). New discoveries in bacterial N-glycosylation to expand the synthetic biology toolbox. Curr. Opin. Chem. Biol..

[29] Szymanski C.M. (2022). Bacterial glycosylation, it’s complicated. Front. Mol. Biosci..

[30] Knoot C.J., Wantuch P.L., Robinson L.S., Rosen D.A., Scott N.E., Harding C.M. (2023). Discovery and characterization of a new class of O-linking oligosaccharyltransferases from the Moraxellaceae family. Glycobiology.

[31] Valderrama-Rincon J.D., Fisher A.C., Merritt J.H., Fan Y.-Y., Reading C.A., Chhiba K., Heiss C., Azadi P., Aebi M., DeLisa M.P. (2012). An engineered eukaryotic protein glycosylation pathway in *Escherichia coli*. Nat. Chem. Biol..

[32] Lauber J., Handrick R., Leptihn S., Dürre P., Gaisser S. (2015). Expression of the functional recombinant human glycosyltransferase GalNAcT2 in Escherichia coli. Microb. Cell Fact..

[33] Srichaisupakit A., Ohashi T., Misaki R., Fujiyama K. (2015). Production of initial-stage eukaryotic N-glycan and its protein glycosylation in *Escherichia coli*. J. Biosci. Bioeng..

[34] Harding C.M., Feldman M.F. (2019). Glycoengineering bioconjugate vaccines, therapeutics, and diagnostics in *E. coli*. Glycobiology.

[35] Romano M.R., Berti F., Rappuoli R. (2022). Classical- and bioconjugate vaccines: Comparison of the structural properties and immunological response. Curr. Opin. Immunol..

[36] Lim C.M.L., Komarasamy T.V., Adnan N.A.A.B., Radhakrishnan A.K., Balasubramaniam V.R.M.T. (2024). Recent Advances, Approaches and Challenges in the Development of Universal Influenza Vaccines. Influenza Other Respir. Viruses.

[37] Erbelding E.J., Post D.J., Stemmy E.J., Roberts P.C., Augustine A.D., Ferguson S., Paules C.I., Graham B.S., Fauci A.S. (2018). A Universal Influenza Vaccine: The Strategic Plan for the National Institute of Allergy and Infectious Diseases. J. Infect. Dis..

[38] Deng L., Cho K.J., Fiers W., Saelens X. (2015). M2e-Based Universal Influenza A Vaccines. Vaccines.

[39] Tsybalova L.M., Stepanova L.A., Kuprianov V.V., Blokhina E.A., Potapchuk M.V., Korotkov A.V., Gorshkov A.N., Kasyanenko M.A., Ravin N.V., Kiselev O.I. (2015). Development of a candidate influenza vaccine based on virus-like particles displaying influenza M2e peptide into the immunodominant region of hepatitis B core antigen: Broad protective efficacy of particles carrying four copies of M2e. Vaccine.

[40] Ong H.K., Yong C.Y., Tan W.S., Yeap S.K., Omar A.R., Abdul Razak M., Ho K.L. (2019). An Influenza A Vaccine Based on the Extracellular Domain of Matrix 2 Protein Protects BALB/C Mice Against H1N1 and H3N2. Vaccines.

[41] Li M., Guo P., Chen C., Feng H., Zhang W., Gu C., Wen G., Rao V.B., Tao P. (2021). Bacteriophage T4 Vaccine Platform for Next-Generation Influenza Vaccine Development. Front. Immunol..

[42] Zhao Y., Li Z., Voyer J., Li Y., Chen X. (2022). Flagellin/Virus-like Particle Hybrid Platform with High Immunogenicity, Safety, and Versatility for Vaccine Development. ACS Appl. Mater. Interfaces.

[43] Hayward A.C., Wang L., Goonetilleke N., Fragaszy E.B., Bermingham A., Copas A., Dukes O., Millett E.R., Nazareth I., Nguyen-Van-Tam J.S. (2015). Natural T Cell-mediated Protection against Seasonal and Pandemic Influenza. Results of the Flu Watch Cohort Study. Am. J. Respir. Crit. Care Med..

[44] Atsmon J., Caraco Y., Ziv-Sefer S., Shaikevich D., Abramov E., Volokhov I., Bruzil S., Haima K.Y., Gottlieb T., Ben-Yedidia T. (2014). Priming by a novel universal influenza vaccine (Multimeric-001)-a gateway for improving immune response in the elderly population. Vaccine.

[45] van Doorn E., Liu H., Ben-Yedidia T., Hassin S., Visontai I., Norley S., Frijlink H.W., Hak E. (2017). Evaluating the immunogenicity and safety of a BiondVax-developed universal influenza vaccine (Multimeric-001) either as a standalone vaccine or as a primer to H5N1 influenza vaccine: Phase IIb study protocol. Medicine.

[46] Atmar R.L., Bernstein D.I., Winokur P., Frey S.E., Angelo L.S., Bryant C., Ben-Yedidia T., Roberts P.C., El Sahly H.M., Keitel W.A. (2023). Safety and immunogenicity of Multimeric-001 (M-001) followed by seasonal quadrivalent inactivated influenza vaccine in young adults—A randomized clinical trial. Vaccine.

[47] Zykova A.A., Blokhina E.A., Stepanova L.A., Shuklina M.A., Tsybalova L.M., Kuprianov V.V., Ravin N.V. (2022). Nanoparticles based on artificial self-assembling peptide and displaying M2e peptide and stalk HA epitopes of influenza A virus induce potent humoral and T-cell responses and protect against the viral infection. Nanomedicine.

[48] Zykova A.A., Blokhina E.A., Stepanova L.A., Shuklina M.A., Ozhereleva O.O., Tsybalova L.M., Kuprianov V.V., Ravin N.V. (2023). Nanoparticles Carrying Conserved Regions of Influenza A Hemagglutinin, Nucleoprotein, and M2 Protein Elicit a Strong Humoral and T Cell Immune Response and Protect Animals from Infection. Molecules.

[49] Hu Y.M., Bi Z.F., Zheng Y., Zhang L., Zheng F.Z., Chu K., Li Y.F., Chen Q., Quan J.L., Hu X.W. (2023). Immunogenicity and safety of an *Escherichia coli*-produced human papillomavirus (types 6/11/16/18/31/33/45/52/58) L1 virus-like-particle vaccine: A phase 2 double-blind, randomized, controlled trial. Sci. Bull..

[50] Zhu F.C., Zhong G.H., Huang W.J., Chu K., Zhang L., Bi Z.F., Zhu K.X., Chen Q., Zheng T.Q., Zhang M.-L. (2023). Head-to-head immunogenicity comparison of an *Escherichia coli*-produced 9-valent human papillomavirus vaccine and Gardasil 9 in women aged 18-26 years in China: A randomised blinded clinical trial. Lancet Infect. Dis..

[51] Gameiro S.F., Mymryk J.S. (2022). Special Issue “Human Papillomavirus Clinical Research: From Infection to Cancer”. J. Clin. Med..

[52] Li Z., Song S., He M., Wang D., Shi J., Liu X., Li Y., Chi X., Wei S., Yang Y. (2018). Rational design of a triple-type human papillomavirus vaccine by compromising viral-type specificity. Nat. Commun..

[53] Qian C., Yang Y., Xu Q., Wang Z., Chen J., Chi X., Yu M., Gao F., Xu Y., Lu Y. (2022). Characterization of an *Escherichia coli*-derived triple-type chimeric vaccine against human papillomavirus types 39, 68 and 70. NPJ Vaccines.

[54] Yu M., Chi X., Huang S., Wang Z., Chen J., Qian C., Han F., Cao L., Li J., Sun H. (2022). A bacterially expressed triple-type chimeric vaccine against human papillomavirus types 51, 69, and 26. Vaccine.

[55] Zhai L., Yadav R., Kunda N.K., Anderson D., Bruckner E., Miller E.K., Basu R., Muttil P., Tumban E. (2019). Oral immunization with bacteriophage MS2-L2 VLPs protects against oral and genital infection with multiple HPV types associated with head & neck cancers and cervical cancer. Antiviral Res..

[56] Yadav R., Zhai L., Kunda N.K., Muttil P., Tumban E. (2021). Mixed Bacteriophage MS2-L2 VLPs Elicit Long-Lasting Protective Antibodies against HPV Pseudovirus 51. Viruses.

[57] Mashhadi Abolghasem Shirazi M., Sadat S.M., Haghighat S., Roohvand F., Arashkia A. (2023). Alum and a TLR7 agonist combined with built-in TLR4 and 5 agonists synergistically enhance immune responses against HPV RG1 epitope. Sci. Rep..

[58] Groome M.J., Fairlie L., Morrison J., Fix A., Koen A., Masenya M., Jose L., Madhi S.A., Page N., McNeal M. (2020). Safety and immunogenicity of a parenteral trivalent P2-VP8 subunit rotavirus vaccine: A multisite, randomised, double-blind, placebo-controlled trial. Lancet Infect. Dis..

[59] PATH. https://www.path.org/our-impact/media-center/path-announces-early-closure-of-pivotal-phase-3-study-of-an-injectable-rotavirus-vaccine-candidate/.

[60] Velasquez D.E., Jiang B. (2019). Evolution of P[8], P[4], and P[6] VP8* genes of human rotaviruses globally reported during 1974 and 2017: Possible implications for rotavirus vaccines in development. Hum. Vaccin. Immunother..

[61] Kondakova O.A., Ivanov P.A., Baranov O.A., Ryabchevskaya E.M., Arkhipenko M.V., Skurat E.V., Evtushenko E.A., Nikitin N.A., Karpova O.V. (2021). Novel antigen panel for modern broad-spectrum recombinant rotavirus A vaccine. Clin. Exp. Vaccine Res..

[62] Granovskiy D.L., Khudainazarova N.S., Evtushenko E.A., Ryabchevskaya E.M., Kondakova O.A., Arkhipenko M.V., Kovrizhko M.V., Kolpakova E.P., Tverdokhlebova T.I., Nikitin N.A. (2024). Novel Universal Recombinant Rotavirus A Vaccine Candidate: Evaluation of Immunological Properties. Viruses.

[63] Xia M., Huang P., Tan M. (2022). A Pseudovirus Nanoparticle-Based Trivalent Rotavirus Vaccine Candidate Elicits High and Cross P Type Immune Response. Pharmaceutics.

[64] Xia M., Huang P., Vago F., Kawagishi T., Ding S., Greenberg H.B., Jiang W., Tan M. (2024). A Viral Protein 4-Based Trivalent Nanoparticle Vaccine Elicited High and Broad Immune Responses and Protective Immunity against the Predominant Rotaviruses. ACS Nano.

[65] Cabral-Miranda G., Lim S.M., Mohsen M.O., Pobelov I.V., Roesti E.S., Heath M.D., Skinner M.A., Kramer M.F., Martina B.E.E., Bachmann M.F. (2019). Zika Virus-Derived E-DIII Protein Displayed on Immunologically Optimized VLPs Induces Neutralizing Antibodies without Causing Enhancement of Dengue Virus Infection. Vaccines.

[66] Gabutti G., Bolognesi N., Sandri F., Florescu C., Stefanati A. (2019). Varicella zoster virus vaccines: An update. Immunotargets Ther..

[67] Sun Y., Kim E., Kong C.L., Arnold B.F., Porco T.C., Acharya N.R. (2021). Effectiveness of the Recombinant Zoster Vaccine in Adults Aged 50 and Older in the United States: A Claims-Based Cohort Study. Clin. Infect. Dis..

[68] Losa L., Antonazzo I.C., Di Martino G., Mazzaglia G., Tafuri S., Mantovani L.G., Ferrara P. (2024). Immunogenicity of Recombinant Zoster Vaccine: A Systematic Review, Meta-Analysis, and Meta-Regression. Vaccines.

[69] U.S. Food and Drug Administration. https://www.fda.gov/media/108597/download.

[70] Chen T., Sun J., Zhang S., Li T., Liu L., Xue W., Zhou L., Liang S., Yu Z., Zheng Q. (2023). Truncated glycoprotein E of varicella-zoster virus is an ideal immunogen for *Escherichia coli*-based vaccine design. Sci. China Life Sci..

[71] Liu J., Zhu R., Ye X., Yang L., Wang Y., Huang Y., Wu J., Wang W., Ye J., Li Y. (2015). A monoclonal antibody-based VZV glycoprotein E quantitative assay and its application on antigen quantitation in VZV vaccine. Appl. Microbiol. Biotechnol..

[72] Lynch J.A., Lim J.K., Asaga P.E.P., Wartel T.A., Marti M., Yakubu B., Rees H., Talaat K., Kmush B., Aggarwal R. (2023). Hepatitis E vaccine-Illuminating the barriers to use. PLoS Negl. Trop. Dis..

[73] Cao Y.F., Tao H., Hu Y.M., Shi C.B., Wu X., Liang Q., Chi C.P., Li L., Liang Z.L., Meng J.H. (2017). A phase 1 randomized open-label clinical study to evaluate the safety and tolerability of a novel recombinant hepatitis E vaccine. Vaccine.

[74] Li Y., Huang X., Zhang Z., Li S., Zhang J., Xia N., Zhao Q. (2020). Prophylactic Hepatitis E Vaccines: Antigenic Analysis and Serological Evaluation. Viruses.

[75] Kim H.W., Canchola J.G., Brandt C.D., Pyles G., Chanock R.M., Jensen K., Parrott R.H. (1969). Respiratory syncytial virus disease in infants despite prior administration of antigenic inactivated vaccine. Am. J. Epidemiol..

[76] Murphy B.R., Walsh E.E. (1988). Formalin-inactivated respiratory syncytial virus vaccine induces antibodies to the fusion glycoprotein that are deficient in fusion-inhibiting activity. J. Clin. Microbiol..

[77] Bergeron H.C., Tripp R.A. (2021). Immunopathology of RSV: An Updated Review. Viruses.

[78] Mazur N.I., Higgins D., Nunes M.C., Melero J.A., Langedijk A.C., Horsley N., Buchholz U.J., Openshaw P.J., van der Klis F., Karron R. (2023). Respiratory Syncytial Virus Prevention Within Reach: The Vaccine and Monoclonal Antibody Landscape. Lancet Infect. Dis..

[79] Langley J.M., Aggarwal N., Toma A., Halperin S.A., McNeil S.A., Côté E., Boulay I., Guasparini R., Stiver G., Embree J. (2018). A Respiratory Syncytial Virus Vaccine Based on the Small Hydrophobic Protein Ectodomain Presented with a Novel Lipid-Based Formulation Is Highly Immunogenic and Safe in Adults: A First-in-Humans Study. J. Infect. Dis..

[80] Schepens B., Sedeyn K., Vande Ginste L., De Baets S., Schotsaert M., Roose K., Houspie L., Van Ranst M., Gilbert B., van Rooijen N. (2014). Protection and Mechanism of Action of a Novel Human Respiratory Syncytial Virus Vaccine Candidate Based on the Extracellular Domain of Small Hydrophobic Protein. EMBO Mol. Med..

[81] Umemoto S., Nakahashi-Ouchida R., Yuki Y., Kurokawa S., Machita T., Uchida Y., Mori H., Yamanoue T., Shibata T., Sawada S.I. (2023). Cationic-Nanogel Nasal Vaccine Containing the Ectodomain of RSV-Small Hydrophobic Protein Induces Protective Immunity in Rodents. NPJ Vaccines.

[82] DCVMN. https://dcvmn.org/member/innovax/.

[83] Luo G., Zeng Y., Yang H., Li Y., Yang L., Li C., Song F., Zhang S., Li T., Ge S. (2022). Bivalent rotavirus VP4∗ stimulates protective antibodies against common genotypes of human rotaviruses. iScience.

[84] Brady J.R., Whittaker C.A., Tan M.C., Kristensen D.L., Ma D., Dalvie N.C., Love K.R., Love J.C. (2020). Comparative genome-scale analysis of Pichia pastoris variants informs selection of an optimal base strain. Biotechnol. Bioeng..

[85] Gündüz Ergün B., Hüccetoğulları D., Öztürk S., Çelik E., Çalık P. (2019). Established and Upcoming Yeast Expression Systems. Methods Mol. Biol..

[86] Schütz A., Bernhard F., Berrow N., Buyel J.F., Ferreira-da-Silva F., Haustraete J., van den Heuvel J., Hoffmann J.-E., de Marco A., Peleg Y. (2023). A concise guide to choosing suitable gene expression systems for recombinant protein production. STAR Protoc..

[87] Khlebodarova T.M., Bogacheva N.V., Zadorozhny A.V., Bryanskaya A.V., Vasilieva A.R., Chesnokov D.O., Pavlova E.I., Peltek S.E. (2024). Komagataella phaffii as a Platform for Heterologous Expression of Enzymes Used for Industry. Microorganisms.

[88] Stephenne J. (1992). Contribution to hepatitis B prevention. Vaccine.

[89] Ellis R.W., Gerety R.J. (1989). Plasma-derived and yeast-derived hepatitis B vaccines. Am. J. Infect. Control..

[90] Jackson S., Lentino J., Kopp J., Murray L., Ellison W., Rhee M., Shockey G., Akella L., Erby K., Heyward W.L. (2018). Immunogenicity of a two-dose investigational hepatitis B vaccine, HBsAg-1018, using a toll-like receptor 9 agonist adjuvant compared with a licensed hepatitis B vaccine in adults. Vaccine.

[91] Hernández-Bernal F., Aguilar-Betancourt A., Aljovin V., Arias G., Valenzuela C., de Alejo K.P., Hernández K., Oquendo O., Figueredo N., Figueroa N. (2011). Comparison of four recombinant hepatitis B vaccines applied on an accelerated schedule in healthy adults. Hum. Vaccin..

[92] Monie A., Hung C.-F., Roden R., Wu T.-C. (2008). Cervarix: A vaccine for the prevention of HPV 16, 18-associated cervical cancer. Biologics.

[93] Hansen J., Yee A., Lewis N., Li S., Velicer C., Saddier P., Klein N.P. (2023). Safety of 9-valent human papillomavirus vaccine administered to males and females in routine use. Vaccine.

[94] Zhao C., Zhao Y., Li J., Li M., Shi Y., Wei L. (2024). Opportunities and challenges for human papillomavirus vaccination in China. Hum. Vaccin. Immunother..

[95] Kumar R., Kumar P. (2019). Yeast-based vaccines: New perspective in vaccine development and application. FEMS Yeast Res..

[96] Srivastava V., Nand K.N., Ahmad A., Kumar R. (2023). Yeast-Based Virus-like Particles as an Emerging Platform for Vaccine Development and Delivery. Vaccines.

[97] Wang S.C., Liao H.Y., Zhang J.Y., Cheng T.R., Wong C.H. (2019). Development of a universal influenza vaccine using hemagglutinin stem protein produced from Pichia pastoris. Virology.

[98] Dutta S.K., Langenburg T. (2023). A Perspective on Current Flavivirus Vaccine Development: A Brief Review. Viruses.

[99] Halstead S.B. (2017). Dengvaxia sensitizes seronegatives to vaccine enhanced disease regardless of age. Vaccine.

[100] (2018). Global Advisory Committee on Vaccine Safety, 6–7 December 2017. Wkly. Epidemiol. Rec..

[101] Shanmugam R.K., Ramasamy V., Shukla R., Arora U., Swaminathan S., Khanna N. (2019). Pichia pastoris-expressed Zika virus envelope domain III on a virus-like particle platform: Design, production and immunological evaluation. Pathog. Dis..

[102] Ramasamy V., Arora U., Shukla R., Poddar A., Shanmugam R.K., White L.J., Mattocks M.M., Raut R., Perween A., Tyagi P. (2018). A tetravalent virus-like particle vaccine designed to display domain III of dengue envelope proteins induces multi-serotype neutralizing antibodies in mice and macaques which confer protection against antibody dependent enhancement in AG129 mice. PLoS Negl. Trop. Dis..

[103] Yang L., Xiao A., Wang H., Zhang X., Zhang Y., Li Y., Wei Y., Liu W., Chen C. (2022). A VLP-Based Vaccine Candidate Protects Mice against Japanese Encephalitis Virus Infection. Vaccines.

[104] Seesen M., Jearanaiwitayakul T., Limthongkul J., Midoeng P., Sunintaboon P., Ubol S. (2023). A bivalent form of nanoparticle-based dengue vaccine stimulated responses that potently eliminate both DENV-2 particles and DENV-2-infected cells. Vaccine.

[105] Seesen M., Jearanaiwitayakul T., Limthongkul J., Sunintaboon P., Ubol S. (2022). Mice immunized with trimethyl chitosan nanoparticles containing DENV-2 envelope domain III elicit neutralizing antibodies with undetectable antibody-dependent enhancement activity. J. Gen. Virol..

[106] Gupta J., Kaul S., Srivastava A., Kaushik N., Ghosh S., Sharma C., Batra G., Banerjee M., Shalimar, Nayak B. (2020). Expression, Purification and Characterization of the Hepatitis E Virus Like-Particles in the Pichia pastoris. Front. Microbiol..

[107] Gupta J., Kumar A., Surjit M. (2022). Production of a Hepatitis E Vaccine Candidate Using the Pichia pastoris Expression System. Methods Mol. Biol..

[108] Zhang W., Qu P., Li D., Zhang C., Liu Q., Zou G., Dupont-Rouzeyrol M., Lavillette D., Jin X., Yin F. (2019). Yeast-produced subunit protein vaccine elicits broadly neutralizing antibodies that protect mice against Zika virus lethal infection. Antiviral Res..

[109] Daniell H., Kulis M., Herzog R.W. (2019). Plant cell-made protein antigens for induction of Oral tolerance. Biotechnol. Adv..

[110] An Y., Wang Y., Wang X., Xiao J. (2022). Development of chloroplast transformation and gene expression regulation technology in land plants. Front. Plant Sci..

[111] Nosaki S., Hoshikawa K., Ezura H., Miura K. (2021). Transient protein expression systems in plants and their applications. Plant Biotechnol..

[112] Komarova T.V., Baschieri S., Donini M., Marusic C., Benvenuto E., Dorokhov Y.L. (2010). Transient expression systems for plant-derived biopharmaceuticals. Expert Rev. Vaccines.

[113] Hefferon K. (2017). Plant Virus Expression Vectors: A Powerhouse for Global Health. Biomedicines.

[114] Hefferon K. (2014). Plant virus expression vector development: New perspectives. Biomed. Res. Int..

[115] Rybicki E.P., Martin D.P. (2014). Virus-derived ssDNA vectors for the expression of foreign proteins in plants. Curr. Top. Microbiol. Immunol..

[116] Tyurin A.A., Suhorukova A.V., Kabardaeva K.V., Goldenkova-Pavlova I.V. (2020). Transient Gene Expression is an Effective Experimental Tool for the Research into the Fine Mechanisms of Plant Gene Function: Advantages, Limitations, and Solutions. Plants.

[117] Marillonnet S., Giritch A., Gils M., Kandzia R., Klimyuk V., Gleba Y. (2004). In planta engineering of viral RNA replicons: Efficient assembly by recombination of DNA modules delivered by Agrobacterium. Proc. Natl. Acad. Sci. USA.

[118] Huang Z., Santi L., LePore K., Kilbourne J., Arntzen C.J., Mason H.S. (2006). Rapid, high-level production of hepatitis B core antigen in plant leaf and its immunogenicity in mice. Vaccine.

[119] Tusé D., Malm M., Tamminen K., Diessner A., Thieme F., Jarczowski F., Blazevic V., Klimyuk V. (2022). Safety and immunogenicity studies in animal models support clinical development of a bivalent norovirus-like particle vaccine produced in plants. Vaccine.

[120] Leroux-Roels I., Maes C., Joye J., Jacobs B., Jarczowski F., Diessner A., Janssens Y., Waerlop G., Tamminen K., Heinimäki S. (2022). A randomized, double-blind, placebo-controlled, dose-escalating phase I trial to evaluate safety and immunogenicity of a plant-produced, bivalent, recombinant norovirus-like particle vaccine. Front. Immunol..

[121] Klimyuk V., Pogue G., Herz S., Butler J., Haydon H. (2012). Production of Recombinant Antigens and Antibodies in Nicotiana benthamiana Using “Magnifection” Technology: GMP-Compliant Facilities for Small- and Large-Scale Manufacturing. Curr. Top. Microbiol. Immunol..

[122] Musiychuk K., Stephenson N., Bi H., Farrance C.E., Orozovic G., Brodelius M., Brodelius P., Horsey A., Ugulava N., Shamloul A.-M. (2007). A launch vector for the production of vaccine antigens in plants. Influenza Other Respir. Viruses.

[123] Chichester J.A., Jones R.M., Green B.J., Stow M., Miao F., Moonsammy G., Streatfield S.J., Yusibov V. (2012). Safety and immunogenicity of a plant-produced recombinant hemagglutinin-based influenza vaccine (HAI-05) derived from A/Indonesia/05/2005 (H5N1) influenza virus: A phase 1 randomized, double-blind, placebo-controlled, dose-escalation study in healthy adults. Viruses.

[124] Cummings J.F., Guerrero M.L., Moon J.E., Waterman P., Nielsen R.K., Jeferson S., Gross F.L., Hancock K., Katz J.M., Yusibov V. (2014). Safety and immunogenicity of a plantproduced recombinant monomer hemagglutinin-based infuenza vaccine derived from infuenza A (H1N1)Pdm09 virus: A phase 1 dose-escalation study in healthy adults. Vaccine.

[125] Mardanova E.S., Blokhina E.A., Tsybalova L.M., Peyret H., Lomonossoff G.P., Ravin N.V. (2017). Efficient Transient Expression of Recombinant Proteins in Plants by the Novel pEff Vector Based on the Genome of Potato Virus X. Front. Plant Sci..

[126] Mardanova E.S., Kotlyarov R.Y., Stuchinskaya M.D., Nikolaeva L.I., Zahmanova G., Ravin N.V. (2022). High-Yield Production of Chimeric Hepatitis E Virus-Like Particles Bearing the M2e Influenza Epitope and Receptor Binding Domain of SARS-CoV-2 in Plants Using Viral Vectors. Int. J. Mol. Sci..

[127] Sainsbury F., Thuenemann E.C., Lomonossoff G.P. (2009). pEAQ: Versatile expression vectors for easy and quick transient expression of heterologous proteins in plants. Plant Biotechnol. J..

[128] Peyret H., Brown J.K.M., Lomonossoff G.P. (2019). Improving plant transient expression through the rational design of synthetic 5′ and 3′ untranslated regions. Plant Methods.

[129] Margolin E., Oh Y.J., Verbeek M., Naude J., Ponndorf D., Meshcheriakova Y.A., Peyret H., van Diepen M.T., Chapman R., Meyers A.E. (2020). Co-expression of Human Calreticulin Significantly Improves the Production of HIV Gp140 and Other Viral Glycoproteins in Plants. Plant Biotechnol. J..

[130] Ponndorf D., Meshcheriakova Y., Thuenemann E.C., Dobon Alonso A., Overman R., Holton N., Dowall S., Kennedy E., Stocks M., Lomonossoff G.P. (2021). Plant-made dengue virus-like particles produced by co-expression of structural and non-structural proteins induce a humoral immune response in mice. Plant Biotechnol. J..

[131] Zahmanova G., Mazalovska M., Takova K., Toneva V., Minkov I., Peyret H., Lomonossoff G. (2021). Efficient Production of Chimeric Hepatitis B Virus-Like Particles Bearing an Epitope of Hepatitis E Virus Capsid by Transient Expression in *Nicotiana benthamiana*. Life.

[132] Chen Q., He J., Phoolcharoen W., Mason H.S. (2011). Geminiviral vectors based on bean yellow dwarf virus for production of vaccine antigens and monoclonal antibodies in plants. Hum. Vaccines..

[133] Kim M.Y., Reljic R., Kilbourne J., Ceballos-Olvera I., Yang M.S., Reyes-del Valle J., Mason H.S. (2015). Novel vaccination approach for dengue infection based on recombinant immune complex universal platform. Vaccine.

[134] Diamos A.G., Pardhe M.D., Sun H., Hunter J.G.L., Kilbourne J., Chen Q., Mason H.S. (2020). A Highly Expressing, Soluble, and Stable Plant-Made IgG Fusion Vaccine Strategy Enhances Antigen Immunogenicity in Mice Without Adjuvant. Front. Immunol..

[135] Diamos A.G., Pardhe M.D., Sun H., Hunter J.G.L., Mor T., Meador L., Kilbourne J., Chen Q., Mason H.S. (2020). Codelivery of improved immune complex and virus-like particle vaccines containing Zika virus envelope domain III synergistically enhances immunogenicity. Vaccine.

[136] Diamos A.G., Larios D., Brown L., Kilbourne J., Kim H.S., Saxena D. (2019). Vaccine synergy with virus-like particle and immune complex platforms for delivery of human papillomavirus L2 antigen. Vaccine.

[137] D’Aoust M.-A., Lavoie P.-O., Couture M.M., Trépanier S., Guay J.-M., Dargis M., Mongrand S., Landry N., Ward B.J., Vézina L.-P. (2008). Influenza virus-like particles produced by transient expression in Nicotiana benthamiana induce a protective immune response against a lethal viral challenge in mice. Plant Biotechnol. J..

[138] Pillet S., Couillard J., Trépanier S., Poulin J.F., Yassine-Diab B., Guy B., Ward B.J., Landry N. (2019). Immunogenicity and safety of a quadrivalent plant-derived virus like particle influenza vaccine candidate-Two randomized Phase II clinical trials in 18 to 49 and ≥50 years old adults. PLoS ONE.

[139] Ward B.J., Makarkov A., Séguin A., Pillet S., Trépanier S., Dhaliwall J., Libman M.D., Vesikari T., Landry N. (2020). Efficacy, immunogenicity, and safety of a plant-derived, quadrivalent, virus-like particle influenza vaccine in adults (18–64 years) and older adults (≥65 years): Two multicentre, randomised phase 3 trials. Lancet.

[140] Ward B.J., Séguin A., Couillard J., Trépanier S., Landry N. (2021). Phase III: Randomized observer-blind trial to evaluate lot-to-lot consistency of a new plant-derived quadrivalent virus like particle influenza vaccine in adults 18-49 years of age. Vaccine.

[141] Benvenuto E., Broer I., D’Aoust M.A., Hitzeroth I., Hundleby P., Menassa R., Oksman-Caldentey K.-M., Peyret H., Salgueiro S., Saxena P. (2023). Plant molecular farming in the wake of the closure of Medicago Inc. Nat. Biotechnol..

[142] Kirby T. (2023). Philip Morris ejected from Canadian vaccine collaboration. Lancet Respir. Med..

[143] Houston A.R. (2024). Medicago saga highlights need for WHO clarity on medical R&D involving tobacco. Can. J. Public Health.

[144] Royal J.M., Simpson C.A., McCormick A.A., Phillips A., Hume S., Morton J., Shepherd J., Oh Y., Swope K., DeBeauchamp J.L. (2021). Development of a SARS-CoV-2 Vaccine Candidate Using Plant-Based Manufacturing and a Tobacco Mosaic Virus-like Nano-Particle. Vaccines.

[145] Nikitin N., Vasiliev Y., Kovalenko A., Ryabchevskaya E., Kondakova O., Evtushenko E., Karpova O. (2023). Plant Viruses as Adjuvants for Next-Generation Vaccines and Immunotherapy. Vaccines.

[146] Kondakova O.A., Nikitin N.A., Trifonova E.A., Atabekov J.G., Karpova O.V. (2017). Rotavirus vaccines: New strategies and approaches. Moscow Univ. Biol.Sci. Bull..

[147] Kurokawa N., Lavoie P.-O., D’Aoust M.-A., Couture M.M., Dargis M., Trépanier S., Hoshino S., Koike T., Arai M., Tsutsui N. (2021). Development and characterization of a plant-derived rotavirus-like particle vaccine. Vaccine.

[148] Kurokawa N., Robinson M.K., Bernard C., Kawaguchi Y., Koujin Y., Koen A., Madhi S., Polasek T.M., McNeal M., Dargis M. (2021). Safety and immunogenicity of a plant-derived rotavirus-like particle vaccine in adults, toddlers and infants. Vaccine.

[149] Inoue J., Sato K., Ninomiya M., Masamune A. (2021). Envelope Proteins of Hepatitis B Virus: Molecular Biology and Involvement in Carcinogenesis. Viruses.

[150] Lazarevic I., Banko A., Miljanovic D., Cupic M. (2024). Hepatitis B Surface Antigen Isoforms: Their Clinical Implications, Utilisation in Diagnosis, Prevention and New Antiviral Strategies. Pathogens.

[151] Vesikari T., Finn A., van Damme P., Leroux-Roels I., Leroux-Roels G., Segall N., Toma A., Vallieres G., Aronson R., Reich D. (2021). Immunogenicity and Safety of a 3-Antigen Hepatitis B Vaccine vs a Single-Antigen Hepatitis B Vaccine: A Phase 3 Randomized Clinical Trial. JAMA Netw. Open..

[152] Vesikari T., Langley J.M., Segall N., Ward B.J., Cooper C., Poliquin G., Smith B., Gantt S., McElhaney J.E., Dionne M. (2021). Immunogenicity and safety of a tri-antigenic versus a mono-antigenic hepatitis B vaccine in adults (PROTECT): A randomised, double-blind, phase 3 trial. Lancet Infect. Dis..

[153] Dobrica M.-O., Lazar C., Paruch L., Skomedal H., Steen H., Haugslien S., Tucureanu C., Caras I., Onu A., Ciulean S. (2017). A novel chimeric Hepatitis B virus S/preS1 antigen produced in mammalian and plant cells elicits stronger humoral and cellular immune response than the standard vaccine-constituent, S protein. Antivir. Res..

[154] Dobrica M.-O., Lazar C., Paruch L., van Eerde A., Clarke J.L., Tucureanu C., Caras I., Ciulean S., Onu A., Tofan V. (2018). Oral administration of a chimeric Hepatitis B Virus S/preS1 antigen produced in lettuce triggers infection neutralizing antibodies in mice. Vaccine.

[155] Pantazica A.-M., Dobrica M.-O., Lazar C., Scurtu C., Tucureanu C., Caras I., Ionescu I., Costache A., Onu A., Clarke J.L. (2022). Efficient cellular and humoral immune response and production of virus-neutralizing antibodies by the Hepatitis B Virus S/preS116-42 antigen. Front. Immunol..

[156] Pantazica A.-M., van Eerde A., Dobrica M.-O., Caras I., Ionescu I., Costache A., Tucureanu C., Steen H., Lazar C., Heldal I. (2023). The “humanized” N-glycosylation pathway in CRISPR/Cas9-edited Nicotiana benthamiana significantly enhances the immunogenicity of a S/preS1 Hepatitis B Virus antigen and the virus-neutralizing antibody response in vaccinated mice. Plant Biotechnol. J..

[157] Jansing J., Sack M., Augustine S.M., Fischer R., Bortesi L. (2019). CRISPR/Cas9-mediated knockout of six glycosyltransferase genes in Nicotiana benthamiana for the production of recombinant proteins lacking β-1,2-xylose and core α-1,3-fucose. Plant Biotechnol. J..

[158] Ströh L.J., Krey T. (2020). HCV Glycoprotein Structure and Implications for B-Cell Vaccine Development. Int. J. Mol. Sci..

[159] Pantazica A.M., Cucos L.M., Stavaru C., Clarke J.L., Branza-Nichita N. (2021). Challenges and Prospects of Plant-Derived Oral Vaccines against Hepatitis B and C Viruses. Plants.

[160] Dobrica M.-O., van Eerde A., Tucureanu C., Onu A., Paruch L., Caras I., Vlase E., Steen H., Haugslien S., Alonzi D. (2021). Hepatitis C virus E2 envelope glycoprotein produced in Nicotiana benthamiana triggers humoral response with virus-neutralizing activity in vaccinated mice. Plant Biotechnol. J..

[161] Blokhina E.A., Mardanova E.S., Stepanova L.A., Tsybalova L.M., Ravin N.V. (2020). Plant-Produced Recombinant Influenza A Virus Candidate Vaccine Based on Flagellin Linked to Conservative Fragments of M2 Protein and Hemagglutintin. Plants.

[162] Blokhina E.A., Mardanova E.S., Zykova A.A., Stepanova L.A., Shuklina M.A., Tsybalova L.M., Ravin N.V. (2023). Plant-Produced Nanoparticles Based on Artificial Self-Assembling Peptide Bearing the Influenza M2e Epitope. Plants.

[163] Pang E.L., Peyret H., Ramirez A., Loh H.S., Lai K.S., Fang C.M., Rosenberg W.M., Lomonossoff G.P. (2019). Epitope Presentation of Dengue Viral Envelope Glycoprotein Domain III on Hepatitis B Core Protein Virus-Like Particles Produced in *Nicotiana benthamiana*. Front. Plant Sci..

[164] Stander J., Chabeda A., Rybicki E.P., Meyers A.E. (2021). A Plant-Produced Virus-Like Particle Displaying Envelope Protein Domain III Elicits an Immune Response Against West Nile Virus in Mice. Front. Plant Sci..

[165] Margolin E., Chapman R., Meyers A.E., van Diepen M.T., Ximba P., Hermanus T., Crowther C., Weber B., Morris L., Williamson A.-L. (2019). Production and Immunogenicity of Soluble Plant-Produced HIV-1 Subtype C Envelope gp140 Immunogens. Front. Plant Sci..

[166] Margolin E., Allen J.D., Verbeek M., Chapman R., Meyers A., van Diepen M., Ximba P., Motlou T., Moore P.L., Woodward J. (2022). Augmenting glycosylation-directed folding pathways enhances the fidelity of HIV Env immunogen production in plants. Biotechnol. Bioeng..

[167] Legastelois I., Buffin S., Peubez I., Mignon C., Sodoyer R., Werle B. (2017). Non-conventional expression systems for the production of vaccine proteins and immunotherapeutic molecules. Hum. Vaccin. Immunother..

[168] Rockstroh A., Moges B., Berneck B.S., Sattler T., Revilla-Fernández S., Schmoll F., Pacenti M., Sinigaglia A., Barzon L., Schmidt-Chanasit J. (2019). Specific detection and differentiation of tick-borne encephalitis and West Nile virus induced IgG antibodies in humans and horses. Transbound. Emerg. Dis..

[169] Weiß R., Issmail L., Rockstroh A., Grunwald T., Fertey J., Ulbert S. (2023). Immunization with different recombinant West Nile virus envelope proteins induces varying levels of serological cross-reactivity and protection from infection. Front. Cell. Infect. Microbiol..

[170] Oers M.M., Pijlman G.P., Vlak J.M. (2015). Thirty years of baculovirus-insect cell protein expression: From dark horse to mainstream technology. J. Gen. Virol..

[171] Cox M.M. (2012). Recombinant protein vaccines produced in insect cells. Vaccine.

[172] Rodríguez-Hernández A.P., Martínez-Flores D., Cruz-Reséndiz A., Padilla-Flores T., González-Flores R., Estrada K., Sampieri A., Camacho-Zarco A.R., Vaca L. (2023). Baculovirus Display of Peptides and Proteins for Medical Applications. Viruses.

[173] Luckow V.A., Lee S.C., Barry G.F., Olins P.O. (1993). Efficient generation of infectious recombinant baculoviruses by site-specific transposon-mediated insertion of foreign genes into a baculovirus genome propagated in *Escherichia coli*. J. Virol..

[174] Hong M., Li T., Xue W., Zhang S., Cui L., Wang H., Zhang Y., Zhou L., Gu Y., Xia N. (2022). Genetic engineering of baculovirus-insect cell system to improve protein production. Front. Bioeng. Biotechnol..

[175] Weyer U., Possee R.D. (1991). A baculovirus dual expression vector derived from the Autographa californica nuclear polyhedrosis virus polyhedrin and p10 promoters: Co-expression of two influenza virus genes in insect cells. J. Gen. Virol..

[176] Wells D.E., Compans R.W. (1990). Expression and characterization of a functional human immunodeficiency virus envelope glycoprotein in insect cells. Virology.

[177] Mohan T., Berman Z., Kang S.M., Wang B.Z. (2018). Sequential immunizations with a panel of HIV-1 Env virus-like particles coach immune system to make broadly neutralizing antibodies. Sci. Rep..

[178] Grohskopf L.A., Blanton L.H., Ferdinands J.M., Chung J.R., Broder K.R., Talbot H.K. (2023). Prevention and Control of Seasonal Influenza with Vaccines: Recommendations of the Advisory Committee on Immunization Practices—United States, 2023–2024 Influenza Season. MMWR Recomm. Rep..

[179] Cox M.M., Hollister J.R. (2009). FluBlok, a next generation influenza vaccine manufactured in insect cells. Biologicals.

[180] Dawood F.S., Naleway A.L., Flannery B., Levine M.Z., Murthy K., Sambhara S., Gangappa S., Edwards L., Ball S., Grant L. (2021). Comparison of the Immunogenicity of Cell Culture-Based and Recombinant Quadrivalent Influenza Vaccines to Conventional Egg-Based Quadrivalent Influenza Vaccines Among Healthcare Personnel Aged 18-64 Years: A Randomized Open-Label Trial. Clin. Infect. Dis..

[181] Gil A., Sinilaite A., Papenburg J. (2022). Summary of the National Advisory Committee on Immunization (NACI) Supplemental Statement on Recombinant Influenza Vaccines. Can. Commun. Dis. Rep..

[182] Liu F., Gross F.L., Joshi S., Gaglani M., Naleway A.L., Murthy K., Groom H.C., Wesley M.G., Edwards L.J., Grant L. (2024). Redirecting antibody responses from egg-adapted epitopes following repeat vaccination with recombinant or cell culture-based versus egg-based influenza vaccines. Nat. Commun..

[183] Wang Y., Li S., Dong C., Ma Y., Song Y., Zhu W., Kim J., Deng L., Denning T.L., Kang S.-M. (2021). Skin vaccination with dissolvable microneedle patches incorporating influenza neuraminidase and flagellin protein nanoparticles induces broad immune protection against multiple influenza viruses. ACS Appl. Bio. Mater..

[184] Wang Y., Dong C., Ma Y., Zhu W., Gill H.S., Denning T.L., Kang S.-M., Wang B.-Z. (2023). Monophosphoryl lipid A-adjuvanted nucleoprotein-neuraminidase nanoparticles improve immune protection against divergent influenza viruses. Nanomedicine.

[185] Kim M.C., Song J.M., Eunju O., Kwon Y.M., Lee Y.J., Compans R.W., Kang S.M. (2013). Virus-like Particles Containing Multiple M2 Extracellular Domains Confer Improved Cross-protection Against Various Subtypes of Influenza Virus. Mol. Ther..

[186] Lee Y.T., Kim K.H., Ko E.J., Kim M.C., Lee Y.N., Hwang H.S., Lee Y., Jung Y.J., Kim Y.J., Santos J. (2019). Enhancing the cross protective efficacy of live attenuated influenza virus vaccine by supplemented vaccination with M2 ectodomain virus-like particles. Virology.

[187] Kim M.C., Kim K.H., Lee J.W., Lee Y.N., Choi H.J., Jung Y.J., Kim Y.J., Compans R.W., Prausnitz M.R., Kang S.M. (2019). Co-Delivery of M2e Virus-Like Particles with Influenza Split Vaccine to the Skin Using Microneedles Enhances the Efficacy of Cross Protection. Pharmaceutics.

[188] Gomes K., D’Sa S., Allotey-Babington G.L., Kang S.-M., D’Souza M.J. (2021). Transdermal Vaccination with the Matrix-2 Protein Virus-like Particle (M2e VLP) Induces Immunity in Mice against Influenza A Virus. Vaccines.

[189] Gomes K.B., Menon I., Bagwe P., Bajaj L., Kang S.-M., D’Souza M.J. (2022). Enhanced Immunogenicity of an Influenza Ectodomain Matrix-2 Protein Virus-like Particle (M2e VLP) Using Polymeric Microparticles for Vaccine Delivery. Viruses.

[190] Gomes K., Vijayanand S., Bagwe P., Menon I., Kale A., Patil S., Kang S.-M., Uddin M.N., D’Souza M.J. (2023). Vaccine-Induced Immunity Elicited by Microneedle Delivery of Influenza Ectodomain Matrix Protein 2 Virus-like Particle (M2e VLP)-Loaded PLGA Nanoparticles. Int. J. Mol. Sci..

[191] Kim K.H., Li Z., Bhatnagar N., Subbiah J., Park B.R., Shin C.H., Pushko P., Wang B.-Z., Kang S.-M. (2022). Universal protection against influenza viruses by multi-subtype neuraminidase and M2 ectodomain virus-like particle. PLoS Pathog..

[192] Raha J.R., Kim K.-H., Bhatnagar N., Liu R., Le C.T.T., Park B.R., Grovenstein P., Pal S.S., Ko E.-J., Shin C.H. (2024). Supplementation of seasonal vaccine with multi-subtype neuraminidase and M2 ectodomain virus-like particle improves protection against homologous and heterologous influenza viruses in aged mice. Antiviral Res..

[193] Kostina L.V., Filatov I.E., Eliseeva O.V., Latyshev O.E., Chernoryzh Y.Y., Yurlov K.I., Lesnova E.I., Khametova K.M., Cherepushkin S.A., Savochkina T.E. (2023). [Study of the safety and immunogenicity of VLP-based vaccine for the prevention of rotavirus infection in neonatal minipig model]. Vopr. Virusol..

[194] Cherepushkin S.A., Tsibezov V.V., Yuzhakov A.G., Latyshev O.E., Alekseev K.P., Altayeva E.G., Khametova K.M., Vorkunova G.K., Yuzhakova K.A., Grebennikova T.V. (2021). [Synthesis and characterization of human rotavirus A (Reoviridae: Sedoreovirinae: Rotavirus: Rotavirus A) virus-like particles]. Vopr. Virusol..

[195] Griffiths C., Drews S.J., Marchant D.J. (2017). Respiratory Syncytial Virus: Infection, Detection, and New Options for Prevention and Treatment. Clin. Microbiol. Rev..

[196] Anderson C.S., Chu C.-Y., Wang Q., Mereness J.A., Ren Y., Donlon K., Bhattacharya S., Misra R.S., Walsh E.E., Pryhuber G.S. (2020). CX3CR1 as a respiratory syncytial virus receptor in pediatric human lung. Pediatr. Res..

[197] Crank M.C., Ruckwardt T.J., Chen M., Morabito K.M., Phung E., Costner P.J., Holman L.A., Hickman S.P., Berkowitz N.M., Gordon I.J. (2019). A proof of concept for structure-based vaccine design targeting RSV in humans. Science.

[198] Luo J., Qin H., Lei L., Lou W., Li R., Pan Z. (2022). Virus-like particles containing a prefusion-stabilized F protein induce a balanced immune response and confer protection against respiratory syncytial virus infection in mice. Front. Immunol..

[199] Chu K.-B., Lee S.-H., Kim M.-J., Kim A.-R., Moon E.-K., Quan F.-S. (2022). Virus-like particles coexpressing the PreF and Gt antigens of respiratory syncytial virus confer protection in mice. Nanomedicine.

[200] Lee S.-H., Chu K.-B., Kim M.-J., Mao J., Eom G.-D., Yoon K.-W., Ahmed M.A., Quan F.-S. (2023). Virus-like Particle Vaccine Expressing the Respiratory Syncytial Virus Pre-Fusion and G Proteins Confers Protection against RSV Challenge Infection. Pharmaceutics.

[201] Kim M.-J., Chu K.B., Lee S.-H., Mao J., Eom G.-D., Yoon K.-W., Moon E.-K., Quan F.-S. (2024). Assessing the protection elicited by virus-like particles expressing the RSV pre-fusion F and tandem repeated G proteins against RSV rA2 line19F infection in mice. Respir. Res..

[202] Lee S.H., Chu K.B., Kim M.J., Quan F.S. (2023). Virus-Like Particles Assembled Using Respiratory Syncytial Virus Matrix Protein Elicit Protective Immunity in Mice. Infect. Drug Resist..

[203] Woolsey C., Fears A.C., Borisevich V., Agans K.N., Dobias N.S., Prasad A.N., Deer D.J., Geisbert J.B., Fenton K.A., Geisbert T.W. (2022). Natural history of Sudan ebolavirus infection in rhesus and cynomolgus macaques. Emerg. Microbes Infect..

[204] Malik S., Kishore S., Nag S., Dhasmana A., Preetam S., Mitra O., León-Figueroa D.A., Mohanty A., Chattu V.K., Assefi M. (2023). Ebola Virus Disease Vaccines: Development, Current Perspectives & Challenges. Vaccines.

[205] Bengtsson K.L., Song H., Stertman L., Liu Y., Flyer D.C., Massare M.J., Xu R.-H., Zhou B., Lu H., Kwilas S.A. (2016). Matrix-M adjuvant enhances antibody, cellular and protective immune responses of a Zaire Ebola/Makona virus glycoprotein (GP) nanoparticle vaccine in mice. Vaccine.

[206] Fries L., Cho I., Krähling V., Fehling S.K., Strecker T., Becker S., Hooper J.W., Kwilas S.A., Agrawal S., Wen J. (2020). Randomized, Blinded, Dose-Ranging Trial of an Ebola Virus Glycoprotein Nanoparticle Vaccine with Matrix-M Adjuvant in Healthy Adults. J. Infect. Dis..

[207] Wu F., Zhang S., Zhang Y., Mo R., Yan F., Wang H., Wong G., Chi H., Wang T., Feng N. (2020). A Chimeric Sudan Virus-Like Particle Vaccine Candidate Produced by a Recombinant Baculovirus System Induces Specific Immune Responses in Mice and Horses. Viruses.

[208] Shinde V., Cho I., Plested J.S., Agrawal S., Fiske J., Cai R., Zhou H., Pham X., Zhu M., Cloney-Clark S. (2022). Comparison of the safety and immunogenicity of a novel Matrix-M-adjuvanted nanoparticle influenza vaccine with a quadrivalent seasonal influenza vaccine in older adults: A phase 3 randomised controlled trial. Lancet Infect. Dis.

[209] Wang Y., Deng L., Gonzalez G.X., Luthra L., Dong C., Ma Y., Zou J., Kang S.-M., Wang B.-Z. (2020). Double-Layered M2e-NA Protein Nanoparticle Immunization Induces Broad Cross-Protection against Different Influenza Viruses in Mice. Adv. Healthc. Mater..

[210] Lampinen V., Gröhn S., Soppela S., Blazevic V., Hytönen V.P., Hankaniemi M.M. (2023). SpyTag/SpyCatcher display of influenza M2e peptide on norovirus-like particle provides stronger immunization than direct genetic fusion. Front. Cell Infect. Microbiol..

[211] Liu C.-C., Liu D.-J., Yue X.-Y., Zhong X.-Q., Wu X., Chang H.-Y., Wang B.-Z., Wan M.-Y., Deng L. (2022). Chimeric Virus-like Particles Co-Displaying Hemagglutinin Stem and the C-Terminal Fragment of DnaK Confer Heterologous Influenza Protection in Mice. Viruses.

[212] Ma M., Xia B., Wang Z., Hao Y., Zhang T., Xu X. (2023). A novel C-terminal modification method enhanced the yield of human papillomavirus L1 or chimeric L1-L2 virus-like particles in the baculovirus system. Front. Bioeng. Biotechnol.

[213] Luo D., Miao Y., Ke X., Tan Z., Hu C., Li P., Wang T., Zhang Y., Sun J., Liu Y. (2020). Baculovirus Surface Display of Zika Virus Envelope Protein Protects against Virus Challenge in Mouse Model. Virol. Sin..

[214] Barone P.W., Wiebe M.E., Leung J.C., Hussein I.T.M., Keumurian F.J., Bouressa J., Brussel A., Chen D., Chong M., Dehghani H. (2020). Viral contamination in biologic manufacture and implications for emerging therapies. Nat. Biotechnol..

[215] Toth M., Reithofer M., Dutra G., Pereira Aguilar P., Dürauer A., Grabherr R. (2024). Comprehensive Comparison of Baculoviral and Plasmid Gene Delivery in Mammalian Cells. Viruses.

[216] Gaillet B., Gilbert R., Broussau S., Pilotte A., Malenfant F., Mullick A., Garnier A., Massie B. (2010). High-level recombinant protein production in CHO cells using lentiviral vectors and the cumate gene-switch. Biotechnol. Bioeng..

[217] Lalonde M.-E., Durocher Y. (2017). Therapeutic glycoprotein production in mammalian cells. J. Biotechnol..

[218] Sandig G., von Horsten H.H., Radke L., Blanchard V., Frohme M., Giese C., Sandig V., Hinderlich S. (2017). Engineering of CHO Cells for the Production of Recombinant Glycoprotein Vaccines with Xylosylated N-glycans. Bioengineering.

[219] Byrne G., O’Rourke S.M., Alexander D.L., Yu B., Doran R.C., Wright M., Chen Q., Azadi P., Berman P.W. (2018). CRISPR/Cas9 gene editing for the creation of an MGAT1-deficient CHO cell line to control HIV-1 vaccine glycosylation. PLoS Biol..

[220] Donini R., Haslam S.M., Kontoravdi C. (2021). Glycoengineering Chinese hamster ovary cells: A short history. Biochem. Soc. Trans..

[221] Geng S.L., Zhao X.J., Zhang X., Zhang J.H., Mi C.L., Wang T.Y. (2024). Recombinant therapeutic proteins degradation and overcoming strategies in CHO cells. Appl. Microbiol. Biotechnol..

[222] Li S.W., Yu B., Byrne G., Wright M., O’Rourke S., Mesa K., Berman P.W. (2019). Identification and CRISPR/Cas9 Inactivation of the C1s Protease Responsible for Proteolysis of Recombinant Proteins Produced in CHO Cells. Biotechnol. Bioeng..

[223] Li S.W., Wright M., Healey J.F., Hutchinson J.M., O’Rourke S., Mesa K.A., Lollar P., Berman P.W. (2020). Gene editing in CHO cells to prevent proteolysis and enhance glycosylation: Production of HIV envelope proteins as vaccine immunogens. PLoS ONE.

[224] Tan E., Chin C.S.H., Lim Z.F.S., Ng S.K. (2021). HEK293 Cell Line as a Platform to Produce Recombinant Proteins and Viral Vectors. Front. Bioeng. Biotechnol..

[225] Walsh G., Walsh E. (2022). Biopharmaceutical benchmarks 2022. Nat. Biotechnol..

[226] Chen T.H., Liu W.C., Lin C.Y., Liu C.C., Jan J.T., Spearman M., Butler M., Wu S.-C. (2019). Glycan-masking hemagglutinin antigens from stable CHO cell clones for H5N1 avian influenza vaccine development. Biotechnol. Bioeng..

[227] Lin S.C., Lin Y.F., Chong P., Wu S.C. (2012). Broader neutralizing antibodies against H5N1 viruses using prime-boost immunization of hyperglycosylated hemagglutinin DNA and virus-like particles. PLoS ONE.

[228] Lin S.C., Liu W.-C., Jan J.T., Wu S.C. (2014). Glycan masking of hemagglutinin for adenovirus vector and recombinant protein immunizations elicits broadly neutralizing antibodies against H5N1 avian influenza viruses. PLoS ONE.

[229] Thoresen D., Matsuda K., Urakami A., Ngwe Tun M.M., Nomura T., Moi M.L., Watanabe Y., Ishikawa M., Hau T.T.T., Yamamoto H. (2024). A tetravalent dengue virus-like particle vaccine induces high levels of neutralizing antibodies and reduces dengue replication in non-human primates. J. Virol..

[230] De Lorenzo G., Tandavanitj R., Doig J., Setthapramote C., Poggianella M., Sanchez-Velazquez R., Scales H.E., Edgar J.M., Kohl A., Brewer J. (2020). Zika Virus-Like Particles Bearing a Covalent Dimer of Envelope Protein Protect Mice from Lethal Challenge. J. Virol..

[231] Mancini M.V., Tandavanitj R., Ant T.H., Murdochy S.M., Gingell D.D., Setthapramote C., Natsrita P., Kohl A., Sinkins S.P., Patel A.H. (2023). Evaluation of an Engineered Zika Virus-Like Particle Vaccine Candidate in a Mosquito-Mouse Transmission Model. mSphere..

[232] Vang L., Morello C.S., Mendy J., Thompson D., Manayani D., Guenther B., Julander J., Sanford D., Jain A., Patel A. (2021). Zika virus-like particle vaccine protects AG129 mice and rhesus macaques against Zika virus. PLoS Negl. Trop. Dis..

[233] Tai W., Chen J., Zhao G., Geng Q., He L., Chen Y., Zhou Y., Li F., Du L. (2019). Rational Design of Zika Virus Subunit Vaccine with Enhanced Efficacy. J. Virol..

[234] Su H., Liu J., Yu J., Qiu Z., Liang W., Wu W., Mo H., Li H., Zhao W., Gu W. (2023). EDIII-Fc induces protective immune responses against the Zika virus in mice and rhesus macaque. PLoS Negl. Trop. Dis..

[235] Dendouga N., Fochesato M., Lockman L., Mossman S., Giannini S.L. (2012). Cell-mediated immune responses to a varicella-zoster virus glycoprotein E vaccine using both a TLR agonist and QS21 in mice. Vaccine.

[236] Wang Y., Qi J., Cao H., Liu C. (2021). Immune Responses to Varicella-Zoster Virus Glycoprotein E Formulated with Poly(Lactic-co-Glycolic Acid) Nanoparticles and Nucleic Acid Adjuvants in Mice. Virol. Sin..

[237] Cao H., Wang Y., Luan N., Liu C. (2021). Immunogenicity of Varicella-Zoster Virus Glycoprotein E Formulated with Lipid Nanoparticles and Nucleic Immunostimulators in Mice. Vaccines.

[238] Luan N., Cao H., Wang Y., Lin K., Liu C. (2022). LNP-CpG ODN-adjuvanted varicella-zoster virus glycoprotein E induced comparable levels of immunity with Shingrix™ in VZV-primed mice. Virol. Sin..

[239] Wui S.R., Kim K.S., Ryu J.I., Ko A., Do H.T.T., Lee Y.J., Kim H.J., Lim S.J., Park S.A., Cho Y.J. (2019). Efficient induction of cell-mediated immunity to varicella-zoster virus glycoprotein E co-lyophilized with a cationic liposome-based adjuvant in mice. Vaccine.

[240] Kim K.S., Park S.A., Wui S.R., Ko A., Lee N.G. (2021). Culture media optimization for Chinese hamster ovary cell growth and expression of recombinant varicella-zoster virus glycoprotein E. Cytotechnology.

[241] Shouval D., Roggendorf H., Roggendorf M. (2015). Enhanced immune response to hepatitis B vaccination through immunization with a Pre-S1/Pre-S2/S vaccine. Med. Microbiol. Immunol..

[242] Vesikari T., Langley J.M., Popovic V., Diaz-Mitoma F. (2023). PreHevbrio: The first approved 3-antigen hepatitis B vaccine. Expert. Rev. Vaccines..

[243] Talbird S.E., Anderson S.A., Nossov M., Beattie N., Rak A.T., Diaz-Mitoma F. (2023). Cost-effectiveness of a 3-antigen versus single-antigen vaccine for the prevention of hepatitis B in adults in the United States. Vaccine.

[244] Pierce B.G., Keck Z.-Y., Wang R., Lau P., Garagusi K., Elkholy K., Toth E.A., Urbanowicz R.A., Guest J.D., Agnihotri P. (2020). Structure-Based Design of Hepatitis C Virus E2 Glycoprotein Improves Serum Binding and Cross-Neutralization. J. Virol..

[245] He L., Tzarum N., Lin X., Shapero B., Sou C., Mann C.J., Stano A., Zhang L., Nagy K., Giang E. (2020). Proof of concept for rational design of hepatitis C virus E2 core nanoparticle vaccines. Sci. Adv..

[246] McGregor J., Hardy J.M., Lay C.-S., Boo I., Piontek M., Suckow M., Coulibaly F., Poumbourios P., Center R.J., Drummer H.E. (2022). Virus-Like Particles Containing the E2 Core Domain of Hepatitis C Virus Generate Broadly Neutralizing Antibodies in Guinea Pigs. J. Virol..

[247] Wang R., Suzuki S., Guest J.D., Heller B., Almeda M., Andrianov A.K., Marin A., Mariuzza R.A., Keck Z.-Y., Foung S.K.H. (2022). Induction of broadly neutralizing antibodies using a secreted form of the hepatitis C virus E1E2 heterodimer as a vaccine candidate. Proc. Natl. Acad. Sci. USA.

[248] Sliepen K., Radić L., Capella-Pujol J., Watanabe Y., Zon I., Chumbe A., Lee W.-H., de Gast M., Koopsen J., Koekkoek S. (2022). Induction of cross-neutralizing antibodies by a permuted hepatitis C virus glycoprotein nanoparticle vaccine candidate. Nat. Commun..

[249] Prabakaran P., Dimitrov A.S., Fouts T.R., Dimitrov D.S. (2007). Structure and function of the HIV envelope glycoprotein as entry mediator, vaccine immunogen, and target for inhibitors. Adv. Pharmacol..

[250] Haynes B.F., Wiehe K., Borrow P., Saunders K.O., Korber B., Wagh K., McMichael A.J., Kelsoe G., Hahn B.H., Alt F. (2023). Strategies for HIV-1 vaccines that induce broadly neutralizing antibodies. Nat. Rev. Immunol..

[251] Ward A.B., Wilson I.A. (2017). The HIV-1 envelope glycoprotein structure: Nailing down a moving target. Immunol. Rev..

[252] Kwon Y.D., Pancera M., Acharya P., Georgiev I.S., Crooks E.T., Gorman J., Joyce M.G., Guttman M., Ma X., Narpala S. (2015). Crystal structure, conformational fixation and entry-related interactions of mature ligand-free HIV-1 Env. Nat. Struct. Mol. Biol..

[253] Houser K.V., Gaudinski M.R., Happe M., Narpala S., Verardi R., Sarfo E.K., Corrigan A.R., Wu R., Rothwell R.S., Novik L. (2022). Safety and immunogenicity of an HIV-1 prefusion-stabilized envelope trimer (Trimer 4571) vaccine in healthy adults: A first-in-human open-label, randomized, dose-escalation, phase 1 clinical trial. EClinicalMedicine.

[254] Hu W.S., Temin H.M. (1990). Retroviral recombination and reverse transcription. Science.

[255] Levy D.N., Aldrovandi G.M., Kutsch O., Shaw G.M. (2004). Dynamics of HIV-1 recombination in its natural target cells. Proc. Natl. Acad. Sci. USA.

[256] Hargrave A., Mustafa A.S., Hanif A., Tunio J.H., Hanif S.N.M. (2021). Current Status of HIV-1 Vaccines. Vaccines.

[257] Zhang Y.N., Paynter J., Antanasijevic A., Allen J.D., Eldad M., Lee Y.Z., Copps J., Newby M.L., He L., Chavez D. (2023). Single-component multilayered self-assembling protein nanoparticles presenting glycan-trimmed uncleaved prefusion optimized envelope trimmers as HIV-1 vaccine candidates. Nat. Commun..

[258] Koornneef A., Vanshylla K., Hardenberg G., Rutten L., Strokappe N.M., Tolboom J., Vreugdenhil J., Boer K.F., Perkasa A., Blokland S. (2024). CoPoP liposomes displaying stabilized clade C HIV-1 Env elicit tier 2 multiclade neutralization in rabbits. Nat. Commun..

[259] Sharma V.K., Menis S., Brower E.T., Sayeed E., Ackland J., Lombardo A., Cottrell C.A., Torres J.L., Hassell T., Ward A.B. (2024). Use of Transient Transfection for cGMP Manufacturing of eOD-GT8 60mer, a Self-Assembling Nanoparticle Germline-Targeting HIV-1 Vaccine Candidate. Pharmaceutics.

[260] Leggat D.J., Cohen K.W., Willis J.R., Fulp W.J., deCamp A.C., Kalyuzhniy O., Cottrell C.A., Menis S., Finak G., Ballweber-Fleming L. (2022). Vaccination induces HIV broadly neutralizing antibody precursors in humans. Science.

[261] Cohen K.W., De Rosa S.C., Fulp W.J., deCamp A.C., Fiore-Gartland A., Mahoney C.R., Furth S., Donahue J., Whaley R.E., Ballweber-Fleming L. (2023). A first-in-human germline-targeting HIV nanoparticle vaccine induced broad and publicly targeted helper T cell responses. Sci. Transl. Med..

[262] Ruckwardt T.J., Morabito K.M., Phung E., Crank M.C., Costner P.J., Holman L.A., Chang L.A., Hickman S.P., Berkowitz N.M., Gordon I.J. (2021). Safety, tolerability, and immunogenicity of the respiratory syncytial virus prefusion F subunit vaccine DS-Cav1: A phase 1, randomised, open-label, dose-escalation clinical trial. Lancet Respir. Med..

[263] Lee Y.Z., Han J., Zhang Y.N., Ward G., Gomes K.B., Auclair S., Stanfield R.L., He L., Wilson I.A., Zhu J. (2024). A tale of two fusion proteins: Understanding the metastability of human respiratory syncytial virus and metapneumovirus and implications for rational design of uncleaved prefusion-closed trimers. bioRxiv.

[264] Pang Y., Lu H., Cao D., Zhu X., Long Q., Tian F., Long X., Li Y. (2024). Efficacy, immunogenicity and safety of respiratory syncytial virus prefusion F vaccine: Systematic review and meta-analysis. BMC Public Health.

[265] McGinnes Cullen L., Luo B., Wen Z., Zhang L., Durr E., Morrison T.G. (2023). The Respiratory Syncytial Virus (RSV) G Protein Enhances the Immune Responses to the RSV F Protein in an Enveloped Virus-Like Particle Vaccine Candidate. J. Virol..

[266] Voorzaat R., Cox F., van Overveld D., Le L., Tettero L., Vaneman J., Bakkers M.J.G., Langedijk J.P.M. (2024). Design and Preclinical Evaluation of a Nanoparticle Vaccine against Respiratory Syncytial Virus Based on the Attachment Protein G. Vaccines.

[267] Xu D., Powell A.E., Utz A., Sanyal M., Do J., Patten J.J., Moliva J.I., Sullivan N.J., Davey R.A., Kim P.S. (2024). Design of universal Ebola virus vaccine candidates via immunofocusing. Proc. Natl. Acad. Sci. USA.

[268] Mesri E.A., Cesarman E., Boshoff C. (2010). Kaposi’s sarcoma and its associated herpesvirus. Nat. Rev. Cancer..

[269] Casper C., Corey L., Cohen J.I., Damania B., Gershon A.A., Kaslow D.C., Krug L.T., Martin J., Mbulaiteye S.M., Mocarski E.S. (2022). KSHV (HHV8) vaccine: Promises and potential pitfalls for a new anti-cancer vaccine. NPJ Vaccines.

[270] Mulama D.H., Mutsvunguma L.Z., Totonchy J., Ye P., Foley J., Escalante G.M., Rodriguez E., Nabiee R., Muniraju M., Wussow F. (2019). A multivalent Kaposi sarcoma-associated herpesvirus-like particle vaccine capable of eliciting high titers of neutralizing antibodies in immunized rabbits. Vaccine.

[271] Farrell P.J. (2019). Epstein-Barr Virus and Cancer. Annu. Rev. Pathol..

[272] Bjornevik K., Cortese M., Healy B.C., Kuhle J., Mina M.J., Leng Y., Elledge S.J., Niebuhr D.W., Scher A.I., Munger K.L. (2022). Longitudinal analysis reveals high prevalence of Epstein-Barr virus associated with multiple sclerosis. Science.

[273] Dasari V., McNeil L.K., Beckett K., Solomon M., Ambalathingal G., Thuy T.L., Panikkar A., Smith C., Steinbuck M.P., Jakubowski A. (2023). Lymph node targeted multi-epitope subunit vaccine promotes effective immunity to EBV in HLA-expressing mice. Nat. Commun..

[274] Sokal E.M., Hoppenbrouwers K., Vandermeulen C., Moutschen M., Léonard P., Moreels A., Haumont M., Bollen A., Smets F., Denis M. (2007). Recombinant gp350 vaccine for infectious mononucleosis: A phase 2, randomized, double-blind, placebo-controlled trial to evaluate the safety, immunogenicity, and efficacy of an Epstein-Barr virus vaccine in healthy young adults. J. Infect. Dis..

[275] Bu W., Joyce M.G., Nguyen H., Banh D.V., Aguilar F., Tariq Z., Yap M.L., Tsujimura Y., Gillespie R.A., Tsybovsky Y. (2019). Immunization with Components of the Viral Fusion Apparatus Elicits Antibodies That Neutralize Epstein-Barr Virus in B Cells and Epithelial Cells. Immunity.

[276] Wei C.J., Bu W., Nguyen L.A., Batchelor J.D., Kim J., Pittaluga S., Fuller J.R., Nguyen H., Chou T.H., Cohen J.I. (2022). A bivalent Epstein-Barr virus vaccine induces neutralizing antibodies that block infection and confer immunity in humanized mice. Sci. Transl. Med..

[277] Escalante G.M., Foley J., Mutsvunguma L.Z., Rodriguez E., Mulama D.H., Muniraju M., Ye P., Barasa A.K., Ogembo J.G. (2020). A Pentavalent Epstein-Barr Virus-Like Particle Vaccine Elicits High Titers of Neutralizing Antibodies against Epstein-Barr Virus Infection in Immunized Rabbits. Vaccines.

[278] Malhi H., Homad L.J., Wan Y.-H., Poudel B., Fiala B., Borst A.J., Wang J.Y., Walkey C., Price J., Wall A. (2022). Immunization with a self-assembling nanoparticle vaccine displaying EBV gH/gL protects humanized mice against lethal viral challenge. Cell Rep. Med..

[279] U.S. Food and Drug Administration. https://www.fda.gov/vaccines-blood-biologics/prehevbrio.

[280] Lee C.Y.F., Khan S.J., Vishal F., Alam S., Murtaza S.F. (2023). Respiratory Syncytial Virus Prevention: A New Era of Vaccines. Cureus..

[281] Pfizer. https://www.pfizer.com/news/press-release/press-release-detail/us-fda-approves-abrysvotm-pfizers-vaccine-prevention.

[282] Swanson K.A., Rainho-Tomko J.N., Williams Z.P., Lanza L., Peredelchuk M., Kishko M., Pavot V., Alamares-Sapuay J., Adhikarla H., Gupta S. (2020). A respiratory syncytial virus (RSV) F protein nanoparticle vaccine focuses antibody responses to a conserved neutralization domain. Sci. Immunol..

[283] He L., Chaudhary A., Lin X., Sou C., Alkutkar T., Kumar S., Ngo T., Kosviner E., Ozorowski G., Stanfield R.L. (2021). Single-component multilayered self-assembling nanoparticles presenting rationally designed glycoprotein trimers as Ebola virus vaccines. Nat. Commun..

[284] Kawahara E., Shibata T., Hirai T., Yoshioka Y. (2023). Non-glycosylated G protein with CpG ODN provides robust protection against respiratory syncytial virus without inducing eosinophilia. Front. Immunol..

[285] Park J., Champion J.A. (2023). Development of Self-Assembled Protein Nanocage Spatially Functionalized with HA Stalk as a Broadly Cross-Reactive Influenza Vaccine Platform. ACS Nano.

[286] Qian G., Gao C., Zhang M., Chen Y., Xie L. (2024). A Review of Protein-Based COVID-19 Vaccines: From Monovalent to Multivalent Formulations. Vaccines.

